# Protocol for a single patient therapy plan: A randomised, double‐blind, placebo‐controlled N‐of‐1 trial to assess the efficacy of cannabidiol in patients with intractable epilepsy

**DOI:** 10.1111/jpc.15078

**Published:** 2020-09-23

**Authors:** Katherine S Ong, John B Carlin, Michael Fahey, Jeremy L Freeman, Ingrid E Scheffer, Lynn Gillam, Monique Anderson, Md Hamidul Huque, Donna Legge, Nicole Dirnbauer, Brian Lilley, Simon Slota‐Kan, Noel Cranswick

**Affiliations:** ^1^ Victoria Department of Health and Human Services Melbourne Victoria Australia; ^2^ Murdoch Children's Research Institute Melbourne Victoria Australia; ^3^ The University of Melbourne Melbourne Victoria Australia; ^4^ Monash Children's Hospital Melbourne Victoria Australia; ^5^ Royal Children's Hospital Melbourne Victoria Australia; ^6^ Austin Hospital Melbourne Victoria Australia; ^7^ Neuroscience Trials Australia Melbourne Victoria Australia

**Keywords:** anticonvulsant/therapeutic use, cannabidiol, child, drug resistant epilepsy, outcome assessment (health care)

## Abstract

**Aim:**

This paper describes the use of the single patient therapy plan (SPTP). The SPTP has been designed to assess the efficacy at an individual level of a commercially available cannabinoid product, cannabidiol, in reducing seizure frequency in paediatric patients with intractable epilepsy.

**Methods:**

The SPTP is a randomised, double‐blind, placebo‐controlled N‐of‐1 trial designed to assess the efficacy of treatment in a neurology outpatient setting. The primary objective of the SPTP is to assess the efficacy of cannabidiol in reducing seizure frequency in each patient with intractable epilepsy, with change in seizure frequency being the primary outcome of interest.

The analysis adopts a Bayesian approach, which provides results in the form of posterior probabilities that various levels of benefit (based on the primary outcome measure, seizure frequency) have been achieved under active treatment compared to placebo, accompanied by decision rules that provide thresholds for deciding whether treatment has been successful in the individual patient.

The SPTP arrangement is most accurately considered part of clinical practice rather than research, since it is aimed at making clinical treatment decisions for individual patients and is not testing a hypothesis or collecting aggregate data. Therefore, Human Research Ethics Committee approval was considered not to be required, although it is recommended that hospital Clinical Ethics Committees provide ethical oversight.

**Conclusion:** These SPTP resources are made available so that they may inform clinical practice in the treatment of severe epilepsy or adapted for use in other conditions.

## What is already known on this topic

1. An N‐of‐1 trial of therapy is a treatment model empowering patients and treating clinicians to make treatment decisions based on results seen in the individual patient.
2. The N‐of‐1 trial of therapy model requires cooperation between the treating clinician, pharmacy, statistician and support of ethics committees.
3. The N‐of‐1 trial of therapy requires the availability/manufacture of a matching placebo product.

## What this paper adds

1. This single patient therapy plan provides a decision tool for patients and treating clinicians in the setting of emerging data on the efficacy of cannabidiol for the treatment of severe paediatric epilepsy.
2. This single patient therapy plan can be followed for the patient population, treatment and indication provided, or the model and resources can be adapted for use in other settings.

Epilepsy is a neurological condition, characterised by recurrent seizures, which affects approximately 0.4% of the population.^1^ Conventional treatments including anti‐epileptic drugs (AEDs), surgery or ketogenic diet control seizures in up to 70% of patients.^2^ However, in approximately one third of patients, existing therapies do not adequately control symptoms, and there is a need for novel treatments in these patients.

Cannabidiol, a non‐psychoactive cannabinoid derived from the cannabis plant, has been shown to reduce seizures in patients with the Dravet syndrome and Lennox Gastaut syndrome when used as add‐on therapy with other antiepileptic treatments.^3^, ^4^ There is an Food and Drug Administration‐registered formulation available in the USA for these indications; however, evidence of the efficacy of cannabidiol in the treatment of other forms of epilepsy is lacking.

The recent legalisation of cannabis products for medicinal use in Australia has resulted in the availability of a range of unregistered medicinal cannabis products, including several formulations of cannabidiol. This change has led to an increase in patients with intractable epilepsy receiving cannabidiol as an add‐on therapy. However, the complexity of these patients, their diverse diagnoses, the multiple pharmaceutical and non‐pharmaceutical treatments they receive and the impact of other comorbidities, means that assessment of the efficacy of cannabidiol is challenging.

The need to ascertain individual patient efficacy is particularly pertinent when using long‐term treatments in younger patients where the evidence base is still evolving, to effectively balance the benefits of treatment against the potential harms. The debates around the use of medicinal cannabis products are highly emotive with strong community opinions and expectations, as well as a notable placebo effect. Additionally, the cost of cannabidiol products is high, and unnecessary use has financial implications for both patients' families and the community. Developing ways to assess individual response to cannabidiol before committing to long‐term use is therefore essential.

To support evidence‐informed decisions regarding this emerging treatment, the Victorian Government's Department of Health and Human Services in Australia, with input from the government's Independent Medical Advisory Committee for Medicinal Cannabis, clinical pharmacologists, neurologists, statisticians, ethicists, pharmacists and a contract research organisation, developed a suite of clinical and pharmacy resources, termed a ‘single patient therapy plan’ (or SPTP) to assist clinicians, patients and their carers determine whether cannabidiol is effective in reducing seizure frequency in an individual patient with severe epilepsy.

The SPTP is based on an N‐of‐1 assessment of treatment efficacy concept,^5^ and the methods and ethical considerations are detailed in this paper. Ethical considerations include that the SPTP is considered clinical practice rather than research, and therefore Human Research Ethics Committee (HREC) approval has not been sought for this treatment methodology. The use of the term SPTP has been purposefully constructed to differentiate its intended use in clinical practice, from the use of N‐of‐1 trials for the primary purpose of population‐based research.

This paper also discusses how the SPTP methodology could potentially be modified for use in other settings with different patient cohorts, conditions and treatments.

Before using the SPTP, treating clinicians should be familiar with its rationale and trained in its processes and have supportive health service infrastructure, including clinic staff and pharmacists. The SPTP is currently available for use at three tertiary hospital sites in Victoria, Australia, for patients under the care of clinicians involved with its development.

## Methods

### Design

The SPTP is designed as a randomised, double‐blind, placebo‐controlled N‐of‐1 trial, for use in a neurology outpatient setting. The primary objective of the SPTP is to assess the efficacy of cannabidiol in reducing seizure frequency in an individual patient with intractable epilepsy, with change in seizure frequency being the primary outcome of interest. The SPTP was made available for use in selected tertiary hospital settings in August 2018, and its use remains ongoing.

**Fig 1:**
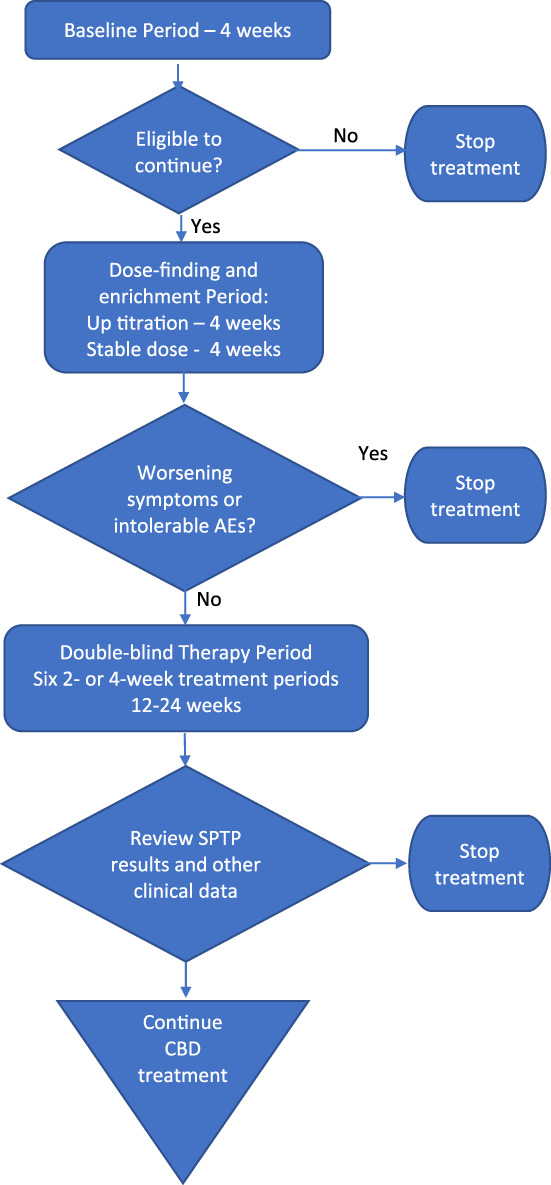
Single patient therapy plan flowchart.

Patients on stable anti‐epileptic medications are initially monitored for 4 weeks to determine their baseline seizure frequency (Fig. 1). Following this there is an open‐label dose‐finding and enrichment period consisting of 4 weeks up‐titration to the maximum tolerated dose and an additional four‐week stable treatment period at that dose (8 weeks in total). Patients able to tolerate treatment then enter a double‐blind therapy cycle of six treatment periods (three active and three placebo treatment periods) in a randomised, counterbalanced design. Treatment periods are of either 2‐ or 4‐weeks duration, depending upon baseline seizure frequency. Patients transition from treatment in one treatment period directly to the next, but for analytic purposes the first 4 days of each treatment period are considered a washout period, based on the anticipated plasma half‐life of cannabidiol in this population, to reduce the impact of treatment in one period impacting on the seizure count in the following treatment period. The statistical model used for assessment of the treatment effect (detailed later) assumes at least 10 expected seizures per analysis period (treatment period minus washout period). Following completion of the six treatment periods, a statistical analysis is performed which provides the probability that treatment has been successful at various thresholds and uses pre‐determined criteria to guide a decision as to whether ongoing treatment is warranted. This then informs discussions about ongoing treatment options between the clinician, patient and their caregivers.

The current recommendations are that cannabidiol is used as an add‐on therapy to existing treatments, and therefore, it is intended that the use of other AEDs remains stable throughout the SPTP process. As cannabidiol has a long half‐life,^6^ it was considered by the advisory group that up‐ and down‐titration of the SPTP products at the beginning and end of each treatment period would not be necessary, although this could be accommodated in the design if required.

Further details about the SPTP are contained within the clinician's module (Appendix S1).

### Participants

The objective of the SPTP is to optimise individual patient treatment outcomes in a patient‐centred approach. Therefore, the decision to use the SPTP occurs after an open dialogue between the clinician and the patient or their carer and requires motivation from the patient and their family to proceed in the interests of determining statistically whether treatment is effective in their individual circumstance. A plain language summary and consent form have been developed (Appendix S2).

Patients can be drawn from neurology outpatient settings, and the SPTP can be considered suitable for use if the patient:

- Is aged greater than 12 months and less than 18 years;
- Has a diagnosis of intractable epilepsy (in the case of the SPTP this is defined as a failure of at least four anti‐epileptic therapies including ketogenic diet and vagal nerve stimulation);
- Experiences at least 10 expected countable seizures per treatment analysis period; and
- Is stable on other AEDs and treatments.

Patients are not suitable for the SPTP if they:

- Have allergy or sensitivity to cannabidiol or product excipients;
- Are female of child‐bearing potential who are currently pregnant or breastfeeding, or planning to become pregnant throughout the duration of the SPTP or within 3 months of completing treatment;
- Have significant heart disease;
- Are assessed as unwilling or unable to comply with the SPTP schedule or assessments.

Treatment with cannabidiol or placebo is in addition to other AEDs and treatments. Prior to starting the SPTP, the patient's medications should be reviewed for possible drug interactions. Due to the known interaction between cannabidiol and clobazam,^7^ consideration may be given to changing clobazam to clonazepam before commencing treatment with cannabidiol.

### Intervention and blinding

The SPTP has been designed to be used with an oral liquid cannabidiol formulation with a matching placebo. The placebo is identical to the active formulation apart from the active ingredient, with appropriate masking agents and identical packaging.

Patients, carers, the clinician and clinic staff are blinded to the sequence allocation.

Packaging and dispensing of the cannabidiol and placebo occur by an unblinded pharmacist familiar with clinical assessment of treatment efficacy procedures, as detailed in the pharmacy module (Appendix S3). The pharmacist remains available for unblinding at the discretion of the treating clinician.

### Assignment of interventions

**Fig 2:**
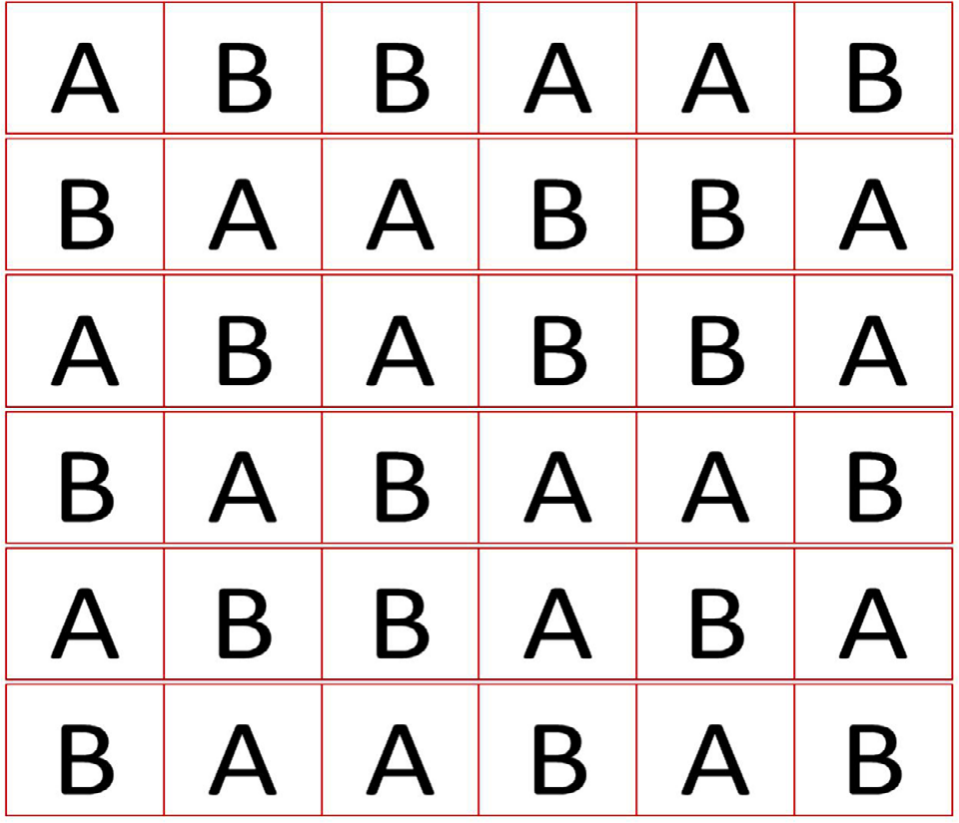
Allocation sequences used (randomly chosen for each patient).

Each patient is allocated to a randomised treatment schedule of six treatment periods, with the sequence of active and placebo periods generated by an independent statistician at a statistical centre (Fig. 2). The unblinded pharmacist can ensure that the correct treatment is dispensed for each of the six treatment periods. A further two treatment periods can be made available if data from one of the treatment periods are unreliable (e.g. due to a hospitalisation or interruption to treatment).

### Monitoring and adherence to treatment

At several points during the SPTP, there are checkpoints at which ongoing participation is evaluated. These include:

- Baseline period – is the patient suitable for an assessment of treatment efficacy?
- On completion of the dose‐finding and enrichment period – was it clear that the patient received no benefit, or was unable to tolerate the dose form/regimen, or did the patient experience undesirable side effects?
- On completion of the assessment of treatment efficacy – determine whether the treatment had an impact on the seizure count.

The SPTP outlines the patient visits required. In addition to an appointment for the initial discussion about the SPTP, and the final discussion where results are provided, patients will need the support of the treating clinician and pharmacists during the dose‐finding and enrichment period when patients are finding a suitable dose for testing, and through the double‐blind therapy cycle.

The patient diary provides a means for verification of patient adherence, as does the return of medication containers to pharmacy between each treatment period.

## Outcomes

The primary outcome is self‐ or carer‐reported seizure counts. Before commencing on the SPTP, the clinician and patient or carer agree to the seizure types to be counted, which may include either all countable seizure types or a subset of countable seizures that are considered more severe or clinically significant. The seizure count period needs to be clearly defined.

Seizure numbers are collected in patient seizure diaries (Appendix S4–S6). On completion of the treatment periods, seizure counts for each treatment period are transferred via a secure website to the statistical centre for analysis.

No secondary outcomes are considered as part of the SPTP due to the complexity of statistical monitoring. Qualitative measures such as duration and severity of seizures and quality of life may be considered informally in addition to the SPTP results when assessing the effectiveness of treatment for an individual patient.

### Safety

Safety monitoring occurs in line with standard medication safety monitoring and regulatory authority reporting processes. Should a serious adverse drug reaction occur, unblinding may be required for treatment and reporting purposes.

Adverse effects known to occur with cannabidiol in the target patient population tend to be mild to moderate in severity, and include gastrointestinal disturbance (diarrhoea, vomiting), fatigue, pyrexia, decreased appetite, convulsions, lethargy and somnolence.^3^, ^4^, ^8^ Less common adverse events include elevated levels of liver aminotransaminase enzymes (generally in patients concurrently taking valproate)^3^, ^4^; therefore, liver function monitoring is warranted.

### Statistical methods

For each patient entered into the SPTP, an analysis is performed of the seizure counts reported in the blinded sequence of active and placebo treatment periods, by a biostatistician implementing a pre‐specified analysis plan (in an independent expert biostatistical centre). This analysis adopts a Bayesian approach, which provides results in the form of posterior probabilities that various levels of benefit (based on the primary outcome measure, seizure frequency) have been achieved under active treatment compared to placebo, accompanied by decision rules that provide thresholds for deciding whether treatment has been successful in the individual patient.

The statistical analysis is based on a model that assumes that seizures occur for each patient according to a Poisson distribution, at a rate that is specific to the patient, but potentially different under active versus placebo medication. The Bayesian analysis provides posterior probabilities for the relative risk reduction, that is the relative reduction in seizure rate seen in the patient, based on the data observed combined with assumptions about the population distribution of seizure counts and patient variation in response to medication. A suggested interpretation of these posterior probabilities, in the form of specific decision rules, is based on the results of simulation experiments that examined the extent to which potential rules would be successful in distinguishing patients that truly benefit (by a pre‐specified amount) from those who do not. Details of the statistical modelling and simulation experiments are outlined in Box 1, with further details provided in Appendix S7.

### Discussion and Conclusion

The SPTP has been designed to accommodate the pragmatic every‐day ethical decision‐making of clinicians caring for individual patients, to assess treatment efficacy for an individual pateint. As an option for clinical management of an individual patient, the SPTP is much more flexible than a clinical trial, as it allows physicians and parents to make decisions in response to the individual child's needs, rather than be bound to a protocol aimed at producing robust research data. For tis reason, a different term (SPTP) has been used to describe this clinical treatment plan rather than an ‘N‐of‐1 trial’, in order to clearly distinguish between research and clinical contexts. In practice, the SPTP and N‐of‐1 trial share common methodology.

Box 1 **Details of statistical model, Bayesian probability calculations and decision rules.**

**Table:** 

The statistical analysis assumes that the number of seizures that occur in any one treatment period (of fixed length, either 2 weeks or 4 weeks) while on active treatment or placebo follows a Poisson distribution. We focus on the ratio of the two seizure rates, which may be interpreted as the relative risk reduction, *R*, for which a value of 1 indicates equivalence in seizure rates between active and placebo. Note that *R* should be thought of as the *true* value that could hypothetically be calculated if we could observe the patient indefinitely under both active and placebo treatments. A key question in concluding as to whether cannabidiol should be continued in a patient is the threshold value of *R* below which treatment would be recommended. This is likely to be a value somewhat below 1, because of the cost and potential side effects of the medication; we focused on the values *R* = 0.8 (20% reduction in seizure rate) and *R* = 0.5 (50% reduction in seizure rate). Our Bayesian analysis focuses on posterior probabilities of the form Pr(*R* < *t*|data), with particular interest in the threshold values mentioned above, i.e. *t* = 0.8 (at least 20% reduction in seizure rate) and *t* = 0.5 (at least 50% reduction in seizure rate). As an example of the interpretation of these calculations, a clinician who believes that treatment should continue if there is good evidence of at least a 20% reduction in seizure rate might decide to recommend treatment if Pr(*R* < 0.8 |data) is high, say at least 0.8 or 80%. To provide guidelines that minimised the need for subjective interpretation of these posterior probabilities, simulation experiments were performed to assess the population performance of a range of specific decision criteria, under a range of assumptions about the population (prior) distributions of the key parameters. The behaviour of the decision rules was characterised in terms of sensitivity and specificity, which represented, respectively, the probability that a patient who should be treated (true *R* below 0.8 or 0.5) will be classified as demonstrating sufficient benefit to meet the recommended threshold, and the probability that a patient who should not be treated will not meet the threshold for recommending treatment. Both the calculations of posterior probabilities and the population behaviour of possible decision rules depend on the statistical model adopted, in particular the prior distribution for *R*. We focused on two scenarios, a relatively ‘optimistic’ one under which it was assumed that half the patients in the target population would obtain at least a 50% reduction in seizure rates (*R* < 0.5), and a more ‘pessimistic’ scenario under which half the patients would obtain at least a 20% reduction in seizure rates (*R* < 0.8). Further details of the simulations and their results are given in Appendix S7. The results led to a decision to provide three posterior probabilities in a report to the treating clinician, along with two recommendations, one for the clinician who would treat at a threshold of (at least) 20% reduction in seizure numbers and the other for the clinician who would only treat at a threshold of 50% reduction. An example of the reporting format is provided in Appendix S8.

### Ethical considerations

The SPTP was considered in the context of an assessment of efficacy of treatment for use in clinical decision‐making by the ethics subcommittee of the Independent Medical Advisory Committee on Medicinal Cannabis, and consultation was held with the HRECs and clinical ethics committees at the three participating tertiary hospitals. It was agreed that the SPTP arrangement is considered part of clinical practice rather than research and HREC approval was not required.

Informed consent remains of utmost importance, including explicit understanding that participation is voluntary and that consent to participate can be withdrawn at any stage of the protocol. This may include where there is no apparent response to treatment, or conversely in the case of a dramatic clear response during the dose finding and enrichment period.

### Dissemination and use in other settings

These SPTP resources have been made available so that they may inform clinical practice in the treatment of severe epilepsy, or be adapted for use in other conditions.

The SPTP is based on N‐of‐1 assessment of treatment efficacy, which are a formalised version of ‘trials of therapy’ that occur every day in clinical practice. It draws on the elements that provide rigour to randomised controlled trials, such as randomisation, blinding, standardised reporting of outcomes and statistical analysis. The aim is to reduce the impact of placebo effects and clinician and patient expectations in ascertaining treatment effectiveness.^9^ Therefore, use of the SPTP can draw on the benefits of a scientific and rigorous approach, and yet have results that inform treatment at an individual patient level.

It is only possible to use an N‐of‐1 approach such as the SPTP in settings where blinded cross‐over of treatment and placebo can be performed and treatment effects are transitory, but in such contexts, it provides a unique opportunity to learn about the individual treatment effect. This includes for novel therapies where the evidence base is evolving, when therapies are proposed for ongoing use, where there is an unavoidable non‐homogeneity in the study group such as diverse causes of the same phenotype (such as epilepsy in this instance), and in patients with multiple comorbidities and concurrent therapies who are often excluded from clinical trials.^10^ For many commonly used medications the majority of patients do not benefit,^11^ and therefore the N‐of‐1 approach can also help establish patients for whom treatment is ineffective so that efforts and resources can be directed elsewhere.

Despite the potential of assessment of treatment efficacy approaches to improve clinical decision‐making and improve patient outcomes by discontinuing ineffective treatments, uptake of the approach has not been strong. This has been attributed in part to practical barriers to use in clinical practice, including obtaining user‐friendly guidance, measuring outcomes, obtaining matching active and placebo formulations and analysing the results.^9^ These SPTP resources are being made publicly available in the hope that they can assist to overcome some of these obstacles, by detailing a model that may be followed and providing guidance documents, consent forms and patient outcome diaries that can be adapted for use as required.

Prior to implementation, the SPTP requires the treating clinician to be familiar with its rationale and trained in its processes and to have supportive health service infrastructure including clinic staff, pharmacist, statistician and hospital clinical ethics committees. The treating clinician will guide the development of the treatment plan, define the patient population, outline safety monitoring requirements, advise on dosing regimens and how the treatment effect will be measured.

The statistician will advise on the statistical issues that need to be considered, such as randomisation and analysis of outcome measures. For each different condition and target patient group, a separate statistical analysis will be required, and a statistical centre must be available to provide a randomised treatment sequence for each patient and to analyse individual patient results promptly to inform clinical practice.

The support of a pharmacy experienced in clinical assessment of treatment efficacy is important. Obtaining a matching placebo formulation to the active drug is not an insurmountable challenge, which could be explored with pharmaceutical sponsors or pharmacies with suitable expertise. The pharmacy also provides the mechanism for unblinding patients if required.

Finally, we recommend that relevant HRECs and Clinical Ethics Committees at any participating clinical sites are consulted prior to implementation of a project based on the SPTP assessment of efficacy methodology, to ensure appropriate oversight. Use of an N‐of‐1 concept such as the SPTP is not common, but in our experience once the concepts are made explicit the model is well supported from an ethical standpoint.

## Supporting information


**Appendix** **S1**. Clinicians module
**Appendix S2**. Single patient therapy plan consent form
**Appendix S3**. Single patient therapy plan pharmacy module
**Appendix S4**. Patient seizure diary baseline, dose finding, maintenance
**Appendix S5**. Patient seizure diary alternating therapy period (2‐week treatment period)
**Appendix S6**. Patient seizure diary alternating therapy period (4‐week treatment period)
**Appendix S7**. Statistical model and simulation evaluation
**Appendix S8**. Example results reporting format
