# Supplementary material for: Protocol for a single patient therapy plan: A randomised, double‐blind, placebo‐controlled N‐of‐1 trial to assess the efficacy of cannabidiol in patients with intractable epilepsy
Source: J Paediatr Child Health. 2020 Sep 23;56(12):1918–23. doi: 10.1111/jpc.15078 (PMC7820972; doi:10.1111/jpc.15078)
Supplement: Supplementary file 1 — Appendix S1. Clinicians module Appendix S2. Single patient therapy plan consent form Appendix S3. Single patient therapy plan pharmacy module Appendix S4. Patient seizure diary baseline, dose finding, maintenance Appendix S5. Patient seizure diary alternating therapy period (2‐week treatment period) Appendix S6. Patient seizure diary alternating therapy period (4‐week treatment period) Appendix S7. Statistical model and simulation evaluation Appendix S8. Example results reporting format [file JPC-56-1918-s001.docx]

**Appendix 1. Clinicians Module**

**Disclaimer:**

These materials are provided for information and education purposes only. Information about a therapy, service, product or treatment does not in any way endorse or support such therapy, service, product or treatment, whether for any individual patient, generally or otherwise.

These materials were developed as a general informational tool to support clinical practice by qualified medical professionals considering medicinal cannabis treatment in patients with epilepsy. They are not a substitute for comprehensive individual clinical assessments and are not intended to constitute a comprehensive guide for assessing whether treatment with medicinal cannabis is or will be beneficial for any individual patient or any particular class of patients, or regarding any other aspect of treatment of patients with medicinal cannabis.

Decisions regarding treatment with medicinal cannabis, and any determination regarding whether treatment with medicinal cannabis is suitable in the circumstances, are the sole responsibility of the treating medical professional, exercising their own clinical judgment and taking into account all of the circumstances and medical history of the patient/s. For the avoidance of doubt, these materials were not developed for use by patients, and patients are solely responsible for seeking medical advice in relation to any treatment with medicinal cannabis.

The State of Victoria and the Department of Health and Human Services shall not bear any liability to you or any third party for any loss or damage which may result from your use of or reliance on any information contained in these materials, including but not limited to the use or application of any information contained in these materials, generation of data by these materials, analysis of the data generated by these materials, and/or any determinations made regarding treatment with medicinal cannabis for an individual patient, a class of patients or generally.

**SINGLE PATIENT THERAPY PLAN (N-of-1) FOR USE OF CANNABIDIOL IN PAEDIATRIC EPILEPSY**

TREATING CLINICIAN MODULE

This Single Patient Therapy Plan (N-of-1) has been prepared to support the Treating Clinician and patient assess whether cannabidiol is of benefit as a treatment for severe intractable epilepsy in that individual patient. The rationale is to ensure that this vulnerable patient group has access to medicinal cannabis supported by a treatment plan for decision making, in the context of an emerging evidence base.

The framework provided in the treatment plan will help in making decisions about whether a patient is benefitting from medicinal cannabis. The Single Patient Therapy Plan requires certain restrictions for patient eligibility for safety and statistical purposes. Use of this plan is at the discretion of the Treating Clinician, in conjunction with the patient and/or their caregiver.

Prior to using this Single Patient Therapy Plan, the Treating Clinician should be sufficiently trained in its use and have the support of the treating health service. The Single Patient Therapy Plan will be discussed with hospital executives and relevant committees prior to implementation.

1. SYNOPSIS

| **Title** | Single Patient Therapy Plan (N-of-1) for use of cannabidiol in children with severe epilepsy |
| --- | --- |
| **Indication:** | Severe Intractable Epilepsy |
| **Products:** | Active: Cannabidiol 100 mg/mL oil formulation for oral administration. During the enrichment period, patients will receive cannabidiol as a twice daily (bd) dose, starting with a dose of 5 mg/kg/day (2.5 mg/kg twice daily) for the first week. If this is tolerated the dose will increase each week to 10 mg/kg/day (5 mg/kg twice daily), 15 mg/kg/day (7.5 mg/kg twice daily) and 20 mg/kg/day (10 mg/kg twice daily), or until undesirable side effects are observed (maximum dose 1000 mg/day). To complete the enrichment period, the patient will be dosed with the maximum dose established for them (selected dose), for 4 weeks.  Placebo: Oil formulation without cannabidiol for oral administration.  During the alternating therapy period, patients will receive cannabidiol oil or placebo oil bd at the established dose, according to the randomization schedule. |
| **Duration:** | Baseline Period: 4 weeks  Enrichment period: maximum 8 weeks (up to 4 weeks up titration/dose-finding and 4 weeks treatment on selected dose).  Alternating therapy period:   \| 6 treatment periods of 2 weeks duration (total 12 weeks).* \| **OR** \| 6 treatment periods of 4 weeks duration (total 24 weeks).* \| \| --- \| --- \| --- \| \| Each treatment period comprises 4 days washout period and 10 days analysis period. \| Each treatment period comprises 4 days washout period and 24 days analysis period. \|   **Note an additional 2 treatment periods are available at the Treating Clinician’s discretion, should they believe that there was a period with unreliable data.*  Follow-Up Period: 2 weeks  The anticipated duration for a patient would therefore be approximately 6 to 9 months depending on the length of the treatment periods. |
| **Objectives of the Single Patient Therapy Plan:** | The primary purpose of the Single Patient Therapy Plan is to assist with individual treatment decisions. To support this the following measures will be reviewed:  The frequency of seizures during the Single Patient Therapy Plan  Occurrence of side effects during the Single Patient Therapy Plan |
| **Design:** | Single Patient Therapy Plan for single-patient use.  Enrichment period: Open-label, dose-finding.  Alternating therapy period: Double-blind, randomized, placebo-controlled, repeated-crossover (6 treatment periods in a counterbalanced design). |
| **Eligibility Criteria (Inclusion and Exclusion)** | **Inclusion Criteria:**   1. Children aged greater than 12 months to less than 18 years at time of starting enrichment period. 2. Prior diagnosis of intractable epilepsy, defined as failure of 4 antiepileptic drugs (AEDs). *Note: ketogenic diet and vagal nerve stimulation are included as an AED.* 3. At least 10 expected countable seizures per treatment analysis period.  \| **Baseline Seizure Count** \| **Treatment Period Duration** \| \| --- \| --- \| \| ≥ 10 seizures in 10 days \| 2 weeks \| \| ≥ 10 seizures in 24 days, but <10 in 10 days \| 4 weeks \| \| < 10 seizures in 24 days \| Patient not suitable for this Single Patient Therapy Plan \|   *Note: The seizures to be included in the seizure count will be determined on a patient by patient basis. Seizure recording/reporting windows will also be determined on a patient by patient basis.*  **Exclusion Criteria:**   1. Sensitivity or allergy to Cannabidiol or its excipients. 2. Females of child bearing potential who are currently pregnant or breastfeeding, or planning on becoming pregnant during the duration of the Single Patient Therapy Plan or within 3 months of treatment completion. 3. Presence of significant medical illness such as heart disease. 4. In the opinion of the Treating Clinician, the patient/parent(s)/caregiver(s) are unable to comply with the Single Patient Therapy Plan schedule or assessments.   Concomitant medications are to be reviewed by the Treating Clinician and considered for possible drug-drug interactions with Cannabidiol. The Treating Clinician might consider changing medication from clobazam to clonazepam, prior to commencing the Single Patient Therapy Plan.  **Considerations for Withdrawal from Treatment:**  Patients with alanine aminotransferase (ALT) and aspartate aminotransferase (AST) levels ≥ 3 x upper limit of normal (ULN) should be reviewed and considered for withdrawal.  Any other significant Adverse Drug Reactions (ADRs), inability to comply with the Single Patient Therapy Plan, at request of patient or carer, or at discretion of Treating Clinician. |
| **Procedures:** | **Baseline period:**  Patients will be asked to record baseline seizure data for a period of 4 weeks. On completion of the baseline period, patients will be reviewed by the Treating Clinician. Those who meet the eligibility criteria will continue to the enrichment treatment period, patients who do not meet the eligibility criteria will leave the Single Patient Therapy Plan at this point.  **Enrichment treatment period:**  Patients will commence an up-titration period (4 weeks). The starting dose is 5 mg/kg/day (2.5 mg/kg twice daily) cannabidiol, the dose will increase each week to 10 mg/kg/day (5 mg/kg twice daily), 15 mg/kg/day (7.5 mg/kg twice daily) and 20 mg/kg/day (10 mg/kg twice daily), or until undesirable side effects are observed (maximum dose 1000 mg/day). Once undesirable side effects are observed, the Treating Clinician may drop back to the previous dose and proceed to 4 weeks of treatment at the selected dose.  Once the maximum dose is established for the patient, the patient will remain on this dose for 4 weeks.  Enrichment period review. The Treating Clinician and patient will decide if the patient should continue onto the alternating therapy period based on perceived effects/deteriorations and side effects observed. Those who experience a worsening of seizures, or patients with unacceptable side effects or who do not believe there has been any benefit on treatment will leave the Single Patient Therapy Plan at this point. The rest will continue to the alternating therapy period.  **Alternating therapy period:**  Patients will be randomized to one of 6 treatment sequences; each sequence will outline the treatment schedule for 6 treatment periods. Treatment periods will be set as either 2 or 4 weeks by the Treating Clinician, depending on the number of baseline seizures recorded. The randomization is counterbalanced (3 active and 3 placebo). On completion of the alternating therapy period, results will be analysed and prepared for review by the statistical group for the Treating Clinician with the patient. The patient will have completed the Single Patient Therapy Plan at this point.  During the alternating therapy period, there will be no washout period in which treatment is withheld between treatment periods; patients will go straight from one treatment to the next. The analysis plan will allow for carryover effects by omitting the first 4 days of each treatment period from the analysis, thus effectively considering this as a washout period. The remaining days of each treatment period will be used for analysis. Therefore, the analysis period will be either 10 or 24 days in duration (for 2 and 4 week treatment periods respectively).  If it is decided that Cannabidiol was effective in reducing seizure numbers, with an acceptable safety profile, patients may continue taking cannabidiol. For those whose treatment was not successful, the Treating Clinician will discuss alternative treatment options.  **Follow-up Period:**  Patients will return to the clinic for a follow-up visit 14 days following completion of the alternating therapy period. If ongoing ADRs exist, follow-up will extend beyond this as needed. |
| **Assessments** | **Safety**  Safety assessments include the frequency and type of ADRs. ADRs may be reported by the patient/caregiver or may be identified through routine investigations at the discretion of the Treating Clinician.  Safety bloods are to be collected and reviewed at baseline, end of enrichment period, mid-way through the alternating therapy period, at the end of the Single Patient Therapy Plan, and as required per routine clinical practice.  **Efficacy**  Efficacy assessments are based on the seizure counts. On completion of the 6 treatment periods, the Treating Clinician is to assess if there were any treatment periods with corrupt or unreliable data. A further 2 treatment periods are permitted in such instance. Seizure frequency data will be sent for statistical analysis and the success of the cannabidiol treatment in individual patients will be statistically analysed for each patient. |

1. TABLE OF CONTENTS

[TREATING CLINICIAN MODULE 1](#_Toc522180986)

[1.0 SYNOPSIS 2](#_Toc522180987)

[2.0 TABLE OF CONTENTS 6](#_Toc522180988)

[3.0 GLOSSARY OF TERMS AND ABBREVIATIONS 6](#_Toc522180989)

[4.0 BACKGROUND 7](#_Toc522180990)

[5.0 GOALS 8](#_Toc522180991)

[6.0 PATIENT ELIGIBILITY 9](#_Toc522180992)

[7.0 TREATMENT 10](#_Toc522180993)

[8.0 SEIZURE RECORDING 11](#_Toc522180994)

[9.0 SAFETY MONITORING 12](#_Toc522180995)

[10.0 TREATMENT PLAN FLOW CHART 16](#_Toc522180996)

[11.0 ETHICAL FRAMEWORK 17](#_Toc522180997)

1. GLOSSARY OF TERMS AND ABBREVIATIONS

| ADR | Adverse Drug Reaction |
| --- | --- |
| AED | Antiepileptic Drug (or medication) |
| ALT | Alanine aminotransferase |
| ARTG | Australian Register of Therapeutic Goods |
| AST | Aspartate aminotransferase |
| bd | Twice daily |
| SADR | Serious Adverse Drug Reaction |
| TGA | Therapeutic Goods Administration |
| ULN | Upper limit of normal |

1. BACKGROUND

Epilepsy is a term used to describe recurrent seizures in patients. These may originate from a number of disorders including genetic syndromes, strokes, infections and traumatic brain injuries. With treatment, it is estimated that 60-70% of patients with epilepsy are seizure-free, however, approximately one third of patients fail to find an approved antiepileptic medication (AED) to control their seizure activity with acceptable side effects. When seizures continue despite medication, other treatment options can include surgery, stimulation of the vagus nerve, ketogenic diet or complementary medicines. The consequences of recurrent seizures include damage to the brain and adverse impacts on quality of life.

Evidence from laboratory studies, anecdotal reports, and small clinical studies suggest that cannabidiol, a non-psychoactive compound of cannabis, could potentially be helpful in controlling seizures. The legalities of medicinal cannabis use are being reviewed by many countries, and clinical research is on-going.

- 1. Victorian Setting

The Commonwealth Department of Health regulates medicinal cannabis products through the Therapeutic Goods Administration (TGA) and the Office of Drug Control. The TGA administers the legislation which establishes the regulatory framework for all medicines in Australia. Currently most medicinal cannabis products have not undergone the requisite evaluation of efficacy and safety to be registered on the Australian Register of Therapeutic Goods (ARTG) and are therefore considered to be unapproved therapeutic goods. The following routes are available to enable access to unapproved medicinal cannabis products in Australia:

- Access for individual patient through Authorised Prescriber Scheme
- Access for individual patient through Special Access Scheme
- Access as part of a clinical trial

More information about accessing unapproved products is available on the TGA website^[[1]](#footnote-1)^.

The Office of Medicinal Cannabis was established within the Department of Health and Human Services for the Victorian State Government and is responsible for establishing the Victorian medicinal cannabis framework. This includes administration of the Victorian *Drugs, Poisons and Controlled Substances Act 1981*, under which cannabis derived products are either classified as Schedule 8 (products with more than 2% Δ9-tetrahydro-cannabinol) or Schedule 4 (products with less than 2% Δ9- tetrahydro-cannabinol).

- 1. Rationale for this Single Patient Therapy Plan

As a Schedule 4 drug, supply of cannabidiol will need to be prescribed by a medical practitioner as part of an overall treatment plan.

A Single Patient Therapy Plan (or N-of-1 trial) is an evidence-based model used for making individual patient treatment decisions^[[2]](#footnote-2)^. This model ensures that each patient receives medical care tailored to their individual needs.

The Single Patient Therapy Plan initially aims to find a safe and efficacious dose for each patient, and to identify patients who are unable to tolerate cannabidiol in an enrichment period. Patients whose epilepsy worsens, or who are unable to tolerate the cannabidiol will not proceed beyond the enrichment period. For the patients who do tolerate cannabidiol during the enrichment period, cannabidiol will be tested in a highly sensitive, repeated-crossover, placebo-controlled design to determine whether treatment can be considered successful or un-successful in each individual patient.

Prior to using this Single Patient Therapy Plan, clinicians should ensure they have read and understand the plan and are familiar with the process and its implications.

1. GOALS

The primary purpose of the Single Patient Therapy Plan is to support the patient and the Treating Clinician in making individual treatment decisions. To support this the following measures will be reviewed:

- The frequency of seizures during the Single Patient Therapy Plan.
- Occurrence of side effects during the Single Patient Therapy Plan.

As medicinal cannabis is not an approved therapeutic product, this Single Patient Therapy Plan includes several safety check points, to ensure the safety and wellbeing of the patient:

- Baseline period: Patients will be reviewed for suitability to participate in this Single Patient Therapy Plan.
- Enrichment period: Patients will complete an 8-week enrichment period which includes up to 4 weeks of dose finding and 4 weeks of treatment at the selected dose. On completion of the enrichment period, the patient will be reviewed to determine if it is suitable for them to continue to the alternating therapy period. This includes review of the patient diary for seizure counts and any safety concerns. Patients with perceived benefit and who have tolerated cannabidiol in the enrichment period will proceed to the alternating therapy period.
- Alternating therapy period: This phase of the Single Patient Therapy Plan is randomized, double-blind, and placebo controlled. Patients will complete 6 treatment periods (of either 2 weeks or 4 weeks duration). They will receive placebo for 3 treatment periods and cannabidiol for 3 treatment periods, neither the patient nor the Treating Clinician will know what treatment the patient is receiving. The patient may be withdrawn from the Single Patient Therapy Plan during the Alternating therapy period if the Treating Clinician is concerned about side effects.

1. PATIENT ELIGIBILITY
   1. Inclusion Criteria

Patients are eligible to use the Single Patient Therapy plan if they are:

1. Aged greater than 12 months to less than 18 years at time of starting enrichment period.
2. Have a diagnosis of intractable epilepsy, defined as failure of 4 AEDs. *Note: ketogenic diet and vagal nerve stimulation are included as an AED.*
3. Experiences at least 10 expected countable seizures per treatment analysis period.

| **Baseline Seizure Count** | **Treatment Period Duration** |
| --- | --- |
| ≥ 10 seizures in 10 days | 2 weeks |
| ≥ 10 seizures in 24 days, but < 10 in 10 days | 4 weeks |
| < 10 seizures in 24 days | Patient not suitable for this Single Patient Therapy Plan |

*Note: The seizures to be included in the seizure count will be determined on a patient by patient basis (Refer to Section 8 - Seizure recording). Seizure recording/reporting windows will also be determined on a patient by patient basis.*

- 1. Exclusion Criteria

1. Sensitivity or allergy to cannabidiol or its excipients.
2. Patients (female) of child bearing potential who are currently pregnant or breastfeeding or planning on becoming pregnant during the duration of the Single Patient Therapy Plan or within 3 months of completing treatment.
3. Presence of significant medical illness such as heart disease.
4. In the opinion of the Treating Clinician, the patient/parent(s)/caregiver(s) are unable to comply with the Single Patient Therapy Plan schedule or assessments.
   1. Consideration for Withdrawal from Treatment

Patients with alanine aminotransferase (ALT) and aspartate aminotransferase (AST) levels ≥ 3 x upper limit of normal (ULN) should be reviewed and considered for withdrawal.

Patients with any other significant adverse drug reactions (ADRs), or inability to comply with Single Patient Therapy Plan, should be considered for withdrawal at request of patient or carer, or at discretion of Treating Clinician.

Patients experiencing unexpected status epileptics should be unblinded, reviewed and ongoing medical care provided at the discretion of the Treating clinician.

1. TREATMENT
   1. Product

This Single Patient Therapy Plan has been developed for use with a purified cannabidiol formation in oil for oral administration, suitable for use in a paediatric patient population, and a matching placebo oil.

The design of the Single Patient Therapy Plan includes a randomised phase where patients switch between active and placebo for six to eight treatment periods. As cannabidiol has a long half-life, and patients will remain on stable AEDs throughout, there will be no up- and down-titration of the products. Review of other trials of cannabidiol in a paediatric population has revealed no evidence that this would be harmful^[[3]](#footnote-3)^,^[[4]](#footnote-4)^.

During the enrichment period, patients will receive cannabidiol as a twice daily dose (bd), starting with a dose of 5 mg/kg/day for the first week, if this is tolerated the dose will increase each week to 10, 15 and 20 mg/kg/day, or until undesirable side effects are observed (maximum dose 1000 mg/day). To complete the enrichment period, the patient will be dosed with the maximum dose established for them (selected dose), for 4 weeks.

During the alternating therapy period, patients will receive cannabidiol oil or placebo oil bd at the established dose, according to the randomization schedule. There will be no up- or down-titration of cannabidiol oil or placebo oil during this period, nor will there be any washout period in which the treatment will be withheld between treatment periods, i.e. patients will go straight from one treatment period to the next. Both the patient and the Treating Clinician will be blinded to the treatment allocation.

Should non-serious adverse drug reactions occur, the dose may be decreased during the alternating treatment phases, in line with clinical judgement.

Patients will be required to return to the hospital for a visit at completion of each treatment period. They will be asked to return all previously dispensed bottles. Patients are to be informed that NO bottle can be ‘lost’ and all bottles must be returned to ensure ongoing access. If any bottles are not returned, the patient may return later to collect new supplies, this will not adversely affect the analysis of the results.

- 1. Dietary Restrictions

Patients should take cannabidiol within 30 minutes of consuming a significant portion of food.

- 1. Drug-Drug Interaction

Concomitant medications are to be reviewed by the Treating Clinician and considered for possible drug-drug interactions with cannabidiol. The Treating Clinician might consider changing medication from clobazam to clonazepam, prior to commencing the Single Patient Therapy Plan, due to the known interaction between cannabidiol and clobazam.

Monitoring of blood levels of AED medications will occur in response to ADRs as clinically indicated to monitor for any drug-drug interactions with cannabidiol.

- 1. Known and Potential Risks

There are currently no cannabis products available in Australia that have been approved by the TGA for use in children with severe epilepsy. Nabiximos has TGA approval for the treatment of muscle spasticity in adults with multiple sclerosis. The U.S. Food and Drug Administration has recently approved Epidiolex oral solution for the treatment of seizures associated with the rare and severe forms of epilepsy, Lennox-Gastaut syndrome and Dravet syndrome, in patients two years of age and older.

Based on published Adverse Event data from paediatric patients treated with cannabidiol products for epilepsy^[[5]](#footnote-5),^^[[6]](#footnote-6),^^[[7]](#footnote-7)^, the anticipated risks associated with the cannabidiol may include;

- Gastrointestinal disturbances including Diarrhoea and Vomiting
- Sleep disorder, Drowsiness, Fatigue, Lethargy, Somnolence, Poor quality sleep and Insomnia
- Pyrexia
- Decreased appetite
- Convulsion
- Transaminase elevations
- Malaise, and Asthenia
- Rash
- Infections

Cannabidiol is contraindicated for use in:

- Patients with a known hypersensitivity to cannabidiol
- Women of childbearing potential, who are breastfeeding or pregnant or are intending to become pregnant or men intending to father children
- Patients with heart failure

1. SEIZURE RECORDING

At the start of the baseline period, the Treating Clinician with the parent/caregiver of the patient will define the seizure types to be recorded for the patient, as well as seizure recording and reporting periods to be used for the patient to ensure consistency across the therapy period.

Two options exist for defining seizure types to be included in the seizure counts:

Option 1: Include all types of countable seizures (to total ≥ 10 in a treatment period), i.e. statistical analysis for treatment success will be based on all seizures.

Option 2: Include only a subset of countable seizure types that are considered more severe or clinically significant (to total ≥ 10 in a treatment period) i.e. statistical analysis for treatment success will be based on these more severe/clinically significant seizure types.

***Note that once the seizure types and seizure recording/reporting periods have been defined for the patient they must be documented consistently across the duration of the project.***

Seizures will be recorded in patient diaries, which will be collected and retained in the patient’s source notes.

On completion of the 6 treatment periods in the Alternating therapy period, the Treating Clinician will assess if there was a treatment period with corrupt or unreliable data. Reasons for corrupt or unreliable data may include but are not limited to: unanticipated hospitalisations, use of additional AEDs in that treatment period, breaks from treatment during the treatment period. A further 2 treatment periods are permitted in such instance. Seizure counts from the Alternating therapy period will be sent via a secure web-based reporting system to the statistical centre. In the instance where 8 treatment periods were used, the Treating Clinician will identify the treatment period with unreliable or corrupt data to be excluded from analysis. At the statistical centre, results will be analyzed with results reported in a timely fashion (no more than a week) to the Treating Clinician. Details of the statistical analysis are provided in the Statistical Module.

1. SAFETY MONITORING
   1. Definitions

Adverse drug reaction (ADR) for unapproved medicines: all noxious and unintended responses to a medicinal product related to any dose should be considered adverse drug reactions. The phrase 'responses to a medicinal product' means that a causal relationship between a medicinal product and an ADR is at least a reasonable possibility, i.e. the relationship cannot be ruled out.

Unexpected Adverse Drug Reaction: An adverse reaction, the nature or severity of which is not consistent with the applicable product information.

Serious Adverse Drug Reaction (SADR): Any untoward medical occurrence that at any dose:

- Results in death;
- Is life-threatening (i.e., the ADR, in the view of the Treating Clinician, places the patient at immediate risk of death);
- Requires or prolongs inpatient hospitalization. Hospitalization does not need to be reported as an SADR where the planned hospitalization or prolonged hospitalization was for a diagnostic or elective surgical procedure for a preexisting medical condition that has not changed;
- Results in persistent or significant disability/incapacity (i.e., the ADR results in substantial disruption of the patient’s ability to conduct normal life functions);
- Is a congenital anomaly/birth defect in a neonate/infant born to a female patient or female partner of a male patient exposed to the drug treatment(s);
- Considered a significant drug reaction by the Treating Clinician (e.g., may jeopardize the patient or may require medical/surgical intervention to prevent one of the outcomes listed above).

Note: the term 'life-threatening' in the definition of 'serious' refers to a reaction in which the patient was at risk of death at the time of the event; it does not refer to a reaction which hypothetically might have caused death if it were more severe.

- 1. Safety Recording

All ADRs, whether reported by the patient or noted by the Treating Clinician, will be recorded in the patient’s medical record. For each ADR recorded, the Treating Clinician will make an assessment of seriousness, severity, and causality. Treating Clinicians should use correct medical terminology/concepts when recording ADRs:

Diagnosis versus Signs and Symptoms: If known, a diagnosis should be recorded rather than individual signs and symptoms (e.g., record only liver failure or hepatitis rather than jaundice, asterixis, and elevated transaminases). However, if a constellation of signs and/or symptoms cannot be medically characterized as a single diagnosis or syndrome at the time of reporting, each individual event should be recorded as an ADR. If a diagnosis is subsequently established, it should be reported as follow-up information.

Adverse Drug Reactions Occurring Secondary to Other Events: In general, ADRs occurring secondary to other events (e.g., cascade events or clinical sequelae) should be identified by their primary cause. For example, if severe diarrhea is known to have resulted in dehydration, it is sufficient to record only diarrhea as an ADR. Medically significant ADRs occurring secondary to an initiating event that are separated in time should be recorded as independent events. For example, if a severe gastrointestinal hemorrhage leads to renal failure, both events should be recorded separately.

Abnormal Laboratory Values: Only clinically significant laboratory abnormalities that require active management will be recorded as ADRs (e.g., abnormalities that require drug dose modification, discontinuation of treatment, more frequent follow-up assessments, further diagnostic investigation, etc.). If the clinically significant laboratory abnormality is a sign of a disease or syndrome (e.g., alkaline phosphatase and bilirubin 5 x ULN associated with cholecystitis), only the diagnosis (e.g., cholecystitis) needs to be recorded. If the clinically significant laboratory abnormality is not a sign of a disease or syndrome, the abnormality itself should be recorded as an ADR or SADR. If the laboratory abnormality can be characterized by a precise clinical term, the clinical term should be recorded as the ADR or SADR. For example, an elevated serum potassium level of 7.0 mmol/L should be recorded as “hyperkalemia.” Observations of the same clinically significant laboratory abnormality from visit to visit should not be repeatedly recorded as ADRs, unless their severity, seriousness, or etiology changes.

Preexisting Medical Conditions: A preexisting medical condition is one that is present at the screening/baseline period. A preexisting medical condition should be recorded as an ADR only if the frequency, severity, or character of the condition worsens during the study. When recording such events, it is important to convey the concept that the preexisting condition has changed by including applicable descriptors (e.g., “more frequent headaches”).

- 1. Safety Reporting

The Treating Clinician will report ADRs reported during treatment with cannabidiol to the TGA as follows (*Note: this means that patients on the alternating treatment phase may need to be unblinded to ensure reporting timelines are met*):

- For fatal or life-threatening serious and unexpected ADRs, initial report is to be sent to the TGA within seven calendar days of first knowledge. Follow-up with complete report within eight additional calendar days. For all other serious and unexpected ADRs, full report no later than fifteen calendar days of first knowledge.
- Other ADRs: to be reported on request by the TGA via data tabulations.

Copies of all safety reports sent to the TGA should be sent to the Program Coordinator. Further pharmacovigilance reporting requirements may be supplied.

### Assessment of Seriousness

Refer to the definition in Section 8.1 for SADRs. All ADRs that do not meet any of the criteria for serious should be regarded as non-serious ADRs.

The terms “severe” and “serious” are not synonymous. Severity refers to the intensity of an ADR (as in mild, moderate, or severe pain); the ADR itself may be of relatively minor medical significance (such as severe headache). “Serious” is a regulatory definition and is based on patient or event outcome or action criteria usually associated with events that pose a threat to a patient’s life or vital functions. Seriousness (not severity) serves as the guide for defining regulatory reporting obligations.

If a Treating Clinician’s opinion of relationship of cannabidiol or placebo to an SADR is possibly, unlikely related, or not related to cannabidiol, another cause of event must be provided by the Treating Clinician in the SADR report.

### Assessment of Severity

To ensure consistency of severity assessments, Treating Clinician should apply the following general guidelines:

Mild: Transient or mild discomfort (< 48 hours); no interference with the patient’s daily activities; no medical intervention/therapy required.

Moderate: Mild to moderate interference with the patient’s daily activities; no or minimal medical intervention/therapy required

Severe: Considerable interference with the patient’s daily activities; medical intervention/therapy required; hospitalization possible

### Assessment of Causality

To ensure consistency of causality assessments, Treating Clinician should apply the following general guidelines:

Probably Related: An event has a strong temporal relationship to cannabidiol or placebo, or recurs on re-challenge and another cause of event is unlikely or significantly less likely.

Possibly Related: An event has a strong temporal relationship to cannabidiol or placebo use and another cause of event is equally or less likely compared to the potential relationship to study drug.

Unlikely Related: An event has little or no temporal relationship to cannabidiol or placebo use and/or a more likely other cause of event exists.

Not Related: An event is due to an underlying or concurrent illness or effect of another drug and is not related to cannabidiol or placebo use (e.g. has no temporal relationship to cannabidiol or placebo use or has a much more likely other cause of event).

***Note: events considered Probably Related and Possibly Related are to be reported as ADRs.***

1. TREATMENT PLAN FLOW CHART

| **Baseline Period: 4 weeks (Day 1 to Day 28)** | |
| --- | --- |
| **VISIT 1**  Day 1 | Informed consent (parents)  Inclusion/exclusion criteria review  Safety laboratory parameter review  Define seizure types to be recorded for the patient  Define seizure recording and reporting periods to be used for the patient  Dispense patient diary with instructions |
| **VISIT 2**  Day 28 | Review patient diary |
| *□ Check point – Is this patient eligible to continue to the enrichment period, if not Single Patient Therapy Plan is now complete for this patient.* | |

| **Enrichment Period Up Titration: 4 weeks (Day 1 to Day 28)** | | |
| --- | --- | --- |
| **VISIT 2**  Day 1 | | Record patients weight  Prescribe/dispense cannabidiol and patient diaries  Provide treatment up-titration plan to families. Starting dose 5 mg/kg/day  Provide patient diary and instructions to collect ADRs |
| **PARENTAL INSTRUCTIONS** | Day 8 | If the patient experiences undesirable side effects at the 5 mg/kg/day dose, parents should contact the site for withdrawal from the treatment plan.  If side effects permit, parents are instructed to increase dose to 10 mg/kg/day. |
|  | Day 15 | If the patient experienced undesirable side effects at the 10 mg/kg/day dose, up-titration is complete, parents should contact the site to confirm Selected Dose is 5 mg/kg/day, patient continues taking 5 mg/kg/day until Visit 3.  If side effects permit, parents are instructed to increase dose to 15 mg/kg/day. |
|  | Day 22 | If the patient experienced undesirable side effects at the 15 mg/kg/day dose, up-titration is complete, parents should contact the site to confirm Selected Dose is 10 mg/kg/day, patient continues taking 10 mg/kg/day until Visit 3.  If side effects permit, parents are instructed to increase dose to 20 mg/kg/day. |
| **VISIT 3**  Day 29 | | If the patient experienced undesirable side effects at the 20 mg/kg/day dose, parents should note Selected Dose is 15 mg/kg/day. Up-titration is complete. Review weeks 1 to 4 patient diaries.  Review ADRs and concomitant medications.  Confirm Selected Dose based on the ADRs reported. |

| **Enrichment Period Selected Dose: 4 weeks (Day 1 to Day 28)** | |
| --- | --- |
| **VISIT 3**  Day 1 | Prescribe/dispense selected dose cannabidiol and patient diary |
| **VISIT 4**  Day 28 | Review weekly patient diaries  Review ADRs and concomitant medications  Safety laboratory parameter review |
| *□ Check point – Is there any perceived benefit for the patient, does the patient wish to proceed? Is it safe for the patient to proceed to the alternating therapy period? It is recommended that patients whose epilepsy worsens, or who experience undesirable side effects, do not proceed to the alternating therapy period.* | |

| **Alternating Therapy Period: 6 Treatment Periods** |
| --- |
| **VISIT 4**  Randomise patient (pharmacist)  Provide patient diary cards and instructions to collect ADRs  Treatment Periods are either 2 weeks duration or 4 weeks duration |
| **ADDITIONAL VISITS**  Patients will be seen on completion of each treatment period during the alternating therapy period.  At each visit the Treating Clinician will review ADRs and concomitant medications.  Patients and their caregivers will be required to return patient diaries and previously dispensed bottles of cannabidiol/placebo. Patients are to be informed that NO bottle can be ‘lost’ and all bottles must be returned to ensure ongoing access. If any bottles are not returned, the patient may return later to collect new supplies, this will not adversely affect the analysis of the results.  Mid way through the alternating therapy period, and on completion of the alternating therapy period, Treating Clinicians will review safety laboratory parameters.  Should non-serious adverse drug reactions occur, the dose may be decreased during the alternating treatment phases, in line with clinical judgement.  On completion of 6 treatment periods, the Treating Clinician will determine whether there were any treatment periods with corrupt or unreliable data. A further 2 treatment periods are permitted in such instance. On completion of the alternating therapy period, seizure counts will be submitted for statistical analysis, the Treating Clinician will identify any treatment period with corrupt or unreliable data to be excluded from the analysis. |
| **RESULTS VISIT**  Results Visit at completion of the alternating therapy period. *Note a minimum of 7 days will be required for the statistical results to be available.*   - Review of unblinded patient results (formal seizure frequency assessment, safety) - Review ADRs and concomitant medications. Monitor ADRs to resolution |
| *□ Check point –* *Is this patient suitable for ongoing treatment with cannabidiol with ongoing safety monitoring and reporting* |

Abbreviations: ADR = Adverse Drug Reaction

** Note: other visits may be scheduled per normal care at the Treating Clinicians discretion.*

1. ETHICAL FRAMEWORK
   1. Consent

The Treating Clinician will obtain consent from the parent/legal guardian of each patient to participate in this Single Patient Therapy Plan. Where possible, assent will be obtained from the patient. This consent form will be retained in the patients notes. The prospective consent will include a consent to use de-identified information generated for audit purposes.

- 1. Ethics

The Single Patient Therapy Plan is considered to be part of clinical practice rather than research and should be discussed with relevant clinical ethics committees at health services prior to its use.

Should an audit of the results be required subsequently, human research ethical approval will be sought for the audit procedure.

**Appendix 2. Single Patient Therapy Plan Consent Form**

**Disclaimer:**

These materials are provided for information and education purposes only.

Information about a therapy, service, product or treatment does not in any way endorse or support such therapy, service, product or treatment, whether for any individual patient, generally or otherwise.

These materials were developed as a general informational tool to support clinical practice by qualified medical professionals considering medicinal cannabis treatment in patients with epilepsy. They are not a substitute for comprehensive individual clinical assessments and are not intended to constitute a comprehensive guide for assessing whether treatment with medicinal cannabis is or will be beneficial for any individual patient or any particular class of patients, or regarding any other aspect of treatment of patients with medicinal cannabis.

Decisions regarding treatment with medicinal cannabis, and any determination regarding whether treatment with medicinal cannabis is suitable in the circumstances, are the sole responsibility of the treating medical professional, exercising their own clinical judgment and taking into account all of the circumstances and medical history of the patient/s. For the avoidance of doubt, these materials were not developed for use by patients, and patients are solely responsible for seeking medical advice in relation to any treatment with medicinal cannabis.

The State of Victoria and the Department of Health and Human Services shall not bear any liability to you or any third party for any loss or damage which may result from your use of or reliance on any information contained in these materials, including but not limited to the use or application of any information contained in these materials, generation of data by these materials, analysis of the data generated by these materials, and/or any determinations made regarding treatment with medicinal cannabis for an individual patient, a class of patients or generally.

**SINGLE PATIENT THERAPY PLAN FOR THE USE OF CANNABIDIOL IN PAEDIATRIC INTRACTABLE EPILEPSY**

***PARTICIPANT INFORMATION SHEET AND CONSENT FORM FOR PARENTS AND GUARDIANS***

**Background**

A Single Patient Therapy Plan is a way of giving a treatment, that helps to decide if the treatment is effective or not. In this case, it is to assess whether treatment with medicinal cannabis is effective in the treatment of severe epilepsy. This information sheet explains how the Single Patient Therapy Plan works. It will help you decide if you or your child would like to try treatment with medicinal cannabis using the Single Patient Therapy Plan.

Cannabidiol is a medicine that comes from the cannabis plant. It does not have psychoactive (mind altering) effects. Cannabidiol needs to be prescribed by a doctor as part of an overall management plan. Cannabidiol may help to control seizures in some children with severe epilepsy where other treatments have not worked. However, as a new treatment, there is less evidence about cannabidiol compared to other medicines that are currently used to treat epilepsy.

The Single Patient Therapy Plan will assess whether cannabidiol helps reduce the number of seizures in an individual patient. It is likely that cannabidiol will have different effects for different patients; it will be helpful in some patients and not in others. After using the Single Patient Therapy Plan, the results can be used to decide for each patient whether cannabidiol is helpful and it should be continued, or whether it is not working and should be stopped.

The Single Patient Therapy Plan may be used for a child who is treated with cannabidiol as part of the Victorian Governments Compassionate Access Scheme. However, it is not a condition of the Compassionate Access Scheme that the Single Patient Therapy Plan be used, and it will be up to you and your child’s doctor to decide what is best for your child. Please discuss all options with your child’s doctor. If you don’t want your child to use the Single Patient Therapy Plan, you don’t have to. Your child will receive the best possible care whether or not you take part.

Read this information carefully and ask questions about anything that you don’t understand or want to know more about.

If you decide you want your child to try cannabidiol using the Single Patient Therapy Plan, you will be asked to sign a Consent Form. By signing it you are telling us that you understand what you have read.

You will be given a copy of this Participant Information Sheet and Consent Form to keep.

**Single Patient Therapy Plan**

**Key points**

The Single Patient Therapy Plan will assess whether cannabidiol helps to reduce seizure numbers in your child.

The Single Patient Therapy Plan runs for at least 4 months and could be up to 9 months. For approximately half of this time, your child will NOT be receiving cannabidiol. This is so your child’s doctor can compare how often your child has seizures when they are taking cannabidiol compared to when they are not.

Your child should continue to take their regular epilepsy medication whilst taking part in the Single Patient Therapy Plan. Discuss your child’s current medication with your child’s doctor who will make sure they will not interact with the cannabidiol.

Cannabidiol will be given in oil form with a peppermint taste. Parents or carers will prepare the volume of oral solution required and give it via mouth for the child to swallow or via a feeding tube.

**Details of the Single Patient Therapy Plan**

There are four main parts to the Single Patient Therapy Plan:

1. Starting Point (“Baseline”): For the first month, no cannabidiol will be given. You will be asked to record the number of seizures your child is having during this period.

2. Choosing the Dose (“Dose Selection”): Next, your child’s doctor will start and then slowly increase the dose of cannabidiol to find the right dose for your child. If your child dislikes the treatment or has unpleasant side effects, treatment may be stopped as the cannabidiol may not be right for your child. You will be able to discuss different treatment options with your child’s doctor.

3. Comparison treatment periods: Once the right dose has been selected for your child, there will be between 6 to 8 separate treatment periods, with each treatment period lasting for either 2 or 4 weeks. In each treatment period your child will receive either cannabidiol oil or a look-alike inactive oil (“placebo”). The placebo will look and taste like the cannabidiol oil but will not have cannabidiol in it, so that neither you nor your child’s doctor will know whether your child is taking cannabidiol or placebo. Your child will be taking cannabidiol for half of the treatment periods, and placebo for the other half. The order for the treatments will be chosen randomly (like a toss of a coin).

In each treatment period, you will need to record the number of seizures that your child has, as well as any side effects that you observe in a diary which will be provided to you by your child’s doctor.

This part of the Single Patient Therapy Plan will take between 3 months and 8 months, depending on how long the treatment periods are.

4. Deciding on the outcome: At the end of the treatment periods, the numbers of seizures recorded in each treatment period will be used to work out if your child had less seizures when on cannabidiol compared to placebo. This will be done using a specialised computer program at the Murdoch Children’s Research Institute (MCRI).

At this point, your child’s doctor will discuss with you whether treatment with cannabidiol is right for your child and whether it should be continued, taking into account the results from the Single Patient Therapy Plan, and any side-effects your child has experienced.

**Use of information collected as part of the Single Patient Therapy Plan**

Information collected as part of the Single Patient Therapy Plan will be kept in your child’s medical record, as is normally the case for any medical treatment. The information collected is to decide whether cannabidiol is a safe and effective treatment for your child. All information that can identify your child will remain confidential. In addition, as part of the Single Patient Therapy Plan, some of your child’s details will need to be shared for testing by the specialised computer program. These details will include: your child’s initials and date of birth, as well as the number of seizures recorded during each treatment period. This information is provided to ensure that the results are correctly returned to your child’s medical record. Any information collected that can identify your child will be treated as confidential and will be securely stored. It will be disclosed only with your permission, or as required by law.

In the future, information collected for your child as part of the Single Patient Therapy Plan may be reviewed and combined with results from other children. This would help doctors learn whether cannabidiol was effective in a group of children, to help inform treatment decisions for other children and their parents in the future. It is possible that the combined results will be shared in a variety of forums. In any publication or presentation, information will be provided in such a way that your child cannot be identified, except with your permission.

**What parents need to do**

For the Single Patient Therapy Plan to work, it needs to be followed carefully. Parents and the child will need to:

- Follow directions from your child’s doctor about when and how to take cannabidiol/placebo.
- Record all seizures and side effects in your seizure diary. Return the diary to your child’s doctor at your next appointment.
- Attend all appointments.
- Tell your child’s doctor about all of the medicines your child takes and inform/discuss any changes to these with him/her.
- Tell your child’s doctor about any other changes to your child’s health.
- Not allow anyone else to use your child’s prescription for cannabidiol/placebo.
- Return all used and unused bottles of cannabidiol/placebo to your child’s doctor/hospital pharmacist at the next appointment.

**Possible Risks**

Side effects have been in seen in the past in children treated with cannabidiol for epilepsy. Your child may have none, some or all the effects listed below, and they may be mild, moderate or severe. If your child has any of these side effects, or you are worried about them, talk with your child’s doctor. He/she will also be looking out for side effects.

Many side effects go away shortly after treatment ends. However, sometimes side effects can be serious, long lasting or permanent. If a severe side effect or reaction occurs, your child’s doctor may need to stop your child’s treatment. He/she should discuss the best way of managing any side effects with you.

Possible side effects are listed below:

- Sleep disorder; Drowsiness, Fatigue, Lethargy, Poor quality sleep, Insomnia
- Changes in appetite (increases or decreases)
- Diarrhoea, Vomiting
- Seizures
- Prolonged seizures (status epilepticus)
- Decreased energy
- Changes in weight (increases or decreases)
- Interactions with other medications

Rarer serious side effects that have been observed include:

- Liver damage
- Pneumonia (lung infections)
- Blood abnormalities (due to high ammonia or low platelets)

***You should immediately tell your child’s doctor or go to the Emergency Department at your nearest hospital if you notice any of the following:***

- Unconsciousness
- Difficulty breathing
- Severe dizziness or drowsiness
- Symptoms of allergy – for example, itchy skin rash, skin blisters, swelling around the lips or mouth, difficulty breathing

At the moment, there are no cannabis products available in Australia that have been approved by the Therapeutic Goods Administration (TGA) for use in children with severe epilepsy. As cannabidiol has not undergone formal testing of efficacy (how well it works) and safety through clinical trials, there could be other side effects that we don’t know about. It is important to record all side effects experienced by your child in the diary. Your child’s doctor will also be checking for side effects. You must bring the completed diary with you to all hospital visits.

There is also the possibility that the cannabidiol might cause problems with other medications your child is taking. Your child’s doctor will carefully review all the medications to make sure it is safe for your child to take cannabidiol. It is important that you tell your child’s doctor of all changes to medications while your child is on the Single Patient Therapy Plan.

**What will happen at the end of the Single Patient Therapy Plan?**

On completion of the Single Patient Therapy Plan, if it is decided that your child is suitable for ongoing treatment with cannabidiol.

If it is decided that cannabidiol is not beneficial for your child, then your child’s doctor will discuss other treatment options with you.

**Single Patient Therapy Plan Costs**

There are no additional costs associated with participating in the Single Patient Therapy Plan. All tests and the medical care provided as part of the approach are considered part of normal standard care provided by your child’s doctor.

**Further information and who to contact**

If you want any further information concerning the Single Patient Therapy Plan or if your child has any medical problems which may be related to your involvement in the Single Patient Therapy Plan (for example, any side effects), you can contact your child’s doctor on *[phone number]* or any of the following people:

**Clinical contact person**

| Name | *[Name]* |
| --- | --- |
| Position | *[Position]* |
| Telephone | *[Phone number]* |
| Email | *[Email address]* |

**SINGLE PATIENT THERAPY PLAN FOR THE USE OF CANNABIDIOL IN PAEDIATRIC INTRACTABLE EPILEPSY**

***CONSENT FORM***

**Declaration by Parent/Guardian:** I have read the Information Sheet, and understand the details provided.

The doctor has discussed with me the reason(s) for using cannabidiol and placebo with the Single Patient Therapy Plan, the nature and known effects of the drug, and possible risks and side effects associated with its use. I have had an opportunity to ask questions relating to the Single Patient Therapy Plan.

I understand the information collected as part of this Single Patient Therapy Plan is for the purpose of determining whether cannabidiol is effective in the treatment of my child. I consent to the use of my child’s personal and health information as described.

I understand that cannabidiol has not been approved for marketing in Australia by the Commonwealth Government, and that its effectiveness and adverse side effects have not been fully evaluated. There may be unknown risks and side effects.

I understand that I can withdraw my consent for this treatment at any time without prejudicing my child’s ongoing medical care.

| Name of Child:  (please print) |  |
| --- | --- |
| Name of Parent/Guardian:  (please print) |  |
| Signature of Parent/Guardian: |  |
| Date: |  |

**Declaration by the Doctor:** I have given a verbal explanation of the Single Patient Therapy Plan for the use of Cannabidiol in paediatric epilepsy, including the risks and I believe that the parent/guardian has understood that explanation.

| Name of Doctor:  (please print) |  |
| --- | --- |
| Signature of Doctor: |  |
| Date: |  |

**(This form is to be filed in patient’s medical record).**

**Appendix 3. Single Patient Therapy Plan Pharmacy Module**

**Disclaimer:**

These materials are provided for information and education purposes only. Information about a therapy, service, product or treatment does not in any way endorse or support such therapy, service, product or treatment, whether for any individual patient, generally or otherwise.

These materials were developed as a general informational tool to support clinical practice by qualified medical professionals considering medicinal cannabis treatment in patients with epilepsy. They are not a substitute for comprehensive individual clinical assessments and are not intended to constitute a comprehensive guide for assessing whether treatment with medicinal cannabis is or will be beneficial for any individual patient or any particular class of patients, or regarding any other aspect of treatment of patients with medicinal cannabis.

Decisions regarding treatment with medicinal cannabis, and any determination regarding whether treatment with medicinal cannabis is suitable in the circumstances, are the sole responsibility of the treating medical professional, exercising their own clinical judgment and taking into account all of the circumstances and medical history of the patient/s. For the avoidance of doubt, these materials were not developed for use by patients, and patients are solely responsible for seeking medical advice in relation to any treatment with medicinal cannabis.

The State of Victoria and the Department of Health and Human Services shall not bear any liability to you or any third party for any loss or damage which may result from your use of or reliance on any information contained in these materials, including but not limited to the use or application of any information contained in these materials, generation of data by these materials, analysis of the data generated by these materials, and/or any determinations made regarding treatment with medicinal cannabis for an individual patient, a class of patients or generally.

**SINGLE PATIENT THERAPY PLAN (N-of-1) FOR USE OF CANNABIDIOL IN PAEDIATRIC EPILEPSY**

PHARMACY MODULE

A Single Patient Therapy Plan (N-of-1) has been prepared to support the Treating Clinician and patient assess whether cannabidiol is of benefit as a treatment for severe intractable epilepsy in that individual patient. The rationale is to ensure that this vulnerable patient group has access to medicinal cannabis supported by a treatment plan for decision making, in the context of an emerging evidence base. This pharmacy module has been prepared to describe the pharmacy logistics involved.

The framework provided in the treatment plan will help in making decisions about whether a patient is benefitting from medicinal cannabis. The Single Patient Therapy Plan requires certain restrictions for patient eligibility for safety and statistical purposes. Use of this plan is at the discretion of the Treating Clinician, in conjunction with the patient and/or their caregiver.

Prior to using this Single Patient Therapy Plan, the Treating Clinician should be sufficiently trained in its use and have the support of the treating health service. The Single Patient Therapy Plan will be discussed with hospital executives and relevant committees prior to implementation.

1. INTRODUCTION

As a Schedule 4 drug, supply of cannabidiol will need to be prescribed by a medical practitioner as part of an overall treatment plan.

A Single Patient Therapy Plan (or N-of-1 trial) is an evidence-based model used for making individual patient treatment decisions^[[8]](#footnote-8)^. This model ensures that each patient receives medical care tailored to their individual needs.

The Single Patient Therapy Plan initially aims to find a safe and efficacious dose for each patient, and to identify patients who are unable to tolerate cannabidiol in an enrichment period. Patients whose epilepsy worsens, or who are unable to tolerate the cannabidiol will not proceed beyond the enrichment period. For the patients who do tolerate cannabidiol during the enrichment period, cannabidiol will be tested in a highly sensitive, repeated-crossover, placebo controlled design to determine whether treatment can be considered successful or un-successful in each individual patient.

This Pharmacy Module provides information to describe the pharmacy requirements and logistics needed to support the Treating Clinicians and patients undertaking the Single Patient Therapy Plan.

1. PRODUCT

**Description**

The product used in the Single Patient Therapy Plan is a purified cannabidiol formulation in oil for oral or enteral administration with a matching placebo oil.

To determine the maximum tolerated dose for a patient, they will complete an enrichment period which comprises of a 4-week dose finding period and a 4-week period at the selected dose.

During the enrichment period, patients will receive cannabidiol as a twice daily dose (bd), starting with a dose of 5 mg/kg/day (2.5 mg/kg twice daily) for the first week, if this is tolerated the dose will increase each week to 10 mg/kg/day (5 mg/kg twice daily), 15 mg/kg/day (7.5 mg/kg twice daily) and 20 mg/kg/day (10 mg/kg twice daily), or until undesirable side effects are observed (maximum dose 1000 mg/day). Once the maximum tolerated dose is established for the patient, the patient will be maintained on this dose for 4 weeks.

During the alternating therapy period, patients will receive cannabidiol oil or matching placebo oil bd at the established dose, according to the randomization schedule. There will be no up- or down-titration of cannabidiol oil or placebo oil, during this period, nor will there be any washout period in which the treatment will be withheld between treatment periods, i.e. patients will go straight from one treatment period to the next. Both the patient and the Treating Clinician will be blinded to the treatment allocation.

Should non-serious adverse effects occur, the dose may be decreased during the alternating treatment phases, in line with clinical judgement.

**Storage**

Refer to the required storage conditions supplied with the product.

Cannabidiol and Placebo will be stored according to the Pharmacy’s SOPs.

**Packaging and Labelling**

Cannabidiol and Placebo will be packaged and labelled by the manufacturer in line with TGA requirements. Pharmacies will re-label the product and ensure the batch number is concealed at point of dispensing according to the requirements of their SOPs.

Labelling will be performed at point of dispensing.

**Randomization and Blinding**

The enrichment period of the treatment is open-label.

The alternating therapy period of the treatment is randomized, placebo-controlled, and double-blind. The pharmacist will be unblinded to treatment allocation.

All pharmacy staff will ensure that the treatment assignment is kept securely at the pharmacy with access restricted to pharmacy staff only.

1. PROCEDURE

**Pharmacy Responsibility**

Cannabidiol and placebo will be available through the hospital pharmacy. The pharmacy is responsible for accountability, reconciliation and record maintenance.

Drug accountability, i.e. receipt, dispensing and destruction of the cannabidiol and placebo will be documented according to pharmacy SOPs.

**Communication with Treating Clinician**

The Treating Clinician will communicate with pharmacy using the hospital prescribing system.

**Treatment Sequence Allocation**

Pharmacists will be provided with a Participant Identification Form. This form should be completed for each patient.

Pharmacists will be provided with electronic files containing SPTP Patient Forms. These forms will have the randomization list embedded in the form, where treatment A indicates (active, cannabidiol) and B indicates (placebo to match). The pharmacist will sequentially allocate a unique identification number to each patient when they enter the alternating therapy period by accessing the NEXT randomised SPTP Patient sequence page.

Patients will be required to return to the hospital for a visit at completion of each treatment period. They will be asked to return all previously dispensed bottles. Patients are to be informed that **NO** bottle can be ‘lost’ and **all** bottles (used and unused) must be returned before new bottles are dispensed. **If any bottles are not returned, the patient may return at a later date to collect new supplies**, this will not adversely affect the analysis of the results.

**Code Break Procedure**

The Treating Clinician will remain blinded, unless knowledge of the patient’s treatment assignment is necessary for the clinical management or welfare of the subject, or for safety reporting to the TGA. In the event that un-blinding is deemed necessary for a patient, the pharmacist will provide details of treatment allocation to the treating clinician. The details and reason for un-blinding that patient will be clearly documented.

**Appendix 4. Patient Seizure Diary Baseline, Dose Finding, Maintenance**

**Disclaimer:**

These materials are provided for information and education purposes only. Information about a therapy, service, product or treatment does not in any way endorse or support such therapy, service, product or treatment, whether for any individual patient, generally or otherwise.

These materials were developed as a general informational tool to support clinical practice by qualified medical professionals considering medicinal cannabis treatment in patients with epilepsy. They are not a substitute for comprehensive individual clinical assessments and are not intended to constitute a comprehensive guide for assessing whether treatment with medicinal cannabis is or will be beneficial for any individual patient or any particular class of patients, or regarding any other aspect of treatment of patients with medicinal cannabis.

Decisions regarding treatment with medicinal cannabis, and any determination regarding whether treatment with medicinal cannabis is suitable in the circumstances, are the sole responsibility of the treating medical professional, exercising their own clinical judgment and taking into account all of the circumstances and medical history of the patient/s. For the avoidance of doubt, these materials were not developed for use by patients, and patients are solely responsible for seeking medical advice in relation to any treatment with medicinal cannabis.

The State of Victoria and the Department of Health and Human Services shall not bear any liability to you or any third party for any loss or damage which may result from your use of or reliance on any information contained in these materials, including but not limited to the use or application of any information contained in these materials, generation of data by these materials, analysis of the data generated by these materials, and/or any determinations made regarding treatment with medicinal cannabis for an individual patient, a class of patients or generally.

**PATIENT DIARY – BASELINE, DOSE FINDING AND MAINTENANCE**

**Contact Details at [Site]: [Name and Phone] Patient Name: ­­­­­_______________________**

- *If your child is admitted to hospital, remember to bring your own supply of medication from this Single Patient Therapy Plan along with this patient diary and your Wallet Card.*

| **VISIT 1**  (_ _/_ _/_ _) | **Start of Baseline Period: 4 weeks (Day B-1 to Day B-28)**   - Meet with your Clinician to discuss the Single Patient Therapy Plan - Informed consent (parents or patient) - Collect Diary - Next Appointment in 1 month: Date & Time: ________________________________ |
| --- | --- |

| **VISIT 2** Day DF-1  (_ _/_ _/_ _) | | **Start of Dose Finding Period: 4 weeks (Day DF-1 to Day DF-28)**   - **Remember to bring this patient diary to the appointment**. - Meet with your Clinician to review the diary - Collect medication (Cannabidiol) from Pharmacy - Dose of Cannabidiol: ­­­­­­­­­­­­­­­­­­­­­­5 mg/kg/day - Record side effects in this patient diary - Record any new medications in this patient diary - Next Appointment in 1 month: Date & Time: _________________________________ |
| --- | --- | --- |
| **INSTRUCTIONS FOR UPTITRATION** | Day DF-8  (_ _/_ _/_ _) | - Contact your Support Person for titration decision: - Withdrawal from the treatment plan. - 5 mg/kg/day - 10 mg/kg/day. - Record side effects in the diary. - Record any new medications in the diary. |
|  | Day DF-15  (_ _/_ _/_ _) | - Contact your Support Person for titration decision: - 5 mg/kg/day - 10 mg/kg/day. - 15 mg/kg/day. - Record side effects in the diary. - Record any new medications in the diary. |
|  | Day DF-22  (_ _/_ _/_ _) | - Contact your Support Person for titration decision: - 5 mg/kg/day. - 10 mg/kg/day. - 15 mg/kg/day - 20 mg/kg/day - Record side effects in the diary. - Record any new medications in the diary. |

| **VISIT 3**  Day M-1 (_ _/_ _/_ _) | **Start of Maintenance Period: 4 weeks (Day M-1 to Day M-28)**   - **Remember to bring this patient diary to the appointment**. - **Please remember to bring all used and unused medication to the pharmacy.** - Meet with your Clinician to review the diary - Selected dose of Cannabadiol:_________________________________________ - Collect medication (Cannabidiol) from Pharmacy - Collect Blood Test Request Form - Record side effects in the diary. - Record any new medications in the diary.   Next Appointment in 1 month: Date & Time: ________________________________ |
| --- | --- |

**Side Effects**: Please record all side effects observed during the Dose Finding and Maintenance Periods

| Start Date | Stop Date | Describe Side effect |
| --- | --- | --- |
|  |  |  |
|  |  |  |
|  |  |  |
|  |  |  |
|  |  |  |
|  |  |  |
|  |  |  |
|  |  |  |
|  |  |  |
|  |  |  |
|  |  |  |
|  |  |  |
|  |  |  |
|  |  |  |
|  |  |  |
|  |  |  |
|  |  |  |
|  |  |  |

**Medications**: Please record any **new** medicines your child has taken during the Dose Finding and Maintenance Periods

| Start Date | Stop Date | Dose | Name of Medicine |
| --- | --- | --- | --- |
|  |  |  |  |
|  |  |  |  |
|  |  |  |  |
|  |  |  |  |
|  |  |  |  |
|  |  |  |  |
|  |  |  |  |
|  |  |  |  |
|  |  |  |  |
|  |  |  |  |
|  |  |  |  |
|  |  |  |  |
|  |  |  |  |
|  |  |  |  |

# Baseline - Week 1 Date:_________________

Seizures: Please record seizure types and keep a tally of seizures observed daily in the week

|  | Day B-1 | Day B-2 | Day B-3 | Day B-4 | Day B-5 | Day B-6 | Day B-7 |
| --- | --- | --- | --- | --- | --- | --- | --- |
| Seizure Type: | | | | | | | |
| Tally |  |  |  |  |  |  |  |
| Total |  |  |  |  |  |  |  |
| Seizure Type: | | | | | | | |
| Tally |  |  |  |  |  |  |  |
| Total |  |  |  |  |  |  |  |
| Seizure Type: | | | | | | | |
| Tally |  |  |  |  |  |  |  |
| Total |  |  |  |  |  |  |  |
| Seizure Type: | | | | | | | |
| Tally |  |  |  |  |  |  |  |
| Total |  |  |  |  |  |  |  |
| Seizure Type: | | | | | | | |
| Tally |  |  |  |  |  |  |  |
| Total |  |  |  |  |  |  |  |
| Seizure Type: | | | | | | | |
| Tally |  |  |  |  |  |  |  |
| Total |  |  |  |  |  |  |  |

Medications: Please record all antiepileptic drugs your child is taking during this period.

| Start Date | Stop Date | Dose | Name of Medicine |
| --- | --- | --- | --- |
|  |  |  |  |
|  |  |  |  |
|  |  |  |  |
|  |  |  |  |
|  |  |  |  |
|  |  |  |  |
|  |  |  |  |
|  |  |  |  |

# Baseline - Week 2 Date:_________________

Seizures: Please record seizure types and keep a tally of seizures observed daily in the week

|  | Day B-8 | Day B-9 | Day B-10 | Day B-11 | Day B-12 | Day B-13 | Day B-14 |
| --- | --- | --- | --- | --- | --- | --- | --- |
| Seizure Type: | | | | | | | |
| Tally |  |  |  |  |  |  |  |
| Total |  |  |  |  |  |  |  |
| Seizure Type: | | | | | | | |
| Tally |  |  |  |  |  |  |  |
| Total |  |  |  |  |  |  |  |
| Seizure Type: | | | | | | | |
| Tally |  |  |  |  |  |  |  |
| Total |  |  |  |  |  |  |  |
| Seizure Type: | | | | | | | |
| Tally |  |  |  |  |  |  |  |
| Total |  |  |  |  |  |  |  |
| Seizure Type: | | | | | | | |
| Tally |  |  |  |  |  |  |  |
| Total |  |  |  |  |  |  |  |
| Seizure Type: | | | | | | | |
| Tally |  |  |  |  |  |  |  |
| Total |  |  |  |  |  |  |  |

Medications: Please record all antiepileptic drugs your child is taking during this period.

| Start Date | Stop Date | Dose | Name of Medicine |
| --- | --- | --- | --- |
|  |  |  |  |
|  |  |  |  |
|  |  |  |  |
|  |  |  |  |
|  |  |  |  |
|  |  |  |  |
|  |  |  |  |
|  |  |  |  |

# Baseline - Week 3 Date:_________________

Seizures: Please record seizure types and keep a tally of seizures observed daily in the week

|  | Day B-15 | Day B-16 | Day B-17 | Day B-18 | Day B-19 | Day B-20 | Day B-21 |
| --- | --- | --- | --- | --- | --- | --- | --- |
| Seizure Type: | | | | | | | |
| Tally |  |  |  |  |  |  |  |
| Total |  |  |  |  |  |  |  |
| Seizure Type: | | | | | | | |
| Tally |  |  |  |  |  |  |  |
| Total |  |  |  |  |  |  |  |
| Seizure Type: | | | | | | | |
| Tally |  |  |  |  |  |  |  |
| Total |  |  |  |  |  |  |  |
| Seizure Type: | | | | | | | |
| Tally |  |  |  |  |  |  |  |
| Total |  |  |  |  |  |  |  |
| Seizure Type: | | | | | | | |
| Tally |  |  |  |  |  |  |  |
| Total |  |  |  |  |  |  |  |
| Seizure Type: | | | | | | | |
| Tally |  |  |  |  |  |  |  |
| Total |  |  |  |  |  |  |  |

Medications: Please record all antiepileptic drugs your child is taking during this period.

| Start Date | Stop Date | Dose | Name of Medicine |
| --- | --- | --- | --- |
|  |  |  |  |
|  |  |  |  |
|  |  |  |  |
|  |  |  |  |
|  |  |  |  |
|  |  |  |  |
|  |  |  |  |
|  |  |  |  |

# Baseline - Week 4 Date:_________________

Seizures: Please record seizure types and keep a tally of seizures observed daily in the week

|  | Day B-22 | Day B-23 | Day B-24 | Day B-25 | Day B-26 | Day B-27 | Day B-28 |
| --- | --- | --- | --- | --- | --- | --- | --- |
| Seizure Type: | | | | | | | |
| Tally |  |  |  |  |  |  |  |
| Total |  |  |  |  |  |  |  |
| Seizure Type: | | | | | | | |
| Tally |  |  |  |  |  |  |  |
| Total |  |  |  |  |  |  |  |
| Seizure Type: | | | | | | | |
| Tally |  |  |  |  |  |  |  |
| Total |  |  |  |  |  |  |  |
| Seizure Type: | | | | | | | |
| Tally |  |  |  |  |  |  |  |
| Total |  |  |  |  |  |  |  |
| Seizure Type: | | | | | | | |
| Tally |  |  |  |  |  |  |  |
| Total |  |  |  |  |  |  |  |
| Seizure Type: | | | | | | | |
| Tally |  |  |  |  |  |  |  |
| Total |  |  |  |  |  |  |  |

Medications: Please record all antiepileptic drugs your child is taking during this period.

| Start Date | Stop Date | Dose | Name of Medicine |
| --- | --- | --- | --- |
|  |  |  |  |
|  |  |  |  |
|  |  |  |  |
|  |  |  |  |
|  |  |  |  |
|  |  |  |  |
|  |  |  |  |
|  |  |  |  |

# Baseline – Additional Page, use only if needed Date:_________________

Seizures: Please record seizure types and keep a tally of seizures observed daily in the week

|  | Day B-29 | Day B-30 | Day B-31 | Day B-32 | Day B-33 | Day B-34 | Day B-35 |
| --- | --- | --- | --- | --- | --- | --- | --- |
| Seizure Type: | | | | | | | |
| Tally |  |  |  |  |  |  |  |
| Total |  |  |  |  |  |  |  |
| Seizure Type: | | | | | | | |
| Tally |  |  |  |  |  |  |  |
| Total |  |  |  |  |  |  |  |
| Seizure Type: | | | | | | | |
| Tally |  |  |  |  |  |  |  |
| Total |  |  |  |  |  |  |  |
| Seizure Type: | | | | | | | |
| Tally |  |  |  |  |  |  |  |
| Total |  |  |  |  |  |  |  |
| Seizure Type: | | | | | | | |
| Tally |  |  |  |  |  |  |  |
| Total |  |  |  |  |  |  |  |
| Seizure Type: | | | | | | | |
| Tally |  |  |  |  |  |  |  |
| Total |  |  |  |  |  |  |  |

Medications: Please record all antiepileptic drugs your child is taking during this period.

| Start Date | Stop Date | Dose | Name of Medicine |
| --- | --- | --- | --- |
|  |  |  |  |
|  |  |  |  |
|  |  |  |  |
|  |  |  |  |
|  |  |  |  |
|  |  |  |  |
|  |  |  |  |
|  |  |  |  |

# Dose Finding - Week 1 Date: ______________________________________

Total Dose: _________ Dose is ______ mL twice per day.

Two doses to be administered each day, within 30 minutes of consuming a main meal (e.g. breakfast or dinner). If a dose is missed **DO NOT** take twice the amount to make up for the missed dose. Please write ‘Dose Missed’ with a reason why.

| Date | Dose 1 Time | Dose 2 Time | Comments/problems with dose |
| --- | --- | --- | --- |
| Day DF-1 |  |  |  |
| Day DF-2 |  |  |  |
| Day DF-3 |  |  |  |
| Day DF-4 |  |  |  |
| Day DF-5 |  |  |  |
| Day DF-6 |  |  |  |
| Day DF-7 |  |  |  |

Seizures: Please record seizure types and keep a tally of seizures observed

| Seizure | Day DF-1 | Day DF-2 | Day DF-3 | Day DF-4 | Day DF-5 | Day DF-6 | Day DF-7 |
| --- | --- | --- | --- | --- | --- | --- | --- |
| Type: | | | | | | | |
| Tally |  |  |  |  |  |  |  |
| Total |  |  |  |  |  |  |  |
| Type: | | | | | | | |
| Tally |  |  |  |  |  |  |  |
| Total |  |  |  |  |  |  |  |
| Type: | | | | | | | |
| Tally |  |  |  |  |  |  |  |
| Total |  |  |  |  |  |  |  |
| Type: | | | | | | | |
| Tally |  |  |  |  |  |  |  |
| Total |  |  |  |  |  |  |  |
| Type: | | | | | | | |
| Tally |  |  |  |  |  |  |  |
| Total |  |  |  |  |  |  |  |
| Type: | | | | | | | |
| Tally |  |  |  |  |  |  |  |
| Total |  |  |  |  |  |  |  |

# Dose Finding - Week 2 Date: ______________________________________

Total Dose: _________ Dose is ______ mL twice per day.

Two doses to be administered each day, within 30 minutes of consuming a main meal (e.g. breakfast or dinner). If a dose is missed **DO NOT** take twice the amount to make up for the missed dose. Please write ‘Dose Missed’ with a reason why.

| Date | Dose 1 Time | Dose 2 Time | Comments/problems with dose |
| --- | --- | --- | --- |
| Day DF-8 |  |  |  |
| Day DF-9 |  |  |  |
| Day DF-10 |  |  |  |
| Day DF-11 |  |  |  |
| Day DF-12 |  |  |  |
| Day DF-13 |  |  |  |
| Day DF-14 |  |  |  |

Seizures: Please record seizure types and keep a tally of seizures observed

| Seizure | Day DF-8 | Day DF-9 | Day DF-10 | Day DF-11 | Day DF-12 | Day DF-13 | Day DF-14 |
| --- | --- | --- | --- | --- | --- | --- | --- |
| Type: | | | | | | | |
| Tally |  |  |  |  |  |  |  |
| Total |  |  |  |  |  |  |  |
| Type: | | | | | | | |
| Tally |  |  |  |  |  |  |  |
| Total |  |  |  |  |  |  |  |
| Type: | | | | | | | |
| Tally |  |  |  |  |  |  |  |
| Total |  |  |  |  |  |  |  |
| Type: | | | | | | | |
| Tally |  |  |  |  |  |  |  |
| Total |  |  |  |  |  |  |  |
| Type: | | | | | | | |
| Tally |  |  |  |  |  |  |  |
| Total |  |  |  |  |  |  |  |
| Type: | | | | | | | |
| Tally |  |  |  |  |  |  |  |
| Total |  |  |  |  |  |  |  |

# Dose Finding - Week 3 Date: ______________________________________

Total Dose: _________ Dose is ______ mL twice per day.

Two doses to be administered each day, within 30 minutes of consuming a main meal (e.g. breakfast or dinner). If a dose is missed **DO NOT** take twice the amount to make up for the missed dose. Please write ‘Dose Missed’ with a reason why.

| Date | Dose 1 Time | Dose 2 Time | Comments/problems with dose |
| --- | --- | --- | --- |
| Day DF-15 |  |  |  |
| Day DF-16 |  |  |  |
| Day DF-17 |  |  |  |
| Day DF-18 |  |  |  |
| Day DF-19 |  |  |  |
| Day DF-20 |  |  |  |
| Day DF-21 |  |  |  |

Seizures: Please record seizure types and keep a tally of seizures observed

| Seizure | Day DF-15 | Day DF-16 | Day DF-17 | Day DF-18 | Day DF-19 | Day DF-20 | Day DF-21 |
| --- | --- | --- | --- | --- | --- | --- | --- |
| Type: | | | | | | | |
| Tally |  |  |  |  |  |  |  |
| Total |  |  |  |  |  |  |  |
| Type: | | | | | | | |
| Tally |  |  |  |  |  |  |  |
| Total |  |  |  |  |  |  |  |
| Type: | | | | | | | |
| Tally |  |  |  |  |  |  |  |
| Total |  |  |  |  |  |  |  |
| Type: | | | | | | | |
| Tally |  |  |  |  |  |  |  |
| Total |  |  |  |  |  |  |  |
| Type: | | | | | | | |
| Tally |  |  |  |  |  |  |  |
| Total |  |  |  |  |  |  |  |
| Type: | | | | | | | |
| Tally |  |  |  |  |  |  |  |
| Total |  |  |  |  |  |  |  |

# Dose Finding - Week 4 Date: ______________________________________

Total Dose: _________ Dose is ______ mL twice per day.

Two doses to be administered each day, within 30 minutes of consuming a main meal (e.g. breakfast or dinner). If a dose is missed **DO NOT** take twice the amount to make up for the missed dose. Please write ‘Dose Missed’ with a reason why.

| Date | Dose 1 Time | Dose 2 Time | Comments/problems with dose |
| --- | --- | --- | --- |
| Day DF-22 |  |  |  |
| Day DF-23 |  |  |  |
| Day DF-24 |  |  |  |
| Day DF-25 |  |  |  |
| Day DF-26 |  |  |  |
| Day DF-27 |  |  |  |
| Day DF-28 |  |  |  |

Seizures: Please record seizure types and keep a tally of seizures observed

| Seizure | Day DF-22 | Day DF-23 | Day DF-24 | Day DF-25 | Day DF-26 | Day DF-27 | Day DF-28 |
| --- | --- | --- | --- | --- | --- | --- | --- |
| Type: | | | | | | | |
| Tally |  |  |  |  |  |  |  |
| Total |  |  |  |  |  |  |  |
| Type: | | | | | | | |
| Tally |  |  |  |  |  |  |  |
| Total |  |  |  |  |  |  |  |
| Type: | | | | | | | |
| Tally |  |  |  |  |  |  |  |
| Total |  |  |  |  |  |  |  |
| Type: | | | | | | | |
| Tally |  |  |  |  |  |  |  |
| Total |  |  |  |  |  |  |  |
| Type: | | | | | | | |
| Tally |  |  |  |  |  |  |  |
| Total |  |  |  |  |  |  |  |
| Type: | | | | | | | |
| Tally |  |  |  |  |  |  |  |
| Total |  |  |  |  |  |  |  |

# Dose Finding - Additional Page, use only if needed Date: ______________________________________

Total Dose: _________ Dose is ______ mL twice per day.

Two doses to be administered each day, within 30 minutes of consuming a main meal (e.g. breakfast or dinner). If a dose is missed **DO NOT** take twice the amount to make up for the missed dose. Please write ‘Dose Missed’ with a reason why.

| Date | Dose 1 Time | Dose 2 Time | Comments/problems with dose |
| --- | --- | --- | --- |
| Day DF-29 |  |  |  |
| Day DF-30 |  |  |  |
| Day DF-31 |  |  |  |
| Day DF-32 |  |  |  |
| Day DF-33 |  |  |  |
| Day DF-34 |  |  |  |
| Day DF-35 |  |  |  |

Seizures: Please record seizure types and keep a tally of seizures observed

| Seizure | Day DF-29 | Day DF-30 | Day DF-31 | Day DF-32 | Day DF-33 | Day DF-34 | Day DF-35 |
| --- | --- | --- | --- | --- | --- | --- | --- |
| Type: | | | | | | | |
| Tally |  |  |  |  |  |  |  |
| Total |  |  |  |  |  |  |  |
| Type: | | | | | | | |
| Tally |  |  |  |  |  |  |  |
| Total |  |  |  |  |  |  |  |
| Type: | | | | | | | |
| Tally |  |  |  |  |  |  |  |
| Total |  |  |  |  |  |  |  |
| Type: | | | | | | | |
| Tally |  |  |  |  |  |  |  |
| Total |  |  |  |  |  |  |  |
| Type: | | | | | | | |
| Tally |  |  |  |  |  |  |  |
| Total |  |  |  |  |  |  |  |
| Type: | | | | | | | |
| Tally |  |  |  |  |  |  |  |
| Total |  |  |  |  |  |  |  |

# Maintenance - Week 1 Date: ______________________________

Total Dose: _________mg/kg/day. Dose is ______ mL twice per day.

Two doses to be administered each day, within 30 minutes of consuming a main meal (e.g. breakfast or dinner). If a dose is missed **DO NOT** take twice the amount to make up for the missed dose. Please write ‘Dose Missed’ with a reason why.

| Date | Dose 1 Time | Dose 2 Time | Comments/problems with dose |
| --- | --- | --- | --- |
| Day M-1 |  |  |  |
| Day M-2 |  |  |  |
| Day M-3 |  |  |  |
| Day M-4 |  |  |  |
| Day M-5 |  |  |  |
| Day M-6 |  |  |  |
| Day M-7 |  |  |  |

Seizures: Please record seizure types and keep a tally of seizures observed

| Seizure | Day M-1 | Day M-2 | Day M-3 | Day M-4 | Day M-5 | Day M-6 | Day M-7 |
| --- | --- | --- | --- | --- | --- | --- | --- |
| Type: | | | | | | | |
| Tally |  |  |  |  |  |  |  |
| Total |  |  |  |  |  |  |  |
| Type: | | | | | | | |
| Tally |  |  |  |  |  |  |  |
| Total |  |  |  |  |  |  |  |
| Type: | | | | | | | |
| Tally |  |  |  |  |  |  |  |
| Total |  |  |  |  |  |  |  |
| Type: | | | | | | | |
| Tally |  |  |  |  |  |  |  |
| Total |  |  |  |  |  |  |  |
| Type: | | | | | | | |
| Tally |  |  |  |  |  |  |  |
| Total |  |  |  |  |  |  |  |
| Type: | | | | | | | |
| Tally |  |  |  |  |  |  |  |
| Total |  |  |  |  |  |  |  |

# Maintenance - Week 2 Date: ______________________________

Total Dose: _________mg/kg/day. Dose is ______ mL twice per day.

Two doses to be administered each day, within 30 minutes of consuming a main meal (e.g. breakfast or dinner). If a dose is missed **DO NOT** take twice the amount to make up for the missed dose. Please write ‘Dose Missed’ with a reason why.

| Date | Dose 1 Time | Dose 2 Time | Comments/problems with dose |
| --- | --- | --- | --- |
| Day M-8 |  |  |  |
| Day M-9 |  |  |  |
| Day M-10 |  |  |  |
| Day M-11 |  |  |  |
| Day M-12 |  |  |  |
| Day M-13 |  |  |  |
| Day M-14 |  |  |  |

Seizures: Please record seizure types and keep a tally of seizures observed

| Seizure | Day M-8 | Day M-9 | Day M-10 | Day M-11 | Day M-12 | Day M-13 | Day M-14 |
| --- | --- | --- | --- | --- | --- | --- | --- |
| Type: | | | | | | | |
| Tally |  |  |  |  |  |  |  |
| Total |  |  |  |  |  |  |  |
| Type: | | | | | | | |
| Tally |  |  |  |  |  |  |  |
| Total |  |  |  |  |  |  |  |
| Type: | | | | | | | |
| Tally |  |  |  |  |  |  |  |
| Total |  |  |  |  |  |  |  |
| Type: | | | | | | | |
| Tally |  |  |  |  |  |  |  |
| Total |  |  |  |  |  |  |  |
| Type: | | | | | | | |
| Tally |  |  |  |  |  |  |  |
| Total |  |  |  |  |  |  |  |
| Type: | | | | | | | |
| Tally |  |  |  |  |  |  |  |
| Total |  |  |  |  |  |  |  |

# Maintenance - Week 3 Date: ______________________________

Total Dose: _________mg/kg/day. Dose is ______ mL twice per day.

Two doses to be administered each day, within 30 minutes of consuming a main meal (e.g. breakfast or dinner). If a dose is missed **DO NOT** take twice the amount to make up for the missed dose. Please write ‘Dose Missed’ with a reason why.

| Date | Dose 1 Time | Dose 2 Time | Comments/problems with dose |
| --- | --- | --- | --- |
| Day M-15 |  |  |  |
| Day M-16 |  |  |  |
| Day M-17 |  |  |  |
| Day M-18 |  |  |  |
| Day M-19 |  |  |  |
| Day M-20 |  |  |  |
| Day M-21 |  |  |  |

Seizures: Please record seizure types and keep a tally of seizures observed

| Seizure | Day M-15 | Day M-16 | Day M-17 | Day M-18 | Day M-19 | Day M-20 | Day M-21 |
| --- | --- | --- | --- | --- | --- | --- | --- |
| Type: | | | | | | | |
| Tally |  |  |  |  |  |  |  |
| Total |  |  |  |  |  |  |  |
| Type: | | | | | | | |
| Tally |  |  |  |  |  |  |  |
| Total |  |  |  |  |  |  |  |
| Type: | | | | | | | |
| Tally |  |  |  |  |  |  |  |
| Total |  |  |  |  |  |  |  |
| Type: | | | | | | | |
| Tally |  |  |  |  |  |  |  |
| Total |  |  |  |  |  |  |  |
| Type: | | | | | | | |
| Tally |  |  |  |  |  |  |  |
| Total |  |  |  |  |  |  |  |
| Type: | | | | | | | |
| Tally |  |  |  |  |  |  |  |
| Total |  |  |  |  |  |  |  |

# Maintenance - Week 4 Date: ______________________________

Total Dose: _________mg/kg/day. Dose is ______ mL twice per day.

Two doses to be administered each day, within 30 minutes of consuming a main meal (e.g. breakfast or dinner). If a dose is missed **DO NOT** take twice the amount to make up for the missed dose. Please write ‘Dose Missed’ with a reason why.

| Date | Dose 1 Time | Dose 2 Time | Comments/problems with dose |
| --- | --- | --- | --- |
| Day M-22 |  |  |  |
| Day M-23 |  |  |  |
| Day M-24 |  |  |  |
| Day M-25 |  |  |  |
| Day M-26 |  |  |  |
| Day M-27 |  |  |  |
| Day M-28 |  |  |  |

Seizure Diary: Please record seizure types and keep a tally of seizures observed

| Seizure | Day M-22 | Day M-23 | Day M-24 | Day M-25 | Day M-26 | Day M-27 | Day M-28 |
| --- | --- | --- | --- | --- | --- | --- | --- |
| Type: | | | | | | | |
| Tally |  |  |  |  |  |  |  |
| Total |  |  |  |  |  |  |  |
| Type: | | | | | | | |
| Tally |  |  |  |  |  |  |  |
| Total |  |  |  |  |  |  |  |
| Type: | | | | | | | |
| Tally |  |  |  |  |  |  |  |
| Total |  |  |  |  |  |  |  |
| Type: | | | | | | | |
| Tally |  |  |  |  |  |  |  |
| Total |  |  |  |  |  |  |  |
| Type: | | | | | | | |
| Tally |  |  |  |  |  |  |  |
| Total |  |  |  |  |  |  |  |
| Type: | | | | | | | |
| Tally |  |  |  |  |  |  |  |
| Total |  |  |  |  |  |  |  |

# Maintenance - Additional Page, use only if needed Date: ______________________________

Total Dose: _________mg/kg/day. Dose is ______ mL twice per day.

Two doses to be administered each day, within 30 minutes of consuming a main meal (e.g. breakfast or dinner). If a dose is missed **DO NOT** take twice the amount to make up for the missed dose. Please write ‘Dose Missed’ with a reason why.

| Date | Dose 1 Time | Dose 2 Time | Comments/problems with dose |
| --- | --- | --- | --- |
| Day M-29 |  |  |  |
| Day M-30 |  |  |  |
| Day M-31 |  |  |  |
| Day M-32 |  |  |  |
| Day M-33 |  |  |  |
| Day M-34 |  |  |  |
| Day M-35 |  |  |  |

Seizures: Please record seizure types and keep a tally of seizures observed

| Seizure | Day M-29 | Day M-30 | Day M-31 | Day M-32 | Day M-33 | Day M-34 | Day M-35 |
| --- | --- | --- | --- | --- | --- | --- | --- |
| Type: | | | | | | | |
| Tally |  |  |  |  |  |  |  |
| Total |  |  |  |  |  |  |  |
| Type: | | | | | | | |
| Tally |  |  |  |  |  |  |  |
| Total |  |  |  |  |  |  |  |
| Type: | | | | | | | |
| Tally |  |  |  |  |  |  |  |
| Total |  |  |  |  |  |  |  |
| Type: | | | | | | | |
| Tally |  |  |  |  |  |  |  |
| Total |  |  |  |  |  |  |  |
| Type: | | | | | | | |
| Tally |  |  |  |  |  |  |  |
| Total |  |  |  |  |  |  |  |
| Type: | | | | | | | |
| Tally |  |  |  |  |  |  |  |
| Total |  |  |  |  |  |  |  |

**Appendix 5. Patient Seizure Diary Alternating Therapy Period (2 week treatment period)**

**Disclaimer:**

These materials are provided for information and education purposes only. Information about a therapy, service, product or treatment does not in any way endorse or support such therapy, service, product or treatment, whether for any individual patient, generally or otherwise.

These materials were developed as a general informational tool to support clinical practice by qualified medical professionals considering medicinal cannabis treatment in patients with epilepsy. They are not a substitute for comprehensive individual clinical assessments and are not intended to constitute a comprehensive guide for assessing whether treatment with medicinal cannabis is or will be beneficial for any individual patient or any particular class of patients, or regarding any other aspect of treatment of patients with medicinal cannabis.

Decisions regarding treatment with medicinal cannabis, and any determination regarding whether treatment with medicinal cannabis is suitable in the circumstances, are the sole responsibility of the treating medical professional, exercising their own clinical judgment and taking into account all of the circumstances and medical history of the patient/s. For the avoidance of doubt, these materials were not developed for use by patients, and patients are solely responsible for seeking medical advice in relation to any treatment with medicinal cannabis.

The State of Victoria and the Department of Health and Human Services shall not bear any liability to you or any third party for any loss or damage which may result from your use of or reliance on any information contained in these materials, including but not limited to the use or application of any information contained in these materials, generation of data by these materials, analysis of the data generated by these materials, and/or any determinations made regarding treatment with medicinal cannabis for an individual patient, a class of patients or generally.

**PATIENT DIARY – ALTERNATING THERAPY PERIOD**

**2 WEEK TREATMENT PERIOD**

**Contact Details at [Site]:** **[Name and Phone] Patients Name:**

- *At each visit, you must remember to return all used and unused medication bottles to the pharmacy. Pharmacy cannot dispense more without receiving the old bottles.*
- *If you’re admitted to hospital, remember to bring your own supply of medication from this Single Patient Therapy Plan along with this patient diary and your Wallet Card.*

| **ALTERNATING THERAPY PERIOD VISITS (6 TREATMENT PERIODS OF 2 WEEKS DURATION)** | |
| --- | --- |
| **VISIT 4** (_ _/_ _/_ _) | - **Please remember to bring this patient diary to the appointment**. - **Please remember to bring all used and unused medication to the pharmacy.** - Meet with your Clinician to review the diary - Collect medication (Cannabidiol/Placebo) from Pharmacy - Next Pharmacy Appointment in 2 weeks: Date: _______________________________ - Next Clinic Appointment in 4 weeks: Date & Time: ________________________________ |
| **VISIT 5** (_ _/_ _/_ _)  **PHARMACY ONLY** | - **Please remember to bring all used and unused medication to the pharmacy.** - Collect medication (Cannabidiol/Placebo) from Pharmacy - Next Pharmacy Appointment in 2 weeks: Date & Time: ________________________________ |
| **VISIT 6** (_ _/_ _/_ _) | - **Please remember to bring this patient diary to the appointment**. - **Please remember to bring all used and unused medication to the pharmacy.** - Meet with your Clinician to review the diary - Collect medication (Cannabidiol/Placebo) from Pharmacy - Collect Blood Test Request Form and have bloods taken prior to your next clinic appointment - Next Pharmacy Appointment in 2 weeks: Date:_______________________________ - Next Clinic Appointment in 4 weeks: Date & Time: ________________________________ |
| **VISIT 7** (_ _/_ _/_ _)  **PHARMACY ONLY** | - **Please remember to bring all used and unused medication to the pharmacy.** - Collect medication (Cannabidiol/Placebo) from Pharmacy - Next Pharmacy Appointment in 2 weeks: Date & Time: ________________________________ |
| **VISIT 8** (_ _/_ _/_ _) | - **Please remember to bring this patient diary to the appointment**. - **Please remember to bring all used and unused medication to the pharmacy.** - Meet with your Clinician to review the diary - Collect medication (Cannabidiol/Placebo) from Pharmacy - Collect Blood Test Request Form and have bloods taken prior to your next clinic appointment - Next Pharmacy Appointment in 2 weeks: Date: ________________________________ - Next Clinic Appointment in 4 weeks: Date & Time: ________________________________ |
| **VISIT 9** (_ _/_ _/_ _)  **PHARMACY ONLY** | - **Please remember to bring all used and unused medication to the pharmacy.** - Collect medication (Cannabidiol/Placebo) from Pharmacy - Next Pharmacy Appointment in 2 weeks: Date: ________________________________ |

| **VISIT 10** (_ _/_ _/_ _) | - **Please remember to bring this patient diary to the appointment**. - **Please remember to bring all used and unused medication to the pharmacy.** - Meet with your Clinician to review the diary - Determine with Clinician if optional treatment periods are needed: YES or NO   **If YES:**   - Collect medication (Cannabidiol/Placebo) from Pharmacy - Next Pharmacy Appointment in 2 weeks: Date: ________________________________   **If NO:**   - Next Clinic Appointment in 4 weeks: Date & Time: ______________________________ |
| --- | --- |

| **ALTERNATING THERAPY PERIODS 7 & 8 (USE ONLY IF YOUR CLINICIAN SAYS THEY ARE NEEDED)** | |
| --- | --- |
| **VISIT DATE:**  (_ _/_ _/_ _)  **PHARMACY ONLY**  **For Alternating Therapy Period 7** | - **Please remember to return all used and unused medication to the pharmacy.** - Collect medication (Cannabidiol/Placebo) from Pharmacy - Next Pharmacy Appointment in 2 weeks: Date: ________________________________ |
| **VISIT DATE:**  (_ _/_ _/_ _)  **For Alternating Therapy Period 8** | - **Please remember to bring this patient diary to the appointment**. - **Please remember to return all used and unused medication to the pharmacy.** - Meet with your Clinician to review the diary - Collect medication (Cannabidiol/Placebo) from Pharmacy - Next Clinic Appointment in 4 weeks: Date & Time: ________________________________ |

| **RESULTS VISIT** | |
| --- | --- |
| **VISIT 11**  (_ _/_ _/_ _) | - **Please remember to bring this patient diary to the appointment**. - **Please remember to return all used and unused medication to the pharmacy.** - Meet with your Clinician to review the diary - Collect medication (Cannabidiol) from Pharmacy, if required |

| **UNSCHEDULED VISITS** | |
| --- | --- |
|  | - **Please remember to bring this patient diary to the appointment**. - **Please remember to return all used and unused medication to the pharmacy.** - Meet with your Clinician to discuss the reason for the visit - Collect medication (Cannabidiol) from Pharmacy, if required |

# Side Effects: Please record all side effects observed

| Start Date | Stop Date | Describe Side effect |
| --- | --- | --- |
|  |  |  |
|  |  |  |
|  |  |  |
|  |  |  |
|  |  |  |
|  |  |  |
|  |  |  |
|  |  |  |
|  |  |  |
|  |  |  |
|  |  |  |
|  |  |  |
|  |  |  |
|  |  |  |
|  |  |  |
|  |  |  |
|  |  |  |
|  |  |  |
|  |  |  |
|  |  |  |
|  |  |  |
|  |  |  |
|  |  |  |
|  |  |  |
|  |  |  |
|  |  |  |
|  |  |  |
|  |  |  |
|  |  |  |
|  |  |  |
|  |  |  |
|  |  |  |
|  |  |  |
|  |  |  |
|  |  |  |
|  |  |  |
|  |  |  |
|  |  |  |
|  |  |  |
|  |  |  |
|  |  |  |
|  |  |  |
|  |  |  |
|  |  |  |

Please ask for additional pages for recording side effects if needed.

# Medications: Please record any new medicines your child has taken

| Start Date | Stop Date | Dose | Name of Medicine |
| --- | --- | --- | --- |
|  |  |  |  |
|  |  |  |  |
|  |  |  |  |
|  |  |  |  |
|  |  |  |  |
|  |  |  |  |
|  |  |  |  |
|  |  |  |  |
|  |  |  |  |
|  |  |  |  |
|  |  |  |  |
|  |  |  |  |
|  |  |  |  |
|  |  |  |  |
|  |  |  |  |
|  |  |  |  |
|  |  |  |  |
|  |  |  |  |
|  |  |  |  |
|  |  |  |  |
|  |  |  |  |
|  |  |  |  |
|  |  |  |  |
|  |  |  |  |
|  |  |  |  |
|  |  |  |  |
|  |  |  |  |
|  |  |  |  |
|  |  |  |  |
|  |  |  |  |
|  |  |  |  |
|  |  |  |  |
|  |  |  |  |
|  |  |  |  |
|  |  |  |  |
|  |  |  |  |
|  |  |  |  |
|  |  |  |  |
|  |  |  |  |
|  |  |  |  |
|  |  |  |  |
|  |  |  |  |
|  |  |  |  |
|  |  |  |  |

Please ask for additional pages for recording medications if needed.

# Alternating Therapy Period 1 - Week 1 Date:_____________

Total Dose: _________mg/kg/day. Dose is ______ mL twice per day.

Two doses to be administered each day, within 30 minutes of consuming a main meal (e.g. breakfast or dinner). If a dose is missed DO NOT take twice the amount to make up for the missed dose. Please write ‘Dose Missed’ with a reason why.

| Date | Dose 1 Time | Dose 2 Time | Comments/problems with dose |
| --- | --- | --- | --- |
| AP 1 - Week 1 | | | |
| AP 1, Day 1 |  |  |  |
| AP 1, Day 2 |  |  |  |
| AP 1, Day 3 |  |  |  |
| AP 1, Day 4 |  |  |  |
| AP 1, Day 5 |  |  |  |
| AP 1, Day 6 |  |  |  |
| AP 1, Day 7 |  |  |  |

Seizures: Please record seizure types and keep a tally of seizures observed

|  | AP 1, Day 1 | AP 1, Day 2 | AP 1, Day 3 | AP 1, Day 4 | AP 1, Day 5 | AP 1, Day 6 | AP 1, Day 7 |
| --- | --- | --- | --- | --- | --- | --- | --- |
| Seizure Type: | | | | | | | |
| Tally |  |  |  |  |  |  |  |
| Total |  |  |  |  |  |  |  |
| Seizure Type: | | | | | | | |
| Tally |  |  |  |  |  |  |  |
| Total |  |  |  |  |  |  |  |
| Seizure Type: | | | | | | | |
| Tally |  |  |  |  |  |  |  |
| Total |  |  |  |  |  |  |  |
| Seizure Type: | | | | | | | |
| Tally |  |  |  |  |  |  |  |
| Total |  |  |  |  |  |  |  |
| Seizure Type: | | | | | | | |
| Tally |  |  |  |  |  |  |  |
| Total |  |  |  |  |  |  |  |
| Seizure Type: | | | | | | | |
| Tally |  |  |  |  |  |  |  |
| Total |  |  |  |  |  |  |  |

# Alternating Therapy Period 1 - Week 2 Date:_____________

Total Dose: _________mg/kg/day. Dose is ______ mL twice per day.

Two doses to be administered each day, within 30 minutes of consuming a main meal (e.g. breakfast or dinner). If a dose is missed DO NOT take twice the amount to make up for the missed dose. Please write ‘Dose Missed’ with a reason why.

| Date | Dose 1 Time | Dose 2 Time | Comments/problems with dose |
| --- | --- | --- | --- |
| **AP 1 - Week 2** | | | |
| AP 1, Day 8 |  |  |  |
| AP 1, Day 9 |  |  |  |
| AP 1, Day 10 |  |  |  |
| AP 1, Day 11 |  |  |  |
| AP 1, Day 12 |  |  |  |
| AP 1, Day 13 |  |  |  |
| AP 1, Day 14 |  |  |  |

Seizures: Please record seizure types and keep a tally of seizures observed

|  | AP 1, Day 8 | AP 1, Day 9 | AP 1, Day 10 | AP 1, Day 11 | AP 1, Day 12 | AP 1, Day 13 | AP 1, Day 14 |
| --- | --- | --- | --- | --- | --- | --- | --- |
| Seizure Type: | | | | | | | |
| Tally |  |  |  |  |  |  |  |
| Total |  |  |  |  |  |  |  |
| Seizure Type: | | | | | | | |
| Tally |  |  |  |  |  |  |  |
| Total |  |  |  |  |  |  |  |
| Seizure Type: | | | | | | | |
| Tally |  |  |  |  |  |  |  |
| Total |  |  |  |  |  |  |  |
| Seizure Type: | | | | | | | |
| Tally |  |  |  |  |  |  |  |
| Total |  |  |  |  |  |  |  |
| Seizure Type: | | | | | | | |
| Tally |  |  |  |  |  |  |  |
| Total |  |  |  |  |  |  |  |
| Seizure Type: | | | | | | | |
| Tally |  |  |  |  |  |  |  |
| Total |  |  |  |  |  |  |  |

# Alternating Therapy Period 2 - Week 1 Date:_____________

Total Dose: _________mg/kg/day. Dose is ______ mL twice per day.

Two doses to be administered each day, within 30 minutes of consuming a main meal (e.g. breakfast or dinner). If a dose is missed DO NOT take twice the amount to make up for the missed dose. Please write ‘Dose Missed’ with a reason why.

| Date | Dose 1 Time | Dose 2 Time | Comments/problems with dose |
| --- | --- | --- | --- |
| AP 2 - Week 1 | | | |
| AP 2, Day 1 |  |  |  |
| AP 2, Day 2 |  |  |  |
| AP 2, Day 3 |  |  |  |
| AP 2, Day 4 |  |  |  |
| AP 2, Day 5 |  |  |  |
| AP 2, Day 6 |  |  |  |
| AP 2, Day 7 |  |  |  |

Seizures: Please record seizure types and keep a tally of seizures observed

|  | AP 2, Day 1 | AP 2, Day 2 | AP 2, Day 3 | AP 2, Day 4 | AP 2, Day 5 | AP 2, Day 6 | AP 2, Day 7 |
| --- | --- | --- | --- | --- | --- | --- | --- |
| Seizure Type: | | | | | | | |
| Tally |  |  |  |  |  |  |  |
| Total |  |  |  |  |  |  |  |
| Seizure Type: | | | | | | | |
| Tally |  |  |  |  |  |  |  |
| Total |  |  |  |  |  |  |  |
| Seizure Type: | | | | | | | |
| Tally |  |  |  |  |  |  |  |
| Total |  |  |  |  |  |  |  |
| Seizure Type: | | | | | | | |
| Tally |  |  |  |  |  |  |  |
| Total |  |  |  |  |  |  |  |
| Seizure Type: | | | | | | | |
| Tally |  |  |  |  |  |  |  |
| Total |  |  |  |  |  |  |  |
| Seizure Type: | | | | | | | |
| Tally |  |  |  |  |  |  |  |
| Total |  |  |  |  |  |  |  |

# Alternating Therapy Period 2 - Week 2 Date:_____________

Total Dose: _________mg/kg/day. Dose is ______ mL twice per day.

Two doses to be administered each day, within 30 minutes of consuming a main meal (e.g. breakfast or dinner). If a dose is missed DO NOT take twice the amount to make up for the missed dose. Please write ‘Dose Missed’ with a reason why.

| Date | Dose 1 Time | Dose 2 Time | Comments/problems with dose |
| --- | --- | --- | --- |
| **AP 2, Week 2** | | | |
| AP 2, Day 8 |  |  |  |
| AP 2, Day 9 |  |  |  |
| AP 2, Day 10 |  |  |  |
| AP 2, Day 11 |  |  |  |
| AP 2, Day 12 |  |  |  |
| AP 2, Day 13 |  |  |  |
| AP 2, Day 14 |  |  |  |

Seizures: Please record seizure types and keep a tally of seizures observed

|  | AP 2, Day 8 | AP 2, Day 9 | AP 2, Day 10 | AP 2, Day 11 | AP 2, Day 12 | AP 2, Day 13 | AP 2, Day 14 |
| --- | --- | --- | --- | --- | --- | --- | --- |
| Seizure Type: | | | | | | | |
| Tally |  |  |  |  |  |  |  |
| Total |  |  |  |  |  |  |  |
| Seizure Type: | | | | | | | |
| Tally |  |  |  |  |  |  |  |
| Total |  |  |  |  |  |  |  |
| Seizure Type: | | | | | | | |
| Tally |  |  |  |  |  |  |  |
| Total |  |  |  |  |  |  |  |
| Seizure Type: | | | | | | | |
| Tally |  |  |  |  |  |  |  |
| Total |  |  |  |  |  |  |  |
| Seizure Type: | | | | | | | |
| Tally |  |  |  |  |  |  |  |
| Total |  |  |  |  |  |  |  |
| Seizure Type: | | | | | | | |
| Tally |  |  |  |  |  |  |  |
| Total |  |  |  |  |  |  |  |

# Alternating Therapy Period 3 - Week 1 Date:_____________

Total Dose: _________mg/kg/day. Dose is ______ mL twice per day.

Two doses to be administered each day, within 30 minutes of consuming a main meal (e.g. breakfast or dinner). If a dose is missed DO NOT take twice the amount to make up for the missed dose. Please write ‘Dose Missed’ with a reason why.

| Date | Dose 1 Time | Dose 2 Time | Comments/problems with dose |
| --- | --- | --- | --- |
| AP 3 - Week 1 | | | |
| AP 3, Day 1 |  |  |  |
| AP 3, Day 2 |  |  |  |
| AP 3, Day 3 |  |  |  |
| AP 3, Day 4 |  |  |  |
| AP 3, Day 5 |  |  |  |
| AP 3, Day 6 |  |  |  |
| AP 3, Day 7 |  |  |  |

Seizures: Please record seizure types and keep a tally of seizures observed

|  | AP 3, Day 1 | AP 3, Day 2 | AP 3, Day 3 | AP 3, Day 4 | AP 3, Day 5 | AP 3, Day 6 | AP 3, Day 7 |
| --- | --- | --- | --- | --- | --- | --- | --- |
| Seizure Type: | | | | | | | |
| Tally |  |  |  |  |  |  |  |
| Total |  |  |  |  |  |  |  |
| Seizure Type: | | | | | | | |
| Tally |  |  |  |  |  |  |  |
| Total |  |  |  |  |  |  |  |
| Seizure Type: | | | | | | | |
| Tally |  |  |  |  |  |  |  |
| Total |  |  |  |  |  |  |  |
| Seizure Type: | | | | | | | |
| Tally |  |  |  |  |  |  |  |
| Total |  |  |  |  |  |  |  |
| Seizure Type: | | | | | | | |
| Tally |  |  |  |  |  |  |  |
| Total |  |  |  |  |  |  |  |
| Seizure Type: | | | | | | | |
| Tally |  |  |  |  |  |  |  |
| Total |  |  |  |  |  |  |  |

# Alternating Therapy Period 3 - Week 2 Date:_____________

Total Dose: _________mg/kg/day. Dose is ______ mL twice per day.

Two doses to be administered each day, within 30 minutes of consuming a main meal (e.g. breakfast or dinner). If a dose is missed DO NOT take twice the amount to make up for the missed dose. Please write ‘Dose Missed’ with a reason why.

| Date | Dose 1 Time | Dose 2 Time | Comments/problems with dose |
| --- | --- | --- | --- |
| **AP 3 - Week 2** | | | |
| **AP 3, Day 8** |  |  |  |
| **AP 3, Day 9** |  |  |  |
| **AP 3, Day 10** |  |  |  |
| **AP 3, Day 11** |  |  |  |
| **AP 3, Day 12** |  |  |  |
| **AP 3, Day 13** |  |  |  |
| **AP 3, Day 14** |  |  |  |

Seizures: Please record seizure types and keep a tally of seizures observed

|  | AP 3, Day 8 | AP 3, Day 9 | AP 3, Day 10 | AP 3, Day 11 | AP 3, Day 12 | AP 3, Day 13 | AP 3, Day 14 |
| --- | --- | --- | --- | --- | --- | --- | --- |
| Seizure Type: | | | | | | | |
| Tally |  |  |  |  |  |  |  |
| Total |  |  |  |  |  |  |  |
| Seizure Type: | | | | | | | |
| Tally |  |  |  |  |  |  |  |
| Total |  |  |  |  |  |  |  |
| Seizure Type: | | | | | | | |
| Tally |  |  |  |  |  |  |  |
| Total |  |  |  |  |  |  |  |
| Seizure Type: | | | | | | | |
| Tally |  |  |  |  |  |  |  |
| Total |  |  |  |  |  |  |  |
| Seizure Type: | | | | | | | |
| Tally |  |  |  |  |  |  |  |
| Total |  |  |  |  |  |  |  |
| Seizure Type: | | | | | | | |
| Tally |  |  |  |  |  |  |  |
| Total |  |  |  |  |  |  |  |

# Alternating Therapy Period 4 - Week 1 Date:_____________

Total Dose: _________mg/kg/day. Dose is ______ mL twice per day.

Two doses to be administered each day, within 30 minutes of consuming a main meal (e.g. breakfast or dinner). If a dose is missed DO NOT take twice the amount to make up for the missed dose. Please write ‘Dose Missed’ with a reason why.

| Date | Dose 1 Time | Dose 2 Time | Comments/problems with dose |
| --- | --- | --- | --- |
| AP 4- Week 1 | | | |
| AP 4, Day 1 |  |  |  |
| AP 4, Day 2 |  |  |  |
| AP 4, Day 3 |  |  |  |
| AP 4, Day 4 |  |  |  |
| AP 4, Day 5 |  |  |  |
| AP 4, Day 6 |  |  |  |
| AP 4, Day 7 |  |  |  |

Seizures: Please record seizure types and keep a tally of seizures observed

|  | AP 4, Day 1 | AP 4, Day 2 | AP 4, Day 3 | AP 4, Day 4 | AP 4, Day 5 | AP 4, Day 6 | AP 4, Day 7 |
| --- | --- | --- | --- | --- | --- | --- | --- |
| Seizure Type: | | | | | | | |
| Tally |  |  |  |  |  |  |  |
| Total |  |  |  |  |  |  |  |
| Seizure Type: | | | | | | | |
| Tally |  |  |  |  |  |  |  |
| Total |  |  |  |  |  |  |  |
| Seizure Type: | | | | | | | |
| Tally |  |  |  |  |  |  |  |
| Total |  |  |  |  |  |  |  |
| Seizure Type: | | | | | | | |
| Tally |  |  |  |  |  |  |  |
| Total |  |  |  |  |  |  |  |
| Seizure Type: | | | | | | | |
| Tally |  |  |  |  |  |  |  |
| Total |  |  |  |  |  |  |  |
| Seizure Type: | | | | | | | |
| Tally |  |  |  |  |  |  |  |
| Total |  |  |  |  |  |  |  |

# Alternating Therapy Period 4 - Week 2 Date:_____________

Total Dose: _________mg/kg/day. Dose is ______ mL twice per day.

Two doses to be administered each day, within 30 minutes of consuming a main meal (e.g. breakfast or dinner). If a dose is missed DO NOT take twice the amount to make up for the missed dose. Please write ‘Dose Missed’ with a reason why.

| Date | Dose 1 Time | Dose 2 Time | Comments/problems with dose |
| --- | --- | --- | --- |
| **AP 4 -Week 2** | | | |
| AP 4, Day 8 |  |  |  |
| AP 4, Day 9 |  |  |  |
| AP 4, Day 10 |  |  |  |
| AP 4, Day 11 |  |  |  |
| AP 4, Day 12 |  |  |  |
| AP 4, Day 13 |  |  |  |
| AP 4, Day 14 |  |  |  |

Seizures: Please record seizure types and keep a tally of seizures observed

|  | AP 4, Day 8 | AP 4, Day 9 | AP 4, Day 10 | AP 4, Day 11 | AP 4, Day 12 | AP 4, Day 13 | AP 4, Day 14 |
| --- | --- | --- | --- | --- | --- | --- | --- |
| Seizure Type: | | | | | | | |
| Tally |  |  |  |  |  |  |  |
| Total |  |  |  |  |  |  |  |
| Seizure Type: | | | | | | | |
| Tally |  |  |  |  |  |  |  |
| Total |  |  |  |  |  |  |  |
| Seizure Type: | | | | | | | |
| Tally |  |  |  |  |  |  |  |
| Total |  |  |  |  |  |  |  |
| Seizure Type: | | | | | | | |
| Tally |  |  |  |  |  |  |  |
| Total |  |  |  |  |  |  |  |
| Seizure Type: | | | | | | | |
| Tally |  |  |  |  |  |  |  |
| Total |  |  |  |  |  |  |  |
| Seizure Type: | | | | | | | |
| Tally |  |  |  |  |  |  |  |
| Total |  |  |  |  |  |  |  |

# Alternating Therapy Period 5 - Week 1 Date:_____________

Total Dose: _________mg/kg/day. Dose is ______ mL twice per day.

Two doses to be administered each day, within 30 minutes of consuming a main meal (e.g. breakfast or dinner). If a dose is missed DO NOT take twice the amount to make up for the missed dose. Please write ‘Dose Missed’ with a reason why.

| Date | Dose 1 Time | Dose 2 Time | Comments/problems with dose |
| --- | --- | --- | --- |
| AP 5 - Week 1 | | | |
| AP 5, Day 1 |  |  |  |
| AP 5, Day 2 |  |  |  |
| AP 5, Day 3 |  |  |  |
| AP 5, Day 4 |  |  |  |
| AP 5, Day 5 |  |  |  |
| AP 5, Day 6 |  |  |  |
| AP 5, Day 7 |  |  |  |

Seizures: Please record seizure types and keep a tally of seizures observed

|  | AP 5, Day 1 | AP 5, Day 2 | AP 5, Day 3 | AP 5, Day 4 | AP 5, Day 5 | AP 5, Day 6 | AP 5, Day 7 |
| --- | --- | --- | --- | --- | --- | --- | --- |
| Seizure Type: | | | | | | | |
| Tally |  |  |  |  |  |  |  |
| Total |  |  |  |  |  |  |  |
| Seizure Type: | | | | | | | |
| Tally |  |  |  |  |  |  |  |
| Total |  |  |  |  |  |  |  |
| Seizure Type: | | | | | | | |
| Tally |  |  |  |  |  |  |  |
| Total |  |  |  |  |  |  |  |
| Seizure Type: | | | | | | | |
| Tally |  |  |  |  |  |  |  |
| Total |  |  |  |  |  |  |  |
| Seizure Type: | | | | | | | |
| Tally |  |  |  |  |  |  |  |
| Total |  |  |  |  |  |  |  |
| Seizure Type: | | | | | | | |
| Tally |  |  |  |  |  |  |  |
| Total |  |  |  |  |  |  |  |

# Alternating Therapy Period 5 - Week 2 Date:_____________

Total Dose: _________mg/kg/day. Dose is ______ mL twice per day.

Two doses to be administered each day, within 30 minutes of consuming a main meal (e.g. breakfast or dinner). If a dose is missed DO NOT take twice the amount to make up for the missed dose. Please write ‘Dose Missed’ with a reason why.

| Date | Dose 1 Time | Dose 2 Time | Comments/problems with dose |
| --- | --- | --- | --- |
| **AP 5 - Week 2** | | | |
| AP 5, Day 8 |  |  |  |
| AP 5, Day 9 |  |  |  |
| AP 5, Day 10 |  |  |  |
| AP 5, Day 11 |  |  |  |
| AP 5, Day 12 |  |  |  |
| AP 5, Day 13 |  |  |  |
| AP 5, Day 14 |  |  |  |

Seizures: Please record seizure types and keep a tally of seizures observed

|  | AP 5, Day 8 | AP 5, Day 9 | AP 5, Day 10 | AP 5, Day 11 | AP 5, Day 12 | AP 5, Day 13 | AP 5, Day 14 |
| --- | --- | --- | --- | --- | --- | --- | --- |
| Seizure Type: | | | | | | | |
| Tally |  |  |  |  |  |  |  |
| Total |  |  |  |  |  |  |  |
| Seizure Type: | | | | | | | |
| Tally |  |  |  |  |  |  |  |
| Total |  |  |  |  |  |  |  |
| Seizure Type: | | | | | | | |
| Tally |  |  |  |  |  |  |  |
| Total |  |  |  |  |  |  |  |
| Seizure Type: | | | | | | | |
| Tally |  |  |  |  |  |  |  |
| Total |  |  |  |  |  |  |  |
| Seizure Type: | | | | | | | |
| Tally |  |  |  |  |  |  |  |
| Total |  |  |  |  |  |  |  |
| Seizure Type: | | | | | | | |
| Tally |  |  |  |  |  |  |  |
| Total |  |  |  |  |  |  |  |

# Alternating Therapy Period 6 - Week 1 Date:_____________

Total Dose: _________mg/kg/day. Dose is ______ mL twice per day.

Two doses to be administered each day, within 30 minutes of consuming a main meal (e.g. breakfast or dinner). If a dose is missed DO NOT take twice the amount to make up for the missed dose. Please write ‘Dose Missed’ with a reason why.

| Date | Dose 1 Time | Dose 2 Time | Comments/problems with dose |
| --- | --- | --- | --- |
| AP 6 - Week 1 | | | |
| AP 6, Day 1 |  |  |  |
| AP 6, Day 2 |  |  |  |
| AP 6, Day 3 |  |  |  |
| AP 6, Day 4 |  |  |  |
| AP 6, Day 5 |  |  |  |
| AP 6, Day 6 |  |  |  |
| AP 6, Day 7 |  |  |  |

Seizures: Please record seizure types and keep a tally of seizures observed

|  | AP 6, Day 1 | AP 6, Day 2 | AP 6, Day 3 | AP 6, Day 4 | AP 6, Day 5 | AP 6, Day 6 | AP 6, Day 7 |
| --- | --- | --- | --- | --- | --- | --- | --- |
| Seizure Type: | | | | | | | |
| Tally |  |  |  |  |  |  |  |
| Total |  |  |  |  |  |  |  |
| Seizure Type: | | | | | | | |
| Tally |  |  |  |  |  |  |  |
| Total |  |  |  |  |  |  |  |
| Seizure Type: | | | | | | | |
| Tally |  |  |  |  |  |  |  |
| Total |  |  |  |  |  |  |  |
| Seizure Type: | | | | | | | |
| Tally |  |  |  |  |  |  |  |
| Total |  |  |  |  |  |  |  |
| Seizure Type: | | | | | | | |
| Tally |  |  |  |  |  |  |  |
| Total |  |  |  |  |  |  |  |
| Seizure Type: | | | | | | | |
| Tally |  |  |  |  |  |  |  |
| Total |  |  |  |  |  |  |  |

# Alternating Therapy Period 6 - Week 2 Date:_____________

Total Dose: _________mg/kg/day. Dose is ______ mL twice per day.

Two doses to be administered each day, within 30 minutes of consuming a main meal (e.g. breakfast or dinner). If a dose is missed DO NOT take twice the amount to make up for the missed dose. Please write ‘Dose Missed’ with a reason why.

| Date | Dose 1 Time | Dose 2 Time | Comments/problems with dose |
| --- | --- | --- | --- |
| **AP 6 - Week 2** | | | |
| AP 6, Day 8 |  |  |  |
| AP 6, Day 9 |  |  |  |
| AP 6, Day 10 |  |  |  |
| AP 6, Day 11 |  |  |  |
| AP 6, Day 12 |  |  |  |
| AP 6, Day 13 |  |  |  |
| AP 6, Day 14 |  |  |  |

Seizures: Please record seizure types and keep a tally of seizures observed

|  | AP 6, Day 8 | AP 6, Day 9 | AP 6, Day 10 | AP 6, Day 11 | AP 6, Day 12 | AP 6, Day 13 | AP 6, Day 14 |
| --- | --- | --- | --- | --- | --- | --- | --- |
| Seizure Type: | | | | | | | |
| Tally |  |  |  |  |  |  |  |
| Total |  |  |  |  |  |  |  |
| Seizure Type: | | | | | | | |
| Tally |  |  |  |  |  |  |  |
| Total |  |  |  |  |  |  |  |
| Seizure Type: | | | | | | | |
| Tally |  |  |  |  |  |  |  |
| Total |  |  |  |  |  |  |  |
| Seizure Type: | | | | | | | |
| Tally |  |  |  |  |  |  |  |
| Total |  |  |  |  |  |  |  |
| Seizure Type: | | | | | | | |
| Tally |  |  |  |  |  |  |  |
| Total |  |  |  |  |  |  |  |
| Seizure Type: | | | | | | | |
| Tally |  |  |  |  |  |  |  |
| Total |  |  |  |  |  |  |  |

# Alternating Therapy Period 7 - Week 1 Date:_____________

Total Dose: _________mg/kg/day. Dose is ______ mL twice per day.

Two doses to be administered each day, within 30 minutes of consuming a main meal (e.g. breakfast or dinner). If a dose is missed DO NOT take twice the amount to make up for the missed dose. Please write ‘Dose Missed’ with a reason why.

| Date | Dose 1 Time | Dose 2 Time | Comments/problems with dose |
| --- | --- | --- | --- |
| AP 7 - Week 1 | | | |
| AP 7, Day 1 |  |  |  |
| AP 7, Day 2 |  |  |  |
| AP 7, Day 3 |  |  |  |
| AP 7, Day 4 |  |  |  |
| AP 7, Day 5 |  |  |  |
| AP 7, Day 6 |  |  |  |
| AP 7, Day 7 |  |  |  |

Seizures: Please record seizure types and keep a tally of seizures observed

|  | AP 7, Day 1 | AP 7, Day 2 | AP 7, Day 3 | AP 7, Day 4 | AP 7, Day 5 | AP 7, Day 6 | AP 7, Day 7 |
| --- | --- | --- | --- | --- | --- | --- | --- |
| Seizure Type: | | | | | | | |
| Tally |  |  |  |  |  |  |  |
| Total |  |  |  |  |  |  |  |
| Seizure Type: | | | | | | | |
| Tally |  |  |  |  |  |  |  |
| Total |  |  |  |  |  |  |  |
| Seizure Type: | | | | | | | |
| Tally |  |  |  |  |  |  |  |
| Total |  |  |  |  |  |  |  |
| Seizure Type: | | | | | | | |
| Tally |  |  |  |  |  |  |  |
| Total |  |  |  |  |  |  |  |
| Seizure Type: | | | | | | | |
| Tally |  |  |  |  |  |  |  |
| Total |  |  |  |  |  |  |  |
| Seizure Type: | | | | | | | |
| Tally |  |  |  |  |  |  |  |
| Total |  |  |  |  |  |  |  |

# Alternating Therapy Period 7 - Week 2 Date:_____________

Total Dose: _________mg/kg/day. Dose is ______ mL twice per day.

Two doses to be administered each day, within 30 minutes of consuming a main meal (e.g. breakfast or dinner). If a dose is missed DO NOT take twice the amount to make up for the missed dose. Please write ‘Dose Missed’ with a reason why.

| Date | Dose 1 Time | Dose 2 Time | Comments/problems with dose |
| --- | --- | --- | --- |
| **AP 7 - Week 2** | | | |
| AP 7, Day 8 |  |  |  |
| AP 7, Day 9 |  |  |  |
| AP 7, Day 10 |  |  |  |
| AP 7, Day 11 |  |  |  |
| AP 7, Day 12 |  |  |  |
| AP 7, Day 13 |  |  |  |
| AP 7, Day 14 |  |  |  |

Seizures: Please record seizure types and keep a tally of seizures observed

|  | AP 7, Day 8 | AP 7, Day 9 | AP 7, Day 10 | AP 7, Day 11 | AP 7, Day 12 | AP 7, Day 13 | AP 7, Day 14 |
| --- | --- | --- | --- | --- | --- | --- | --- |
| Seizure Type: | | | | | | | |
| Tally |  |  |  |  |  |  |  |
| Total |  |  |  |  |  |  |  |
| Seizure Type: | | | | | | | |
| Tally |  |  |  |  |  |  |  |
| Total |  |  |  |  |  |  |  |
| Seizure Type: | | | | | | | |
| Tally |  |  |  |  |  |  |  |
| Total |  |  |  |  |  |  |  |
| Seizure Type: | | | | | | | |
| Tally |  |  |  |  |  |  |  |
| Total |  |  |  |  |  |  |  |
| Seizure Type: | | | | | | | |
| Tally |  |  |  |  |  |  |  |
| Total |  |  |  |  |  |  |  |
| Seizure Type: | | | | | | | |
| Tally |  |  |  |  |  |  |  |
| Total |  |  |  |  |  |  |  |

# Alternating Therapy Period 8 - Week 1 Date:_____________

Total Dose: _________mg/kg/day. Dose is ______ mL twice per day.

Two doses to be administered each day, within 30 minutes of consuming a main meal (e.g. breakfast or dinner). If a dose is missed DO NOT take twice the amount to make up for the missed dose. Please write ‘Dose Missed’ with a reason why.

| Date | Dose 1 Time | Dose 2 Time | Comments/problems with dose |
| --- | --- | --- | --- |
| AP 8 - Week 1 | | | |
| AP 8, Day 1 |  |  |  |
| AP 8, Day 2 |  |  |  |
| AP 8, Day 3 |  |  |  |
| AP 8, Day 4 |  |  |  |
| AP 8, Day 5 |  |  |  |
| AP 8, Day 6 |  |  |  |
| AP 8, Day 7 |  |  |  |

Seizures: Please record seizure types and keep a tally of seizures observed

|  | AP 8, Day 1 | AP 8, Day 2 | AP 8, Day 3 | AP 8, Day 4 | AP 8, Day 5 | AP 8, Day 6 | AP 8, Day 7 |
| --- | --- | --- | --- | --- | --- | --- | --- |
| Seizure Type: | | | | | | | |
| Tally |  |  |  |  |  |  |  |
| Total |  |  |  |  |  |  |  |
| Seizure Type: | | | | | | | |
| Tally |  |  |  |  |  |  |  |
| Total |  |  |  |  |  |  |  |
| Seizure Type: | | | | | | | |
| Tally |  |  |  |  |  |  |  |
| Total |  |  |  |  |  |  |  |
| Seizure Type: | | | | | | | |
| Tally |  |  |  |  |  |  |  |
| Total |  |  |  |  |  |  |  |
| Seizure Type: | | | | | | | |
| Tally |  |  |  |  |  |  |  |
| Total |  |  |  |  |  |  |  |
| Seizure Type: | | | | | | | |
| Tally |  |  |  |  |  |  |  |
| Total |  |  |  |  |  |  |  |

# Alternating Therapy Period 8 - Week 2 Date:_____________

Total Dose: _________mg/kg/day. Dose is ______ mL twice per day.

Two doses to be administered each day, within 30 minutes of consuming a main meal (e.g. breakfast or dinner). If a dose is missed DO NOT take twice the amount to make up for the missed dose. Please write ‘Dose Missed’ with a reason why.

| Date | Dose 1 Time | Dose 2 Time | Comments/problems with dose |
| --- | --- | --- | --- |
| **AP 8 - Week 2** | | | |
| AP 8, Day 8 |  |  |  |
| AP 8, Day 9 |  |  |  |
| AP 8, Day 10 |  |  |  |
| AP 8, Day 11 |  |  |  |
| AP 8, Day 12 |  |  |  |
| AP 8, Day 13 |  |  |  |
| AP 8, Day 14 |  |  |  |

Seizures: Please record seizure types and keep a tally of seizures observed

|  | AP 8, Day 8 | AP 8, Day 9 | AP 8, Day 10 | AP 8, Day 11 | AP 8, Day 12 | AP 8, Day 13 | AP 8, Day 14 |
| --- | --- | --- | --- | --- | --- | --- | --- |
| Seizure Type: | | | | | | | |
| Tally |  |  |  |  |  |  |  |
| Total |  |  |  |  |  |  |  |
| Seizure Type: | | | | | | | |
| Tally |  |  |  |  |  |  |  |
| Total |  |  |  |  |  |  |  |
| Seizure Type: | | | | | | | |
| Tally |  |  |  |  |  |  |  |
| Total |  |  |  |  |  |  |  |
| Seizure Type: | | | | | | | |
| Tally |  |  |  |  |  |  |  |
| Total |  |  |  |  |  |  |  |
| Seizure Type: | | | | | | | |
| Tally |  |  |  |  |  |  |  |
| Total |  |  |  |  |  |  |  |
| Seizure Type: | | | | | | | |
| Tally |  |  |  |  |  |  |  |
| Total |  |  |  |  |  |  |  |

# Additional Page, Use only if Needed Date________________

Total Dose: _________mg/kg/day. Dose is ______ mL twice per day.

Two doses to be administered each day, within 30 minutes of consuming a main meal (e.g. breakfast or dinner). If a dose is missed DO NOT take twice the amount to make up for the missed dose. Please write ‘Dose Missed’ with a reason why.

| Date | Dose 1 Time | Dose 2 Time | Comments/problems with dose |
| --- | --- | --- | --- |
|  |  |  |  |
|  |  |  |  |
|  |  |  |  |
|  |  |  |  |
|  |  |  |  |
|  |  |  |  |
|  |  |  |  |

Seizures: Please record seizure types and keep a tally of seizures observed

| DATE |  |  |  |  |  |  |  |
| --- | --- | --- | --- | --- | --- | --- | --- |
| Seizure Type: | | | | | | | |
| Tally |  |  |  |  |  |  |  |
| Total |  |  |  |  |  |  |  |
| Seizure Type: | | | | | | | |
| Tally |  |  |  |  |  |  |  |
| Total |  |  |  |  |  |  |  |
| Seizure Type: | | | | | | | |
| Tally |  |  |  |  |  |  |  |
| Total |  |  |  |  |  |  |  |
| Seizure Type: | | | | | | | |
| Tally |  |  |  |  |  |  |  |
| Total |  |  |  |  |  |  |  |
| Seizure Type: | | | | | | | |
| Tally |  |  |  |  |  |  |  |
| Total |  |  |  |  |  |  |  |
| Seizure Type: | | | | | | | |
| Tally |  |  |  |  |  |  |  |
| Total |  |  |  |  |  |  |  |

Please ask for additional pages if needed.

**Appendix 6. Patient Seizure Diary Alternating Therapy Period (4 week treatment period)**

**Disclaimer:**

These materials are provided for information and education purposes only. Information about a therapy, service, product or treatment does not in any way endorse or support such therapy, service, product or treatment, whether for any individual patient, generally or otherwise.

These materials were developed as a general informational tool to support clinical practice by qualified medical professionals considering medicinal cannabis treatment in patients with epilepsy. They are not a substitute for comprehensive individual clinical assessments and are not intended to constitute a comprehensive guide for assessing whether treatment with medicinal cannabis is or will be beneficial for any individual patient or any particular class of patients, or regarding any other aspect of treatment of patients with medicinal cannabis.

Decisions regarding treatment with medicinal cannabis, and any determination regarding whether treatment with medicinal cannabis is suitable in the circumstances, are the sole responsibility of the treating medical professional, exercising their own clinical judgment and taking into account all of the circumstances and medical history of the patient/s. For the avoidance of doubt, these materials were not developed for use by patients, and patients are solely responsible for seeking medical advice in relation to any treatment with medicinal cannabis.

The State of Victoria and the Department of Health and Human Services shall not bear any liability to you or any third party for any loss or damage which may result from your use of or reliance on any information contained in these materials, including but not limited to the use or application of any information contained in these materials, generation of data by these materials, analysis of the data generated by these materials, and/or any determinations made regarding treatment with medicinal cannabis for an individual patient, a class of patients or generally.

**PATIENT DIARY – ALTERNATING THERAPY PERIOD**

**4 WEEK TREATMENT PERIOD**

**Contact Details at [Site]: [Name and Phone] Patient Name:**

# *At each visit, you must remember to return all used and unused medication bottles to the pharmacy. Pharmacy cannot dispense more without receiving the old bottles.*

- *If you’re admitted to hospital, remember to bring your own supply of medication from this Single Patient Therapy Plan along with this patient diary and your Wallet Card.*

| **ALTERNATING THERAPY PERIOD VISITS (6 TREATMENT PERIODS OF 4 WEEKS DURATION)** | |
| --- | --- |
| **VISIT 4**  (_ _/_ _/_ _) | - **Please remember to bring this patient diary to the appointment**. - **Please remember to return all used and unused medication to the pharmacy.** - Meet with your Clinician to review the diary - Collect medication (Cannabidiol/Placebo) from Pharmacy - Next Appointment in 4 weeks: Date & Time: ________________________________ |
| **VISIT 5**  (_ _/_ _/_ _) | - **Please remember to bring this patient diary to the appointment**. - **Please remember to return all used and unused medication to the pharmacy.** - Meet with your Clinician to review the diary - Collect medication (Cannabidiol/Placebo) from Pharmacy - Next Appointment in 4 weeks: Date & Time: ________________________________ |
| **VISIT 6**  (_ _/_ _/_ _) | - **Please remember to bring this patient diary to the appointment**. - **Please remember to return all used and unused medication to the pharmacy.** - Meet with your Clinician to review the diary - Collect medication (Cannabidiol/Placebo) from Pharmacy - Next Appointment in 4 weeks: Date & Time: ________________________________ |
| **VISIT 7**  (_ _/_ _/_ _) | - **Please remember to bring this patient diary to the appointment**. - **Please remember to return all used and unused medication to the pharmacy.** - Meet with your Clinician to review the diary - Collect medication (Cannabidiol/Placebo) from Pharmacy - Collect Blood Test Request Form and have bloods taken prior to your next appointment - Next Appointment in 4 weeks: Date & Time: ________________________________ |
| **VISIT 8**  (_ _/_ _/_ _) | - **Please remember to bring this patient diary to the appointment**. - **Please remember to return all used and unused medication to the pharmacy.** - Meet with your Clinician to review the diary - Collect medication (Cannabidiol/Placebo) from Pharmacy - Next Appointment in 4 weeks: Date & Time: ________________________________ |

| **VISIT 9**  (_ _/_ _/_ _) | - **Please remember to bring this patient diary to the appointment**. - **Please remember to return all used and unused medication to the pharmacy.** - Meet with your Clinician to review the diary - Collect medication (Cannabidiol/Placebo) from Pharmacy - Collect Blood Test Request Form and have bloods taken prior to your next appointment - Next Appointment in 4 weeks: Date & Time: ________________________________ |
| --- | --- |
| **VISIT 10**  (_ _/_ _/_ _) | - **Please remember to bring this patient diary to the appointment**. - **Please remember to return all used and unused medication to the pharmacy.** - Meet with your Clinician to review the diary - Determine with Clinician if optional treatment periods are needed: YES or NO   If YES – collect medication (Cannabidiol/Placebo) from Pharmacy   - Next Appointment in 4 weeks Date & Time: __________________________________________ |

| **ALTERNATING THERAPY PERIODS 7 & 8 (USE ONLY IF YOUR CLINICIAN SAYS THEY ARE NEEDED)** | |
| --- | --- |
| **VISIT DATE:**  (_ _/_ _/_ _)  **For Alternating Therapy Period 7** | - **Please remember to bring this patient diary to the appointment**. - **Please remember to return all used and unused medication to the pharmacy.** - Meet with your Clinician to review the diary - Collect medication (Cannabidiol/Placebo) from Pharmacy   Next Appointment in 4 weeks: Date & Time: ________________________________ |
| **VISIT DATE:**  (_ _/_ _/_ _)  **For Alternating Therapy Period 8** | - **Please remember to bring this patient diary to the appointment**. - **Please remember to return all used and unused medication to the pharmacy.** - Meet with your Clinician to review the diary - Collect medication (Cannabidiol/Placebo) from Pharmacy - Next Appointment in 4 weeks: Date & Time: ________________________________ |

| **RESULTS VISIT** | |
| --- | --- |
| **VISIT 11**  (_ _/_ _/_ _) | - **Please remember to bring this patient diary to the appointment**. - **Please remember to return all used and unused medication to the pharmacy.** - Meet with your Clinician to review the diary - Collect medication (Cannabidiol) from Pharmacy, if required |

| **UNSCHEDULED VISITS** | |
| --- | --- |
|  | - **Please remember to bring this patient diary to the appointment**. - **Please remember to return all used and unused medication to the pharmacy.** - Meet with your Clinician to discuss the reason for the visit - Collect medication (Cannabidiol) from Pharmacy, if required |

# Side Effects: Please record all side effects observed

| Start Date | Stop Date | Describe Side effect |
| --- | --- | --- |
|  |  |  |
|  |  |  |
|  |  |  |
|  |  |  |
|  |  |  |
|  |  |  |
|  |  |  |
|  |  |  |
|  |  |  |
|  |  |  |
|  |  |  |
|  |  |  |
|  |  |  |
|  |  |  |
|  |  |  |
|  |  |  |
|  |  |  |
|  |  |  |
|  |  |  |
|  |  |  |
|  |  |  |
|  |  |  |
|  |  |  |
|  |  |  |
|  |  |  |
|  |  |  |
|  |  |  |
|  |  |  |
|  |  |  |
|  |  |  |
|  |  |  |
|  |  |  |
|  |  |  |
|  |  |  |
|  |  |  |
|  |  |  |
|  |  |  |
|  |  |  |
|  |  |  |
|  |  |  |
|  |  |  |
|  |  |  |
|  |  |  |
|  |  |  |

Please ask for additional pages for recording side effects if needed.

# Medications: Please record any new medicines your child has taken

| Start Date | Stop Date | Dose | Name of Medicine |
| --- | --- | --- | --- |
|  |  |  |  |
|  |  |  |  |
|  |  |  |  |
|  |  |  |  |
|  |  |  |  |
|  |  |  |  |
|  |  |  |  |
|  |  |  |  |
|  |  |  |  |
|  |  |  |  |
|  |  |  |  |
|  |  |  |  |
|  |  |  |  |
|  |  |  |  |
|  |  |  |  |
|  |  |  |  |
|  |  |  |  |
|  |  |  |  |
|  |  |  |  |
|  |  |  |  |
|  |  |  |  |
|  |  |  |  |
|  |  |  |  |
|  |  |  |  |
|  |  |  |  |
|  |  |  |  |
|  |  |  |  |
|  |  |  |  |
|  |  |  |  |
|  |  |  |  |
|  |  |  |  |
|  |  |  |  |
|  |  |  |  |
|  |  |  |  |
|  |  |  |  |
|  |  |  |  |
|  |  |  |  |
|  |  |  |  |
|  |  |  |  |
|  |  |  |  |
|  |  |  |  |
|  |  |  |  |
|  |  |  |  |
|  |  |  |  |

Please ask for additional pages for recording medications if needed.

# Alternating Therapy Period 1 - Week 1 Date:_____________

Total Dose: _________mg/kg/day. Dose is ______ mL twice per day.

Two doses to be administered each day, within 30 minutes of consuming a main meal (e.g. breakfast or dinner). If a dose is missed DO NOT take twice the amount to make up for the missed dose. Please write ‘Dose Missed’ with a reason why.

| Date | Dose 1 Time | Dose 2 Time | Comments/problems with dose |
| --- | --- | --- | --- |
| AP 1 - Week 1 | | | |
| AP 1, Day 1 |  |  |  |
| AP 1, Day 2 |  |  |  |
| AP 1, Day 3 |  |  |  |
| AP 1, Day 4 |  |  |  |
| AP 1, Day 5 |  |  |  |
| AP 1, Day 6 |  |  |  |
| AP 1, Day 7 |  |  |  |

Seizures: Please record seizure types and keep a tally of seizures observed

|  | AP 1, Day 1 | AP 1, Day 2 | AP 1, Day 3 | AP 1, Day 4 | AP 1, Day 5 | AP 1, Day 6 | AP 1, Day 7 |
| --- | --- | --- | --- | --- | --- | --- | --- |
| Seizure Type: | | | | | | | |
| Tally |  |  |  |  |  |  |  |
| Total |  |  |  |  |  |  |  |
| Seizure Type: | | | | | | | |
| Tally |  |  |  |  |  |  |  |
| Total |  |  |  |  |  |  |  |
| Seizure Type: | | | | | | | |
| Tally |  |  |  |  |  |  |  |
| Total |  |  |  |  |  |  |  |
| Seizure Type: | | | | | | | |
| Tally |  |  |  |  |  |  |  |
| Total |  |  |  |  |  |  |  |
| Seizure Type: | | | | | | | |
| Tally |  |  |  |  |  |  |  |
| Total |  |  |  |  |  |  |  |
| Seizure Type: | | | | | | | |
| Tally |  |  |  |  |  |  |  |
| Total |  |  |  |  |  |  |  |

# Alternating Therapy Period 1 - Week 2 Date:_____________

Total Dose: _________mg/kg/day. Dose is ______ mL twice per day.

Two doses to be administered each day, within 30 minutes of consuming a main meal (e.g. breakfast or dinner). If a dose is missed DO NOT take twice the amount to make up for the missed dose. Please write ‘Dose Missed’ with a reason why.

| Date | Dose 1 Time | Dose 2 Time | Comments/problems with dose |
| --- | --- | --- | --- |
| **AP 1 - Week 2** | | | |
| AP 1, Day 8 |  |  |  |
| AP 1, Day 9 |  |  |  |
| AP 1, Day 10 |  |  |  |
| AP 1, Day 11 |  |  |  |
| AP 1, Day 12 |  |  |  |
| AP 1, Day 13 |  |  |  |
| AP 1, Day 14 |  |  |  |

Seizures: Please record seizure types and keep a tally of seizures observed

|  | AP 1, Day 8 | AP 1, Day 9 | AP 1, Day 10 | AP 1, Day 11 | AP 1, Day 12 | AP 1, Day 13 | AP 1, Day 14 |
| --- | --- | --- | --- | --- | --- | --- | --- |
| Seizure Type: | | | | | | | |
| Tally |  |  |  |  |  |  |  |
| Total |  |  |  |  |  |  |  |
| Seizure Type: | | | | | | | |
| Tally |  |  |  |  |  |  |  |
| Total |  |  |  |  |  |  |  |
| Seizure Type: | | | | | | | |
| Tally |  |  |  |  |  |  |  |
| Total |  |  |  |  |  |  |  |
| Seizure Type: | | | | | | | |
| Tally |  |  |  |  |  |  |  |
| Total |  |  |  |  |  |  |  |
| Seizure Type: | | | | | | | |
| Tally |  |  |  |  |  |  |  |
| Total |  |  |  |  |  |  |  |
| Seizure Type: | | | | | | | |
| Tally |  |  |  |  |  |  |  |
| Total |  |  |  |  |  |  |  |

# Alternating Therapy Period 1 - Week 3 Date:_____________

Total Dose: _________mg/kg/day. Dose is ______ mL twice per day.

Two doses to be administered each day, within 30 minutes of consuming a main meal (e.g. breakfast or dinner). If a dose is missed DO NOT take twice the amount to make up for the missed dose. Please write ‘Dose Missed’ with a reason why.

| Date | Dose 1 Time | Dose 2 Time | Comments/problems with dose |
| --- | --- | --- | --- |
| **AP 1 - Week 3** | | | |
| AP 1, Day 15 |  |  |  |
| AP 1, Day 16 |  |  |  |
| AP 1, Day 17 |  |  |  |
| AP 1, Day 18 |  |  |  |
| AP 1, Day 19 |  |  |  |
| AP 1, Day 20 |  |  |  |
| AP 1, Day 21 |  |  |  |

Seizures: Please record seizure types and keep a tally of seizures observed

|  | AP 1, Day 15 | AP 1, Day 16 | AP 1, Day 17 | AP 1, Day 18 | AP 1, Day 19 | AP 1, Day 20 | AP 1, Day 21 |
| --- | --- | --- | --- | --- | --- | --- | --- |
| Seizure Type: | | | | | | | |
| Tally |  |  |  |  |  |  |  |
| Total |  |  |  |  |  |  |  |
| Seizure Type: | | | | | | | |
| Tally |  |  |  |  |  |  |  |
| Total |  |  |  |  |  |  |  |
| Seizure Type: | | | | | | | |
| Tally |  |  |  |  |  |  |  |
| Total |  |  |  |  |  |  |  |
| Seizure Type: | | | | | | | |
| Tally |  |  |  |  |  |  |  |
| Total |  |  |  |  |  |  |  |
| Seizure Type: | | | | | | | |
| Tally |  |  |  |  |  |  |  |
| Total |  |  |  |  |  |  |  |
| Seizure Type: | | | | | | | |
| Tally |  |  |  |  |  |  |  |
| Total |  |  |  |  |  |  |  |

# Alternating Therapy Period 1 - Week 4 Date:_____________

Total Dose: _________mg/kg/day. Dose is ______ mL twice per day.

Two doses to be administered each day, within 30 minutes of consuming a main meal (e.g. breakfast or dinner). If a dose is missed DO NOT take twice the amount to make up for the missed dose. Please write ‘Dose Missed’ with a reason why.

| Date | Dose 1 Time | Dose 2 Time | Comments/problems with dose |
| --- | --- | --- | --- |
| **AP 1 - Week 4** | | | |
| AP 1, Day 22 |  |  |  |
| AP 1, Day 23 |  |  |  |
| AP 1, Day 24 |  |  |  |
| AP 1, Day 25 |  |  |  |
| AP 1, Day 26 |  |  |  |
| AP 1, Day 27 |  |  |  |
| AP 1, Day 28 |  |  |  |

Seizures: Please record seizure types and keep a tally of seizures observed

|  | AP 1, Day 22 | AP 1, Day 23 | AP 1, Day 24 | AP 1, Day 25 | AP 1, Day 26 | AP 1, Day 27 | AP 1, Day 28 |
| --- | --- | --- | --- | --- | --- | --- | --- |
| Seizure Type: | | | | | | | |
| Tally |  |  |  |  |  |  |  |
| Total |  |  |  |  |  |  |  |
| Seizure Type: | | | | | | | |
| Tally |  |  |  |  |  |  |  |
| Total |  |  |  |  |  |  |  |
| Seizure Type: | | | | | | | |
| Tally |  |  |  |  |  |  |  |
| Total |  |  |  |  |  |  |  |
| Seizure Type: | | | | | | | |
| Tally |  |  |  |  |  |  |  |
| Total |  |  |  |  |  |  |  |
| Seizure Type: | | | | | | | |
| Tally |  |  |  |  |  |  |  |
| Total |  |  |  |  |  |  |  |
| Seizure Type: | | | | | | | |
| Tally |  |  |  |  |  |  |  |
| Total |  |  |  |  |  |  |  |

# Alternating Therapy Period 2 - Week 1 Date:_____________

Total Dose: _________mg/kg/day. Dose is ______ mL twice per day.

Two doses to be administered each day, within 30 minutes of consuming a main meal (e.g. breakfast or dinner). If a dose is missed DO NOT take twice the amount to make up for the missed dose. Please write ‘Dose Missed’ with a reason why.

| Date | Dose 1 Time | Dose 2 Time | Comments/problems with dose |
| --- | --- | --- | --- |
| AP 2 - Week 1 | | | |
| AP 2, Day 1 |  |  |  |
| AP 2, Day 2 |  |  |  |
| AP 2, Day 3 |  |  |  |
| AP 2, Day 4 |  |  |  |
| AP 2, Day 5 |  |  |  |
| AP 2, Day 6 |  |  |  |
| AP 2, Day 7 |  |  |  |

Seizures: Please record seizure types and keep a tally of seizures observed

|  | AP 2, Day 1 | AP 2, Day 2 | AP 2, Day 3 | AP 2, Day 4 | AP 2, Day 5 | AP 2, Day 6 | AP 2, Day 7 |
| --- | --- | --- | --- | --- | --- | --- | --- |
| Seizure Type: | | | | | | | |
| Tally |  |  |  |  |  |  |  |
| Total |  |  |  |  |  |  |  |
| Seizure Type: | | | | | | | |
| Tally |  |  |  |  |  |  |  |
| Total |  |  |  |  |  |  |  |
| Seizure Type: | | | | | | | |
| Tally |  |  |  |  |  |  |  |
| Total |  |  |  |  |  |  |  |
| Seizure Type: | | | | | | | |
| Tally |  |  |  |  |  |  |  |
| Total |  |  |  |  |  |  |  |
| Seizure Type: | | | | | | | |
| Tally |  |  |  |  |  |  |  |
| Total |  |  |  |  |  |  |  |
| Seizure Type: | | | | | | | |
| Tally |  |  |  |  |  |  |  |
| Total |  |  |  |  |  |  |  |

# Alternating Therapy Period 2 - Week 2 Date:_____________

Total Dose: _________mg/kg/day. Dose is ______ mL twice per day.

Two doses to be administered each day, within 30 minutes of consuming a main meal (e.g. breakfast or dinner). If a dose is missed DO NOT take twice the amount to make up for the missed dose. Please write ‘Dose Missed’ with a reason why.

| Date | Dose 1 Time | Dose 2 Time | Comments/problems with dose |
| --- | --- | --- | --- |
| **AP 2, Week 2** | | | |
| AP 2, Day 8 |  |  |  |
| AP 2, Day 9 |  |  |  |
| AP 2, Day 10 |  |  |  |
| AP 2, Day 11 |  |  |  |
| AP 2, Day 12 |  |  |  |
| AP 2, Day 13 |  |  |  |
| AP 2, Day 14 |  |  |  |

Seizures: Please record seizure types and keep a tally of seizures observed

|  | AP 2, Day 8 | AP 2, Day 9 | AP 2, Day 10 | AP 2, Day 11 | AP 2, Day 12 | AP 2, Day 13 | AP 2, Day 14 |
| --- | --- | --- | --- | --- | --- | --- | --- |
| Seizure Type: | | | | | | | |
| Tally |  |  |  |  |  |  |  |
| Total |  |  |  |  |  |  |  |
| Seizure Type: | | | | | | | |
| Tally |  |  |  |  |  |  |  |
| Total |  |  |  |  |  |  |  |
| Seizure Type: | | | | | | | |
| Tally |  |  |  |  |  |  |  |
| Total |  |  |  |  |  |  |  |
| Seizure Type: | | | | | | | |
| Tally |  |  |  |  |  |  |  |
| Total |  |  |  |  |  |  |  |
| Seizure Type: | | | | | | | |
| Tally |  |  |  |  |  |  |  |
| Total |  |  |  |  |  |  |  |
| Seizure Type: | | | | | | | |
| Tally |  |  |  |  |  |  |  |
| Total |  |  |  |  |  |  |  |

# Alternating Therapy Period 2 - Week 3 Date:_____________

Total Dose: _________mg/kg/day. Dose is ______ mL twice per day.

Two doses to be administered each day, within 30 minutes of consuming a main meal (e.g. breakfast or dinner). If a dose is missed DO NOT take twice the amount to make up for the missed dose. Please write ‘Dose Missed’ with a reason why.

| Date | Dose 1 Time | Dose 2 Time | Comments/problems with dose |
| --- | --- | --- | --- |
| AP 2 - Week 3 | | | |
| AP 2, Day 15 |  |  |  |
| AP 2, Day 16 |  |  |  |
| AP 2, Day 17 |  |  |  |
| AP 2, Day 18 |  |  |  |
| AP 2, Day 19 |  |  |  |
| AP 2, Day 20 |  |  |  |
| AP 2, Day 21 |  |  |  |

Seizures: Please record seizure types and keep a tally of seizures observed

|  | AP 2, Day 15 | AP 2, Day 16 | AP 2, Day 17 | AP 2, Day 18 | AP 2, Day 19 | AP 2, Day 20 | AP 2, Day 21 |
| --- | --- | --- | --- | --- | --- | --- | --- |
| Seizure Type: | | | | | | | |
| Tally |  |  |  |  |  |  |  |
| Total |  |  |  |  |  |  |  |
| Seizure Type: | | | | | | | |
| Tally |  |  |  |  |  |  |  |
| Total |  |  |  |  |  |  |  |
| Seizure Type: | | | | | | | |
| Tally |  |  |  |  |  |  |  |
| Total |  |  |  |  |  |  |  |
| Seizure Type: | | | | | | | |
| Tally |  |  |  |  |  |  |  |
| Total |  |  |  |  |  |  |  |
| Seizure Type: | | | | | | | |
| Tally |  |  |  |  |  |  |  |
| Total |  |  |  |  |  |  |  |
| Seizure Type: | | | | | | | |
| Tally |  |  |  |  |  |  |  |
| Total |  |  |  |  |  |  |  |

# Alternating Therapy Period 2 - Week 4 Date:_____________

Total Dose: _________mg/kg/day. Dose is ______ mL twice per day.

Two doses to be administered each day, within 30 minutes of consuming a main meal (e.g. breakfast or dinner). If a dose is missed DO NOT take twice the amount to make up for the missed dose. Please write ‘Dose Missed’ with a reason why.

| Date | Dose 1 Time | Dose 2 Time | Comments/problems with dose |
| --- | --- | --- | --- |
| **AP 2 - Week 4** | | | |
| AP 2, Day 22 |  |  |  |
| AP 2, Day 23 |  |  |  |
| AP 2, Day 24 |  |  |  |
| AP 2, Day 25 |  |  |  |
| AP 2, Day 26 |  |  |  |
| AP 2, Day 27 |  |  |  |
| AP 2, Day 28 |  |  |  |

Seizures: Please record seizure types and keep a tally of seizures observed

|  | AP 2, Day 22 | AP 2, Day 23 | AP 2, Day 24 | AP 2, Day 25 | AP 2, Day 26 | AP 2, Day 27 | AP 2, Day 28 |
| --- | --- | --- | --- | --- | --- | --- | --- |
| Seizure Type: | | | | | | | |
| Tally |  |  |  |  |  |  |  |
| Total |  |  |  |  |  |  |  |
| Seizure Type: | | | | | | | |
| Tally |  |  |  |  |  |  |  |
| Total |  |  |  |  |  |  |  |
| Seizure Type: | | | | | | | |
| Tally |  |  |  |  |  |  |  |
| Total |  |  |  |  |  |  |  |
| Seizure Type: | | | | | | | |
| Tally |  |  |  |  |  |  |  |
| Total |  |  |  |  |  |  |  |
| Seizure Type: | | | | | | | |
| Tally |  |  |  |  |  |  |  |
| Total |  |  |  |  |  |  |  |
| Seizure Type: | | | | | | | |
| Tally |  |  |  |  |  |  |  |
| Total |  |  |  |  |  |  |  |

# Alternating Therapy Period 3 - Week 1 Date:_____________

Total Dose: _________mg/kg/day. Dose is ______ mL twice per day.

Two doses to be administered each day, within 30 minutes of consuming a main meal (e.g. breakfast or dinner). If a dose is missed DO NOT take twice the amount to make up for the missed dose. Please write ‘Dose Missed’ with a reason why.

| Date | Dose 1 Time | Dose 2 Time | Comments/problems with dose |
| --- | --- | --- | --- |
| AP 3 - Week 1 | | | |
| AP 3, Day 1 |  |  |  |
| AP 3, Day 2 |  |  |  |
| AP 3, Day 3 |  |  |  |
| AP 3, Day 4 |  |  |  |
| AP 3, Day 5 |  |  |  |
| AP 3, Day 6 |  |  |  |
| AP 3, Day 7 |  |  |  |

Seizures: Please record seizure types and keep a tally of seizures observed

|  | AP 3, Day 1 | AP 3, Day 2 | AP 3, Day 3 | AP 3, Day 4 | AP 3, Day 5 | AP 3, Day 6 | AP 3, Day 7 |
| --- | --- | --- | --- | --- | --- | --- | --- |
| Seizure Type: | | | | | | | |
| Tally |  |  |  |  |  |  |  |
| Total |  |  |  |  |  |  |  |
| Seizure Type: | | | | | | | |
| Tally |  |  |  |  |  |  |  |
| Total |  |  |  |  |  |  |  |
| Seizure Type: | | | | | | | |
| Tally |  |  |  |  |  |  |  |
| Total |  |  |  |  |  |  |  |
| Seizure Type: | | | | | | | |
| Tally |  |  |  |  |  |  |  |
| Total |  |  |  |  |  |  |  |
| Seizure Type: | | | | | | | |
| Tally |  |  |  |  |  |  |  |
| Total |  |  |  |  |  |  |  |
| Seizure Type: | | | | | | | |
| Tally |  |  |  |  |  |  |  |
| Total |  |  |  |  |  |  |  |

# Alternating Therapy Period 3 - Week 2 Date:_____________

Total Dose: _________mg/kg/day. Dose is ______ mL twice per day.

Two doses to be administered each day, within 30 minutes of consuming a main meal (e.g. breakfast or dinner). If a dose is missed DO NOT take twice the amount to make up for the missed dose. Please write ‘Dose Missed’ with a reason why.

| Date | Dose 1 Time | Dose 2 Time | Comments/problems with dose |
| --- | --- | --- | --- |
| **AP 3 - Week 2** | | | |
| AP 3, Day 8 |  |  |  |
| AP 3, Day 9 |  |  |  |
| AP 3, Day 10 |  |  |  |
| AP 3, Day 11 |  |  |  |
| AP 3, Day 12 |  |  |  |
| AP 3, Day 13 |  |  |  |
| AP 3, Day 14 |  |  |  |

Seizures: Please record seizure types and keep a tally of seizures observed

|  | AP 3, Day 8 | AP 3, Day 9 | AP 3, Day 10 | AP 3, Day 11 | AP 3, Day 12 | AP 3, Day 13 | AP 3, Day 14 |
| --- | --- | --- | --- | --- | --- | --- | --- |
| Seizure Type: | | | | | | | |
| Tally |  |  |  |  |  |  |  |
| Total |  |  |  |  |  |  |  |
| Seizure Type: | | | | | | | |
| Tally |  |  |  |  |  |  |  |
| Total |  |  |  |  |  |  |  |
| Seizure Type: | | | | | | | |
| Tally |  |  |  |  |  |  |  |
| Total |  |  |  |  |  |  |  |
| Seizure Type: | | | | | | | |
| Tally |  |  |  |  |  |  |  |
| Total |  |  |  |  |  |  |  |
| Seizure Type: | | | | | | | |
| Tally |  |  |  |  |  |  |  |
| Total |  |  |  |  |  |  |  |
| Seizure Type: | | | | | | | |
| Tally |  |  |  |  |  |  |  |
| Total |  |  |  |  |  |  |  |

# Alternating Therapy Period 3 - Week 3 Date:_____________

Total Dose: _________mg/kg/day. Dose is ______ mL twice per day.

Two doses to be administered each day, within 30 minutes of consuming a main meal (e.g. breakfast or dinner). If a dose is missed DO NOT take twice the amount to make up for the missed dose. Please write ‘Dose Missed’ with a reason why.

| Date | Dose 1 Time | Dose 2 Time | Comments/problems with dose |
| --- | --- | --- | --- |
| **AP 3 - Week 3** | | | |
| AP 3, Day 15 |  |  |  |
| AP 3, Day 16 |  |  |  |
| AP 3, Day 17 |  |  |  |
| AP 3, Day 18 |  |  |  |
| AP 3, Day 19 |  |  |  |
| AP 3, Day 20 |  |  |  |
| AP 3, Day 21 |  |  |  |

Seizures: Please record seizure types and keep a tally of seizures observed

|  | AP 3, Day 15 | AP 3, Day 16 | AP 3, Day 17 | AP 3, Day 18 | AP 3, Day 19 | AP 3, Day 20 | AP 3, Day 21 |
| --- | --- | --- | --- | --- | --- | --- | --- |
| Seizure Type: | | | | | | | |
| Tally |  |  |  |  |  |  |  |
| Total |  |  |  |  |  |  |  |
| Seizure Type: | | | | | | | |
| Tally |  |  |  |  |  |  |  |
| Total |  |  |  |  |  |  |  |
| Seizure Type: | | | | | | | |
| Tally |  |  |  |  |  |  |  |
| Total |  |  |  |  |  |  |  |
| Seizure Type: | | | | | | | |
| Tally |  |  |  |  |  |  |  |
| Total |  |  |  |  |  |  |  |
| Seizure Type: | | | | | | | |
| Tally |  |  |  |  |  |  |  |
| Total |  |  |  |  |  |  |  |
| Seizure Type: | | | | | | | |
| Tally |  |  |  |  |  |  |  |
| Total |  |  |  |  |  |  |  |

# Alternating Therapy Period 3 - Week 4 Date:_____________

Total Dose: _________mg/kg/day. Dose is ______ mL twice per day.

Two doses to be administered each day, within 30 minutes of consuming a main meal (e.g. breakfast or dinner). If a dose is missed DO NOT take twice the amount to make up for the missed dose. Please write ‘Dose Missed’ with a reason why.

| Date | Dose 1 Time | Dose 2 Time | Comments/problems with dose |
| --- | --- | --- | --- |
| **AP 3 - Week 4** | | | |
| AP 3, Day 22 |  |  |  |
| AP 3, Day 23 |  |  |  |
| AP 3, Day 24 |  |  |  |
| AP 3, Day 25 |  |  |  |
| AP 3, Day 26 |  |  |  |
| AP 3, Day 27 |  |  |  |
| AP 3, Day 28 |  |  |  |

Seizures: Please record seizure types and keep a tally of seizures observed

|  | AP 3, Day 22 | AP 3, Day 23 | AP 3, Day 24 | AP 3, Day 25 | AP 3, Day 26 | AP 3, Day 27 | AP 3, Day 28 |
| --- | --- | --- | --- | --- | --- | --- | --- |
| Seizure Type: | | | | | | | |
| Tally |  |  |  |  |  |  |  |
| Total |  |  |  |  |  |  |  |
| Seizure Type: | | | | | | | |
| Tally |  |  |  |  |  |  |  |
| Total |  |  |  |  |  |  |  |
| Seizure Type: | | | | | | | |
| Tally |  |  |  |  |  |  |  |
| Total |  |  |  |  |  |  |  |
| Seizure Type: | | | | | | | |
| Tally |  |  |  |  |  |  |  |
| Total |  |  |  |  |  |  |  |
| Seizure Type: | | | | | | | |
| Tally |  |  |  |  |  |  |  |
| Total |  |  |  |  |  |  |  |
| Seizure Type: | | | | | | | |
| Tally |  |  |  |  |  |  |  |
| Total |  |  |  |  |  |  |  |

# Alternating Therapy Period 4 - Week 1 Date:_____________

Total Dose: _________mg/kg/day. Dose is ______ mL twice per day.

Two doses to be administered each day, within 30 minutes of consuming a main meal (e.g. breakfast or dinner). If a dose is missed DO NOT take twice the amount to make up for the missed dose. Please write ‘Dose Missed’ with a reason why.

| Date | Dose 1 Time | Dose 2 Time | Comments/problems with dose |
| --- | --- | --- | --- |
| AP 4 - Week 1 | | | |
| AP 4, Day 1 |  |  |  |
| AP 4, Day 2 |  |  |  |
| AP 4, Day 3 |  |  |  |
| AP 4, Day 4 |  |  |  |
| AP 4, Day 5 |  |  |  |
| AP 4, Day 6 |  |  |  |
| AP 4, Day 7 |  |  |  |

Seizures: Please record seizure types and keep a tally of seizures observed

|  | AP 4, Day 1 | AP 4, Day 2 | AP 4, Day 3 | AP 4, Day 4 | AP 4, Day 5 | AP 4, Day 6 | AP 4, Day 7 |
| --- | --- | --- | --- | --- | --- | --- | --- |
| Seizure Type: | | | | | | | |
| Tally |  |  |  |  |  |  |  |
| Total |  |  |  |  |  |  |  |
| Seizure Type: | | | | | | | |
| Tally |  |  |  |  |  |  |  |
| Total |  |  |  |  |  |  |  |
| Seizure Type: | | | | | | | |
| Tally |  |  |  |  |  |  |  |
| Total |  |  |  |  |  |  |  |
| Seizure Type: | | | | | | | |
| Tally |  |  |  |  |  |  |  |
| Total |  |  |  |  |  |  |  |
| Seizure Type: | | | | | | | |
| Tally |  |  |  |  |  |  |  |
| Total |  |  |  |  |  |  |  |
| Seizure Type: | | | | | | | |
| Tally |  |  |  |  |  |  |  |
| Total |  |  |  |  |  |  |  |

# Alternating Therapy Period 4 - Week 2 Date:_____________

Total Dose: _________mg/kg/day. Dose is ______ mL twice per day.

Two doses to be administered each day, within 30 minutes of consuming a main meal (e.g. breakfast or dinner). If a dose is missed DO NOT take twice the amount to make up for the missed dose. Please write ‘Dose Missed’ with a reason why.

| Date | Dose 1 Time | Dose 2 Time | Comments/problems with dose |
| --- | --- | --- | --- |
| **AP 4 - Week 2** | | | |
| AP 4, Day 8 |  |  |  |
| AP 4, Day 9 |  |  |  |
| AP 4, Day 10 |  |  |  |
| AP 4, Day 11 |  |  |  |
| AP 4, Day 12 |  |  |  |
| AP 4, Day 13 |  |  |  |
| AP 4, Day 14 |  |  |  |

Seizures: Please record seizure types and keep a tally of seizures observed

|  | AP 4, Day 8 | AP 4, Day 9 | AP 4, Day 10 | AP 4, Day 11 | AP 4, Day 12 | AP 4, Day 13 | AP 4, Day 14 |
| --- | --- | --- | --- | --- | --- | --- | --- |
| Seizure Type: | | | | | | | |
| Tally |  |  |  |  |  |  |  |
| Total |  |  |  |  |  |  |  |
| Seizure Type: | | | | | | | |
| Tally |  |  |  |  |  |  |  |
| Total |  |  |  |  |  |  |  |
| Seizure Type: | | | | | | | |
| Tally |  |  |  |  |  |  |  |
| Total |  |  |  |  |  |  |  |
| Seizure Type: | | | | | | | |
| Tally |  |  |  |  |  |  |  |
| Total |  |  |  |  |  |  |  |
| Seizure Type: | | | | | | | |
| Tally |  |  |  |  |  |  |  |
| Total |  |  |  |  |  |  |  |
| Seizure Type: | | | | | | | |
| Tally |  |  |  |  |  |  |  |
| Total |  |  |  |  |  |  |  |

# Alternating Therapy Period 4 - Week 3 Date:_____________

Total Dose: _________mg/kg/day. Dose is ______ mL twice per day.

Two doses to be administered each day, within 30 minutes of consuming a main meal (e.g. breakfast or dinner). If a dose is missed DO NOT take twice the amount to make up for the missed dose. Please write ‘Dose Missed’ with a reason why.

| Date | Dose 1 Time | Dose 2 Time | Comments/problems with dose |
| --- | --- | --- | --- |
| AP 4 - Week 3 | | | |
| AP 4, Day 15 |  |  |  |
| AP 4, Day 16 |  |  |  |
| AP 4, Day 17 |  |  |  |
| AP 4, Day 18 |  |  |  |
| AP 4, Day 19 |  |  |  |
| AP 4, Day 20 |  |  |  |
| AP 4, Day 21 |  |  |  |

Seizures: Please record seizure types and keep a tally of seizures observed

|  | AP 4, Day 15 | AP 4, Day 16 | AP 4, Day 17 | AP 4, Day 18 | AP 4, Day 19 | AP 4, Day 20 | AP 4, Day 21 |
| --- | --- | --- | --- | --- | --- | --- | --- |
| Seizure Type: | | | | | | | |
| Tally |  |  |  |  |  |  |  |
| Total |  |  |  |  |  |  |  |
| Seizure Type: | | | | | | | |
| Tally |  |  |  |  |  |  |  |
| Total |  |  |  |  |  |  |  |
| Seizure Type: | | | | | | | |
| Tally |  |  |  |  |  |  |  |
| Total |  |  |  |  |  |  |  |
| Seizure Type: | | | | | | | |
| Tally |  |  |  |  |  |  |  |
| Total |  |  |  |  |  |  |  |
| Seizure Type: | | | | | | | |
| Tally |  |  |  |  |  |  |  |
| Total |  |  |  |  |  |  |  |
| Seizure Type: | | | | | | | |
| Tally |  |  |  |  |  |  |  |
| Total |  |  |  |  |  |  |  |

# Alternating Therapy Period 4 - Week 4 Date:_____________

Total Dose: _________mg/kg/day. Dose is ______ mL twice per day.

Two doses to be administered each day, within 30 minutes of consuming a main meal (e.g. breakfast or dinner). If a dose is missed DO NOT take twice the amount to make up for the missed dose. Please write ‘Dose Missed’ with a reason why.

| Date | Dose 1 Time | Dose 2 Time | Comments/problems with dose |
| --- | --- | --- | --- |
| **AP 4 - Week 4** | | | |
| AP 4, Day 22 |  |  |  |
| AP 4, Day 23 |  |  |  |
| AP 4, Day 24 |  |  |  |
| AP 4, Day 25 |  |  |  |
| AP 4, Day 26 |  |  |  |
| AP 4, Day 27 |  |  |  |
| AP 4, Day 28 |  |  |  |

Seizures: Please record seizure types and keep a tally of seizures observed

|  | AP 4, Day 22 | AP 4, Day 23 | AP 4, Day 24 | AP 4, Day 25 | AP 4, Day 26 | AP 4, Day 27 | AP 4, Day 28 |
| --- | --- | --- | --- | --- | --- | --- | --- |
| Seizure Type: | | | | | | | |
| Tally |  |  |  |  |  |  |  |
| Total |  |  |  |  |  |  |  |
| Seizure Type: | | | | | | | |
| Tally |  |  |  |  |  |  |  |
| Total |  |  |  |  |  |  |  |
| Seizure Type: | | | | | | | |
| Tally |  |  |  |  |  |  |  |
| Total |  |  |  |  |  |  |  |
| Seizure Type: | | | | | | | |
| Tally |  |  |  |  |  |  |  |
| Total |  |  |  |  |  |  |  |
| Seizure Type: | | | | | | | |
| Tally |  |  |  |  |  |  |  |
| Total |  |  |  |  |  |  |  |
| Seizure Type: | | | | | | | |
| Tally |  |  |  |  |  |  |  |
| Total |  |  |  |  |  |  |  |

# Alternating Therapy Period 5 - Week 1 Date:_____________

Total Dose: _________mg/kg/day. Dose is ______ mL twice per day.

Two doses to be administered each day, within 30 minutes of consuming a main meal (e.g. breakfast or dinner). If a dose is missed DO NOT take twice the amount to make up for the missed dose. Please write ‘Dose Missed’ with a reason why.

| Date | Dose 1 Time | Dose 2 Time | Comments/problems with dose |
| --- | --- | --- | --- |
| AP 5 - Week 1 | | | |
| AP 5, Day 1 |  |  |  |
| AP 5, Day 2 |  |  |  |
| AP 5, Day 3 |  |  |  |
| AP 5, Day 4 |  |  |  |
| AP 5, Day 5 |  |  |  |
| AP 5, Day 6 |  |  |  |
| AP 5, Day 7 |  |  |  |

Seizures: Please record seizure types and keep a tally of seizures observed

|  | AP 5, Day 1 | AP 5, Day 2 | AP 5, Day 3 | AP 5, Day 4 | AP 5, Day 5 | AP 5, Day 6 | AP 5, Day 7 |
| --- | --- | --- | --- | --- | --- | --- | --- |
| Seizure Type: | | | | | | | |
| Tally |  |  |  |  |  |  |  |
| Total |  |  |  |  |  |  |  |
| Seizure Type: | | | | | | | |
| Tally |  |  |  |  |  |  |  |
| Total |  |  |  |  |  |  |  |
| Seizure Type: | | | | | | | |
| Tally |  |  |  |  |  |  |  |
| Total |  |  |  |  |  |  |  |
| Seizure Type: | | | | | | | |
| Tally |  |  |  |  |  |  |  |
| Total |  |  |  |  |  |  |  |
| Seizure Type: | | | | | | | |
| Tally |  |  |  |  |  |  |  |
| Total |  |  |  |  |  |  |  |
| Seizure Type: | | | | | | | |
| Tally |  |  |  |  |  |  |  |
| Total |  |  |  |  |  |  |  |

# Alternating Therapy Period 5 - Week 2 Date:_____________

Total Dose: _________mg/kg/day. Dose is ______ mL twice per day.

Two doses to be administered each day, within 30 minutes of consuming a main meal (e.g. breakfast or dinner). If a dose is missed DO NOT take twice the amount to make up for the missed dose. Please write ‘Dose Missed’ with a reason why.

| Date | Dose 1 Time | Dose 2 Time | Comments/problems with dose |
| --- | --- | --- | --- |
| **AP 5 - Week 2** | | | |
| AP 5, Day 8 |  |  |  |
| AP 5, Day 9 |  |  |  |
| AP 5, Day 10 |  |  |  |
| AP 5, Day 11 |  |  |  |
| AP 5, Day 12 |  |  |  |
| AP 5, Day 13 |  |  |  |
| AP 5, Day 14 |  |  |  |

Seizures: Please record seizure types and keep a tally of seizures observed

|  | AP 5, Day 8 | AP 5, Day 9 | AP 5, Day 10 | AP 5, Day 11 | AP 5, Day 12 | AP 5, Day 13 | AP 5, Day 14 |
| --- | --- | --- | --- | --- | --- | --- | --- |
| Seizure Type: | | | | | | | |
| Tally |  |  |  |  |  |  |  |
| Total |  |  |  |  |  |  |  |
| Seizure Type: | | | | | | | |
| Tally |  |  |  |  |  |  |  |
| Total |  |  |  |  |  |  |  |
| Seizure Type: | | | | | | | |
| Tally |  |  |  |  |  |  |  |
| Total |  |  |  |  |  |  |  |
| Seizure Type: | | | | | | | |
| Tally |  |  |  |  |  |  |  |
| Total |  |  |  |  |  |  |  |
| Seizure Type: | | | | | | | |
| Tally |  |  |  |  |  |  |  |
| Total |  |  |  |  |  |  |  |
| Seizure Type: | | | | | | | |
| Tally |  |  |  |  |  |  |  |
| Total |  |  |  |  |  |  |  |

# Alternating Therapy Period 5 - Week 3 Date:_____________

Total Dose: _________mg/kg/day. Dose is ______ mL twice per day.

Two doses to be administered each day, within 30 minutes of consuming a main meal (e.g. breakfast or dinner). If a dose is missed DO NOT take twice the amount to make up for the missed dose. Please write ‘Dose Missed’ with a reason why.

| Date | Dose 1 Time | Dose 2 Time | Comments/problems with dose |
| --- | --- | --- | --- |
| AP 5 - Week 3 | | | |
| AP 5, Day 15 |  |  |  |
| AP 5, Day 16 |  |  |  |
| AP 5, Day 17 |  |  |  |
| AP 5, Day 18 |  |  |  |
| AP 5, Day 19 |  |  |  |
| AP 5, Day 20 |  |  |  |
| AP 5, Day 21 |  |  |  |

Seizures: Please record seizure types and keep a tally of seizures observed

|  | AP 5, Day 15 | AP 5, Day 16 | AP 5, Day 17 | AP 5, Day 18 | AP 5, Day 19 | AP 5, Day 20 | AP 5, Day 21 |
| --- | --- | --- | --- | --- | --- | --- | --- |
| Seizure Type: | | | | | | | |
| Tally |  |  |  |  |  |  |  |
| Total |  |  |  |  |  |  |  |
| Seizure Type: | | | | | | | |
| Tally |  |  |  |  |  |  |  |
| Total |  |  |  |  |  |  |  |
| Seizure Type: | | | | | | | |
| Tally |  |  |  |  |  |  |  |
| Total |  |  |  |  |  |  |  |
| Seizure Type: | | | | | | | |
| Tally |  |  |  |  |  |  |  |
| Total |  |  |  |  |  |  |  |
| Seizure Type: | | | | | | | |
| Tally |  |  |  |  |  |  |  |
| Total |  |  |  |  |  |  |  |
| Seizure Type: | | | | | | | |
| Tally |  |  |  |  |  |  |  |
| Total |  |  |  |  |  |  |  |

# Alternating Therapy Period 5 - Week 4 Date:_____________

Total Dose: _________mg/kg/day. Dose is ______ mL twice per day.

Two doses to be administered each day, within 30 minutes of consuming a main meal (e.g. breakfast or dinner). If a dose is missed DO NOT take twice the amount to make up for the missed dose. Please write ‘Dose Missed’ with a reason why.

| Date | Dose 1 Time | Dose 2 Time | Comments/problems with dose |
| --- | --- | --- | --- |
| **AP 5 - Week 4** | | | |
| AP 5, Day 22 |  |  |  |
| AP 5, Day 23 |  |  |  |
| AP 5, Day 24 |  |  |  |
| AP 5, Day 25 |  |  |  |
| AP 5, Day 26 |  |  |  |
| AP 5, Day 27 |  |  |  |
| AP 5, Day 28 |  |  |  |

Seizures: Please record seizure types and keep a tally of seizures observed

|  | AP 5, Day 22 | AP 5, Day 23 | AP 5, Day 24 | AP 5, Day 25 | AP 5, Day 26 | AP 5, Day 27 | AP 5, Day 28 |
| --- | --- | --- | --- | --- | --- | --- | --- |
| Seizure Type: | | | | | | | |
| Tally |  |  |  |  |  |  |  |
| Total |  |  |  |  |  |  |  |
| Seizure Type: | | | | | | | |
| Tally |  |  |  |  |  |  |  |
| Total |  |  |  |  |  |  |  |
| Seizure Type: | | | | | | | |
| Tally |  |  |  |  |  |  |  |
| Total |  |  |  |  |  |  |  |
| Seizure Type: | | | | | | | |
| Tally |  |  |  |  |  |  |  |
| Total |  |  |  |  |  |  |  |
| Seizure Type: | | | | | | | |
| Tally |  |  |  |  |  |  |  |
| Total |  |  |  |  |  |  |  |
| Seizure Type: | | | | | | | |
| Tally |  |  |  |  |  |  |  |
| Total |  |  |  |  |  |  |  |

# Alternating Therapy Period 6 - Week 1 Date:_____________

Total Dose: _________mg/kg/day. Dose is ______ mL twice per day.

Two doses to be administered each day, within 30 minutes of consuming a main meal (e.g. breakfast or dinner). If a dose is missed DO NOT take twice the amount to make up for the missed dose. Please write ‘Dose Missed’ with a reason why.

| Date | Dose 1 Time | Dose 2 Time | Comments/problems with dose |
| --- | --- | --- | --- |
| AP 6 - Week 1 | | | |
| AP 6, Day 1 |  |  |  |
| AP 6, Day 2 |  |  |  |
| AP 6, Day 3 |  |  |  |
| AP 6, Day 4 |  |  |  |
| AP 6, Day 5 |  |  |  |
| AP 6, Day 6 |  |  |  |
| AP 6, Day 7 |  |  |  |

Seizures: Please record seizure types and keep a tally of seizures observed

|  | AP 6, Day 1 | AP 6, Day 2 | AP 6, Day 3 | AP 6, Day 4 | AP 6, Day 5 | AP 6, Day 6 | AP 6, Day 7 |
| --- | --- | --- | --- | --- | --- | --- | --- |
| Seizure Type: | | | | | | | |
| Tally |  |  |  |  |  |  |  |
| Total |  |  |  |  |  |  |  |
| Seizure Type: | | | | | | | |
| Tally |  |  |  |  |  |  |  |
| Total |  |  |  |  |  |  |  |
| Seizure Type: | | | | | | | |
| Tally |  |  |  |  |  |  |  |
| Total |  |  |  |  |  |  |  |
| Seizure Type: | | | | | | | |
| Tally |  |  |  |  |  |  |  |
| Total |  |  |  |  |  |  |  |
| Seizure Type: | | | | | | | |
| Tally |  |  |  |  |  |  |  |
| Total |  |  |  |  |  |  |  |
| Seizure Type: | | | | | | | |
| Tally |  |  |  |  |  |  |  |
| Total |  |  |  |  |  |  |  |

# Alternating Therapy Period 6 - Week 2 Date:_____________

Total Dose: _________mg/kg/day. Dose is ______ mL twice per day.

Two doses to be administered each day, within 30 minutes of consuming a main meal (e.g. breakfast or dinner). If a dose is missed DO NOT take twice the amount to make up for the missed dose. Please write ‘Dose Missed’ with a reason why.

| Date | Dose 1 Time | Dose 2 Time | Comments/problems with dose |
| --- | --- | --- | --- |
| **AP 6 - Week 2** | | | |
| AP 6, Day 8 |  |  |  |
| AP 6, Day 9 |  |  |  |
| AP 6, Day 10 |  |  |  |
| AP 6, Day 11 |  |  |  |
| AP 6, Day 12 |  |  |  |
| AP 6, Day 13 |  |  |  |
| AP 6, Day 14 |  |  |  |

Seizures: Please record seizure types and keep a tally of seizures observed

|  | AP 6, Day 8 | AP 6, Day 9 | AP 6, Day 10 | AP 6, Day 11 | AP 6, Day 12 | AP 6, Day 13 | AP 6, Day 14 |
| --- | --- | --- | --- | --- | --- | --- | --- |
| Seizure Type: | | | | | | | |
| Tally |  |  |  |  |  |  |  |
| Total |  |  |  |  |  |  |  |
| Seizure Type: | | | | | | | |
| Tally |  |  |  |  |  |  |  |
| Total |  |  |  |  |  |  |  |
| Seizure Type: | | | | | | | |
| Tally |  |  |  |  |  |  |  |
| Total |  |  |  |  |  |  |  |
| Seizure Type: | | | | | | | |
| Tally |  |  |  |  |  |  |  |
| Total |  |  |  |  |  |  |  |
| Seizure Type: | | | | | | | |
| Tally |  |  |  |  |  |  |  |
| Total |  |  |  |  |  |  |  |
| Seizure Type: | | | | | | | |
| Tally |  |  |  |  |  |  |  |
| Total |  |  |  |  |  |  |  |

# Alternating Therapy Period 6 - Week 3 Date:_____________

Total Dose: _________mg/kg/day. Dose is ______ mL twice per day.

Two doses to be administered each day, within 30 minutes of consuming a main meal (e.g. breakfast or dinner). If a dose is missed DO NOT take twice the amount to make up for the missed dose. Please write ‘Dose Missed’ with a reason why.

| Date | Dose 1 Time | Dose 2 Time | Comments/problems with dose |
| --- | --- | --- | --- |
| AP 6 - Week 3 | | | |
| AP 6, Day 15 |  |  |  |
| AP 6, Day 16 |  |  |  |
| AP 6, Day 17 |  |  |  |
| AP 6, Day 18 |  |  |  |
| AP 6, Day 19 |  |  |  |
| AP 6, Day 20 |  |  |  |
| AP 6, Day 21 |  |  |  |

Seizures: Please record seizure types and keep a tally of seizures observed

|  | AP 6, Day 15 | AP 6, Day 16 | AP 6, Day 17 | AP 6, Day 18 | AP 6, Day 19 | AP 6, Day 20 | AP 6, Day 21 |
| --- | --- | --- | --- | --- | --- | --- | --- |
| Seizure Type: | | | | | | | |
| Tally |  |  |  |  |  |  |  |
| Total |  |  |  |  |  |  |  |
| Seizure Type: | | | | | | | |
| Tally |  |  |  |  |  |  |  |
| Total |  |  |  |  |  |  |  |
| Seizure Type: | | | | | | | |
| Tally |  |  |  |  |  |  |  |
| Total |  |  |  |  |  |  |  |
| Seizure Type: | | | | | | | |
| Tally |  |  |  |  |  |  |  |
| Total |  |  |  |  |  |  |  |
| Seizure Type: | | | | | | | |
| Tally |  |  |  |  |  |  |  |
| Total |  |  |  |  |  |  |  |
| Seizure Type: | | | | | | | |
| Tally |  |  |  |  |  |  |  |
| Total |  |  |  |  |  |  |  |

# Alternating Therapy Period 6 - Week 4 Date:_____________

Total Dose: _________mg/kg/day. Dose is ______ mL twice per day.

Two doses to be administered each day, within 30 minutes of consuming a main meal (e.g. breakfast or dinner). If a dose is missed DO NOT take twice the amount to make up for the missed dose. Please write ‘Dose Missed’ with a reason why.

| Date | Dose 1 Time | Dose 2 Time | Comments/problems with dose |
| --- | --- | --- | --- |
| **AP 6 - Week 4** | | | |
| AP 6, Day 22 |  |  |  |
| AP 6, Day 23 |  |  |  |
| AP 6, Day 24 |  |  |  |
| AP 6, Day 25 |  |  |  |
| AP 6, Day 26 |  |  |  |
| AP 6, Day 27 |  |  |  |
| AP 6, Day 28 |  |  |  |

Seizures: Please record seizure types and keep a tally of seizures observed

|  | AP 6, Day 22 | AP 6, Day 23 | AP 6, Day 24 | AP 6, Day 25 | AP 6, Day 26 | AP 6, Day 27 | AP 6, Day 28 |
| --- | --- | --- | --- | --- | --- | --- | --- |
| Seizure Type: | | | | | | | |
| Tally |  |  |  |  |  |  |  |
| Total |  |  |  |  |  |  |  |
| Seizure Type: | | | | | | | |
| Tally |  |  |  |  |  |  |  |
| Total |  |  |  |  |  |  |  |
| Seizure Type: | | | | | | | |
| Tally |  |  |  |  |  |  |  |
| Total |  |  |  |  |  |  |  |
| Seizure Type: | | | | | | | |
| Tally |  |  |  |  |  |  |  |
| Total |  |  |  |  |  |  |  |
| Seizure Type: | | | | | | | |
| Tally |  |  |  |  |  |  |  |
| Total |  |  |  |  |  |  |  |
| Seizure Type: | | | | | | | |
| Tally |  |  |  |  |  |  |  |
| Total |  |  |  |  |  |  |  |

# Alternating Therapy Period 7 - Week 1 Date:_____________

Total Dose: _________mg/kg/day. Dose is ______ mL twice per day.

Two doses to be administered each day, within 30 minutes of consuming a main meal (e.g. breakfast or dinner). If a dose is missed DO NOT take twice the amount to make up for the missed dose. Please write ‘Dose Missed’ with a reason why.

| Date | Dose 1 Time | Dose 2 Time | Comments/problems with dose |
| --- | --- | --- | --- |
| AP 7 - Week 1 | | | |
| AP 7, Day 1 |  |  |  |
| AP 7, Day 2 |  |  |  |
| AP 7, Day 3 |  |  |  |
| AP 7, Day 4 |  |  |  |
| AP 7, Day 5 |  |  |  |
| AP 7, Day 6 |  |  |  |
| AP 7, Day 7 |  |  |  |

Seizures: Please record seizure types and keep a tally of seizures observed

|  | AP 7, Day 1 | AP 7, Day 2 | AP 7, Day 3 | AP 7, Day 4 | AP 7, Day 5 | AP 7, Day 6 | AP 7, Day 7 |
| --- | --- | --- | --- | --- | --- | --- | --- |
| Seizure Type: | | | | | | | |
| Tally |  |  |  |  |  |  |  |
| Total |  |  |  |  |  |  |  |
| Seizure Type: | | | | | | | |
| Tally |  |  |  |  |  |  |  |
| Total |  |  |  |  |  |  |  |
| Seizure Type: | | | | | | | |
| Tally |  |  |  |  |  |  |  |
| Total |  |  |  |  |  |  |  |
| Seizure Type: | | | | | | | |
| Tally |  |  |  |  |  |  |  |
| Total |  |  |  |  |  |  |  |
| Seizure Type: | | | | | | | |
| Tally |  |  |  |  |  |  |  |
| Total |  |  |  |  |  |  |  |
| Seizure Type: | | | | | | | |
| Tally |  |  |  |  |  |  |  |
| Total |  |  |  |  |  |  |  |

# Alternating Therapy Period 7 - Week 2 Date:_____________

Total Dose: _________mg/kg/day. Dose is ______ mL twice per day.

Two doses to be administered each day, within 30 minutes of consuming a main meal (e.g. breakfast or dinner). If a dose is missed DO NOT take twice the amount to make up for the missed dose. Please write ‘Dose Missed’ with a reason why.

| Date | Dose 1 Time | Dose 2 Time | Comments/problems with dose |
| --- | --- | --- | --- |
| **AP 7 - Week 2** | | | |
| AP 7, Day 8 |  |  |  |
| AP 7, Day 9 |  |  |  |
| AP 7, Day 10 |  |  |  |
| AP 7, Day 11 |  |  |  |
| AP 7, Day 12 |  |  |  |
| AP 7, Day 13 |  |  |  |
| AP 7, Day 14 |  |  |  |

Seizures: Please record seizure types and keep a tally of seizures observed

|  | AP 7, Day 8 | AP 7, Day 9 | AP 7, Day 10 | AP 7, Day 11 | AP 7, Day 12 | AP 7, Day 13 | AP 7, Day 14 |
| --- | --- | --- | --- | --- | --- | --- | --- |
| Seizure Type: | | | | | | | |
| Tally |  |  |  |  |  |  |  |
| Total |  |  |  |  |  |  |  |
| Seizure Type: | | | | | | | |
| Tally |  |  |  |  |  |  |  |
| Total |  |  |  |  |  |  |  |
| Seizure Type: | | | | | | | |
| Tally |  |  |  |  |  |  |  |
| Total |  |  |  |  |  |  |  |
| Seizure Type: | | | | | | | |
| Tally |  |  |  |  |  |  |  |
| Total |  |  |  |  |  |  |  |
| Seizure Type: | | | | | | | |
| Tally |  |  |  |  |  |  |  |
| Total |  |  |  |  |  |  |  |
| Seizure Type: | | | | | | | |
| Tally |  |  |  |  |  |  |  |
| Total |  |  |  |  |  |  |  |

# Alternating Therapy Period 7 - Week 3 Date:_____________

Total Dose: _________mg/kg/day. Dose is ______ mL twice per day.

Two doses to be administered each day, within 30 minutes of consuming a main meal (e.g. breakfast or dinner). If a dose is missed DO NOT take twice the amount to make up for the missed dose. Please write ‘Dose Missed’ with a reason why.

| Date | Dose 1 Time | Dose 2 Time | Comments/problems with dose |
| --- | --- | --- | --- |
| AP 7 - Week 3 | | | |
| AP 7, Day 15 |  |  |  |
| AP 7, Day 16 |  |  |  |
| AP 7, Day 17 |  |  |  |
| AP 7, Day 18 |  |  |  |
| AP 7, Day 19 |  |  |  |
| AP 7, Day 20 |  |  |  |
| AP 7, Day 21 |  |  |  |

Seizures: Please record seizure types and keep a tally of seizures observed

|  | AP 7, Day 15 | AP 7, Day 16 | AP 7, Day 17 | AP 7, Day 18 | AP 7, Day 19 | AP 7, Day 20 | AP 7, Day 21 |
| --- | --- | --- | --- | --- | --- | --- | --- |
| Seizure Type: | | | | | | | |
| Tally |  |  |  |  |  |  |  |
| Total |  |  |  |  |  |  |  |
| Seizure Type: | | | | | | | |
| Tally |  |  |  |  |  |  |  |
| Total |  |  |  |  |  |  |  |
| Seizure Type: | | | | | | | |
| Tally |  |  |  |  |  |  |  |
| Total |  |  |  |  |  |  |  |
| Seizure Type: | | | | | | | |
| Tally |  |  |  |  |  |  |  |
| Total |  |  |  |  |  |  |  |
| Seizure Type: | | | | | | | |
| Tally |  |  |  |  |  |  |  |
| Total |  |  |  |  |  |  |  |
| Seizure Type: | | | | | | | |
| Tally |  |  |  |  |  |  |  |
| Total |  |  |  |  |  |  |  |

# Alternating Therapy Period 7 - Week 4 Date:_____________

Total Dose: _________mg/kg/day. Dose is ______ mL twice per day.

Two doses to be administered each day, within 30 minutes of consuming a main meal (e.g. breakfast or dinner). If a dose is missed DO NOT take twice the amount to make up for the missed dose. Please write ‘Dose Missed’ with a reason why.

| Date | Dose 1 Time | Dose 2 Time | Comments/problems with dose |
| --- | --- | --- | --- |
| **AP 7 - Week 4** | | | |
| AP 7, Day 22 |  |  |  |
| AP 7, Day 23 |  |  |  |
| AP 7, Day 24 |  |  |  |
| AP 7, Day 25 |  |  |  |
| AP 7, Day 26 |  |  |  |
| AP 7, Day 27 |  |  |  |
| AP 7, Day 28 |  |  |  |

Seizures: Please record seizure types and keep a tally of seizures observed

|  | AP 7, Day 22 | AP 7, Day 23 | AP 7, Day 24 | AP 7, Day 25 | AP 7, Day 26 | AP 7, Day 27 | AP 7, Day 28 |
| --- | --- | --- | --- | --- | --- | --- | --- |
| Seizure Type: | | | | | | | |
| Tally |  |  |  |  |  |  |  |
| Total |  |  |  |  |  |  |  |
| Seizure Type: | | | | | | | |
| Tally |  |  |  |  |  |  |  |
| Total |  |  |  |  |  |  |  |
| Seizure Type: | | | | | | | |
| Tally |  |  |  |  |  |  |  |
| Total |  |  |  |  |  |  |  |
| Seizure Type: | | | | | | | |
| Tally |  |  |  |  |  |  |  |
| Total |  |  |  |  |  |  |  |
| Seizure Type: | | | | | | | |
| Tally |  |  |  |  |  |  |  |
| Total |  |  |  |  |  |  |  |
| Seizure Type: | | | | | | | |
| Tally |  |  |  |  |  |  |  |
| Total |  |  |  |  |  |  |  |

# Alternating Therapy Period 8 - Week 1 Date:_____________

Total Dose: _________mg/kg/day. Dose is ______ mL twice per day.

Two doses to be administered each day, within 30 minutes of consuming a main meal (e.g. breakfast or dinner). If a dose is missed DO NOT take twice the amount to make up for the missed dose. Please write ‘Dose Missed’ with a reason why.

| Date | Dose 1 Time | Dose 2 Time | Comments/problems with dose |
| --- | --- | --- | --- |
| AP 8 - Week 1 | | | |
| AP 8, Day 1 |  |  |  |
| AP 8, Day 2 |  |  |  |
| AP 8, Day 3 |  |  |  |
| AP 8, Day 4 |  |  |  |
| AP 8, Day 5 |  |  |  |
| AP 8, Day 6 |  |  |  |
| AP 8, Day 7 |  |  |  |

Seizures: Please record seizure types and keep a tally of seizures observed

|  | AP 8, Day 1 | AP 8, Day 2 | AP 8, Day 3 | AP 8, Day 4 | AP 8, Day 5 | AP 8, Day 6 | AP 8, Day 7 |
| --- | --- | --- | --- | --- | --- | --- | --- |
| Seizure Type: | | | | | | | |
| Tally |  |  |  |  |  |  |  |
| Total |  |  |  |  |  |  |  |
| Seizure Type: | | | | | | | |
| Tally |  |  |  |  |  |  |  |
| Total |  |  |  |  |  |  |  |
| Seizure Type: | | | | | | | |
| Tally |  |  |  |  |  |  |  |
| Total |  |  |  |  |  |  |  |
| Seizure Type: | | | | | | | |
| Tally |  |  |  |  |  |  |  |
| Total |  |  |  |  |  |  |  |
| Seizure Type: | | | | | | | |
| Tally |  |  |  |  |  |  |  |
| Total |  |  |  |  |  |  |  |
| Seizure Type: | | | | | | | |
| Tally |  |  |  |  |  |  |  |
| Total |  |  |  |  |  |  |  |

# Alternating Therapy Period 8 - Week 2 Date:_____________

Total Dose: _________mg/kg/day. Dose is ______ mL twice per day.

Two doses to be administered each day, within 30 minutes of consuming a main meal (e.g. breakfast or dinner). If a dose is missed DO NOT take twice the amount to make up for the missed dose. Please write ‘Dose Missed’ with a reason why.

| Date | Dose 1 Time | Dose 2 Time | Comments/problems with dose |
| --- | --- | --- | --- |
| **AP 8 - Week 2** | | | |
| AP 8, Day 8 |  |  |  |
| AP 8, Day 9 |  |  |  |
| AP 8, Day 10 |  |  |  |
| AP 8, Day 11 |  |  |  |
| AP 8, Day 12 |  |  |  |
| AP 8, Day 13 |  |  |  |
| AP 8, Day 14 |  |  |  |

Seizures: Please record seizure types and keep a tally of seizures observed

|  | AP 8, Day 8 | AP 8, Day 9 | AP 8, Day 10 | AP 8, Day 11 | AP 8, Day 12 | AP 8, Day 13 | AP 8, Day 14 |
| --- | --- | --- | --- | --- | --- | --- | --- |
| Seizure Type: | | | | | | | |
| Tally |  |  |  |  |  |  |  |
| Total |  |  |  |  |  |  |  |
| Seizure Type: | | | | | | | |
| Tally |  |  |  |  |  |  |  |
| Total |  |  |  |  |  |  |  |
| Seizure Type: | | | | | | | |
| Tally |  |  |  |  |  |  |  |
| Total |  |  |  |  |  |  |  |
| Seizure Type: | | | | | | | |
| Tally |  |  |  |  |  |  |  |
| Total |  |  |  |  |  |  |  |
| Seizure Type: | | | | | | | |
| Tally |  |  |  |  |  |  |  |
| Total |  |  |  |  |  |  |  |
| Seizure Type: | | | | | | | |
| Tally |  |  |  |  |  |  |  |
| Total |  |  |  |  |  |  |  |

# Alternating Therapy Period 8 - Week 3 Date:_____________

Total Dose: _________mg/kg/day. Dose is ______ mL twice per day.

Two doses to be administered each day, within 30 minutes of consuming a main meal (e.g. breakfast or dinner). If a dose is missed DO NOT take twice the amount to make up for the missed dose. Please write ‘Dose Missed’ with a reason why.

| Date | Dose 1 Time | Dose 2 Time | Comments/problems with dose |
| --- | --- | --- | --- |
| AP 8 - Week 3 | | | |
| AP 8, Day 15 |  |  |  |
| AP 8, Day 16 |  |  |  |
| AP 8, Day 17 |  |  |  |
| AP 8, Day 18 |  |  |  |
| AP 8, Day 19 |  |  |  |
| AP 8, Day 20 |  |  |  |
| AP 8, Day 21 |  |  |  |

Seizures: Please record seizure types and keep a tally of seizures observed

|  | AP 8, Day 15 | AP 8, Day 16 | AP 8, Day 17 | AP 8, Day 18 | AP 8, Day 19 | AP 8, Day 20 | AP 8, Day 21 |
| --- | --- | --- | --- | --- | --- | --- | --- |
| Seizure Type: | | | | | | | |
| Tally |  |  |  |  |  |  |  |
| Total |  |  |  |  |  |  |  |
| Seizure Type: | | | | | | | |
| Tally |  |  |  |  |  |  |  |
| Total |  |  |  |  |  |  |  |
| Seizure Type: | | | | | | | |
| Tally |  |  |  |  |  |  |  |
| Total |  |  |  |  |  |  |  |
| Seizure Type: | | | | | | | |
| Tally |  |  |  |  |  |  |  |
| Total |  |  |  |  |  |  |  |
| Seizure Type: | | | | | | | |
| Tally |  |  |  |  |  |  |  |
| Total |  |  |  |  |  |  |  |
| Seizure Type: | | | | | | | |
| Tally |  |  |  |  |  |  |  |
| Total |  |  |  |  |  |  |  |

# Alternating Therapy Period 8 - Week 4 Date:_____________

Total Dose: _________mg/kg/day. Dose is ______ mL twice per day.

Two doses to be administered each day, within 30 minutes of consuming a main meal (e.g. breakfast or dinner). If a dose is missed DO NOT take twice the amount to make up for the missed dose. Please write ‘Dose Missed’ with a reason why.

| Date | Dose 1 Time | Dose 2 Time | Comments/problems with dose |
| --- | --- | --- | --- |
| **AP 8 - Week 4** | | | |
| AP 8, Day 22 |  |  |  |
| AP 8, Day 23 |  |  |  |
| AP 8, Day 24 |  |  |  |
| AP 8, Day 25 |  |  |  |
| AP 8, Day 26 |  |  |  |
| AP 8, Day 27 |  |  |  |
| AP 8, Day 28 |  |  |  |

Seizures: Please record seizure types and keep a tally of seizures observed

|  | AP 8, Day 22 | AP 8, Day 23 | AP 8, Day 24 | AP 8, Day 25 | AP 8, Day 26 | AP 8, Day 27 | AP 8, Day 28 |
| --- | --- | --- | --- | --- | --- | --- | --- |
| Seizure Type: | | | | | | | |
| Tally |  |  |  |  |  |  |  |
| Total |  |  |  |  |  |  |  |
| Seizure Type: | | | | | | | |
| Tally |  |  |  |  |  |  |  |
| Total |  |  |  |  |  |  |  |
| Seizure Type: | | | | | | | |
| Tally |  |  |  |  |  |  |  |
| Total |  |  |  |  |  |  |  |
| Seizure Type: | | | | | | | |
| Tally |  |  |  |  |  |  |  |
| Total |  |  |  |  |  |  |  |
| Seizure Type: | | | | | | | |
| Tally |  |  |  |  |  |  |  |
| Total |  |  |  |  |  |  |  |
| Seizure Type: | | | | | | | |
| Tally |  |  |  |  |  |  |  |
| Total |  |  |  |  |  |  |  |

# Additional Page, Use only if Needed Date________________

Total Dose: _________mg/kg/day. Dose is ______ mL twice per day.

Two doses to be administered each day, within 30 minutes of consuming a main meal (e.g. breakfast or dinner). If a dose is missed DO NOT take twice the amount to make up for the missed dose. Please write ‘Dose Missed’ with a reason why.

| Date | Dose 1 Time | Dose 2 Time | Comments/problems with dose |
| --- | --- | --- | --- |
|  |  |  |  |
|  |  |  |  |
|  |  |  |  |
|  |  |  |  |
|  |  |  |  |
|  |  |  |  |
|  |  |  |  |

Seizures: Please record seizure types and keep a tally of seizures observed

| DATE |  |  |  |  |  |  |  |
| --- | --- | --- | --- | --- | --- | --- | --- |
| Seizure Type: | | | | | | | |
| Tally |  |  |  |  |  |  |  |
| Total |  |  |  |  |  |  |  |
| Seizure Type: | | | | | | | |
| Tally |  |  |  |  |  |  |  |
| Total |  |  |  |  |  |  |  |
| Seizure Type: | | | | | | | |
| Tally |  |  |  |  |  |  |  |
| Total |  |  |  |  |  |  |  |
| Seizure Type: | | | | | | | |
| Tally |  |  |  |  |  |  |  |
| Total |  |  |  |  |  |  |  |
| Seizure Type: | | | | | | | |
| Tally |  |  |  |  |  |  |  |
| Total |  |  |  |  |  |  |  |
| Seizure Type: | | | | | | | |
| Tally |  |  |  |  |  |  |  |
| Total |  |  |  |  |  |  |  |

Please ask for additional pages if needed.

**Appendix 7: Statistical model and simulation evaluation**

*Model Specification*

Letting $Y_{A}$ denote the number of seizures that occur in any one treatment period while on active treatment, and $Y_{B}$ the number of seizures on placebo, we assume that these variables follow Poisson distributions, that is, $Y_{A}\sim\mathrm{Poi}(\eta)$ and $Y_{B}\sim\mathrm{Poi}(\mu)$, where *η >* $\text{0}$ and *μ >* $\text{0}$ are the expected seizure counts for the designated observation period. We refer to these means as rates, since the observation period is fixed, and focus on the ratio of the two seizure rates, which may be interpreted as the relative risk reduction, $R=\eta/\mu$. For statistical modelling purposes we define the parameter $\text{δ}$ as the log of the relative risk reduction, so $\ln\left( \text{η} \right)\text{=}\ln\left( \text{μ} \right)\text{-δ}$. The value$\text{δ = }\text{0}$, equivalent to $\text{R = }\text{1}$, indicates equivalence between active and placebo while positive values of $\text{δ}$ indicate a reduction in seizures on active treatment compared with placebo. The parameters $\mu$ and *δ* are unique to each patient and it is reasonable to assume a continuous (smooth) distribution for these across the population of patients. A continuous distribution is assumed partly for mathematical convenience but also because it is unlikely that there are precise “zeros” – given sufficient data, some effect of medication is likely to be discernible even if this is not regarded as clinically relevant.

In this protocol, each patient produces 6 seizure counts in the successive treatment periods. In our analysis model we ignore the time ordering of these values, relying on the alternating treatment allocation to reduce any potential confounding effects of time period to a negligible level. We thus treat the six successive values as if arising independently from the Poisson distributions described above, and use the resulting Poisson likelihood in conjunction with prior distributions for $\mu$ and $\delta$ to derive a Bayesian posterior probability distribution for the parameter of interest, $\delta$. The Bayesian approach produces directly interpretable probabilities regarding the likelihood of benefit of any specified extent (in terms of $\delta$) for each patient, which can be used to decide whether treatment is beneficial compared with the placebo in reducing the number of seizures.

Closed-form calculations of the posterior distribution are not possible, so we use the general Bayesian computation package Stan [http://mc-stan.org/] (via the R package rstan) to produce high-precision numerical estimates of posterior probabilities of interest. Each run (individual dataset) uses 5 chains with 50,000 iterations and 30,000 warm-up iterations.

*Decision rules*

It was recommended that a decision for each patient be based on the estimated posterior probability that their individual value of $\delta$ (equivalent to the relative reduction in seizure rate, R) was greater than a desirable threshold value, under the base-case (optimistic) prior distribution. This required clinicians to agree on the value of *R* that represented the minimal effect that was worth achieving given the costs and potential side-effects of the treatment. Two threshold values were considered: *R* < 0.5 (definitely would adopt treatment) and *R* < 0.8 (some clinicians would adopt but not all).

*Prior distributions*

In order to obtain the posterior distribution of interest, prior distributions must be specified for the parameters $\mu$ and $\delta$. In the present context, these prior distributions are just the distributions of the patient-specific parameters across the population. We assume independence between the parameters. The prior distribution for $\mu$ has relatively little influence on the conclusions with respect to $\delta$ and we used a gamma distribution for its convenience and flexibility. Several specifications were explored but simulation results are presented for two of these: (a) $\mu\sim\gamma\left( 20,2 \right)$, and (b) $\mu\sim\gamma\left( 10,0.5 \right)$, which represent an average (SD) number of seizures of 10 (2.2) and 20 (6.3), respectively, in the placebo arm. These two specifications span a range from highly informative (assuming minimal population variation around the expected count of 10 seizures) to much less informative (reflecting much more uncertainty about each patient’s underlying true seizure frequency).

A range of prior distributions was also considered for the risk reduction parameter $\text{δ}$, with results presented for two relatively extreme choices that might be considered to represent optimistic and pessimistic opinions about the likely value of the treatment, each of these linked to the clinically specified threshold values defined above. The “optimistic” (base case) prior was defined as $\delta\sim N\left( 0.7, 0.81 \right),$which corresponds to assuming that:

- Half the patients will obtain at least a 50% reduction in seizure rates under treatment
- Another 20% obtain between 20%-50% reduction
- The remaining 30% obtain less than 20% reduction.

A second set of calculations was performed under the more pessimistic assumption that the mean of $\delta$ is 0.223, with the same standard deviation, which corresponds to assuming:

- Half the patients will obtain at least a 20% reduction in seizure rates
- 10% obtain between 0%-20% reduction
- The remaining 40% obtain no (or a “negative”) reduction.

*Evaluation of decision rules by simulation*

Simulation experiments were performed to assess the population performance of a range of specific decision criteria under various assumed population (prior) distributions and decision rules. The number of seizures in the control periods for each subject/trial were generated using a Poisson distribution with mean ($\mu$) drawn from either prior distribution (a) or (b) defined above. The number of seizures in the intervention periods were generated using a Poisson distribution with mean defined as $\text{η}\text{ }\text{=}\text{μ} \exp(-\text{δ})$ after drawing a value of $\delta$ from one of the prior distributions specified above. Three decision rules were examined, each of the form “recommend treatment if Pr(*R* < *t*| data) > 0.8”, for the three thresholds *t*=0.8 (at least 20% reduction in seizure rate), *t* = 0.67 (at least 33% reduction in seizure rate) and *t* = 0.5 (50% reduction). For each case, we simulated a hypothetical series of 2000 n-of-1 trials.

Results from the simulations are presented in the tables below, in terms of sensitivity, defined as the proportion of patients in whom we correctly recommend to treat if the true reduction is greater than the threshold of interest (either 20% or 50% reduction), and specificity, defined as the proportion of patients in whom we correctly recommend not to treat if the true reduction is less than the threshold of interest. Results are shown for both prior distributions for the setting in which the clinical preference is to treat at the lower threshold (20% reduction) but only for the pessimistic prior at the higher threshold.

A key result is that under the base case “optimistic” scenario, if we require Pr($R<0.8 | data)$ to be at least 0.8 (80%) in order to declare treatment successful, then we obtain sensitivities of 87% and 91%, and specificities of 91% and 98%, respectively under the two population distributions for $\mu$. This implies, under the second, more realistic, setting that we would miss only about 10% of patients that we would like to identify as benefiting, and would erroneously decide to continue treating only 2% of those who are really not benefiting. In contrast, decision rules that require a high probability that the patient has achieved a 50% or greater reduction in seizures (third row in each of the tables) exhibit low sensitivity in the population, i.e. are likely to result in failing to identify many patients who are actually benefiting. Another way of expressing this is that if it is believed that the patient who should continue to receive the treatment is one who experiences at least a 50% reduction in seizures, and we wish to optimise the “capture rate” of such children, then we should do this by applying a threshold further down the scale (than 50%). Therefore under the “pessimistic” assumptions, the recommended decision rule is to use the second row in the tables below, i.e. to classify the patient as a treatment responder if Pr($R<0.67 | data)>0.80$.

*Recommended decision rule*

Based on the results of this simulation study, a decision rule requiring Pr($R<0.8)>0.80$ is recommended to define a treatment success for clinicians who would use the treatment when its true effect is a reduction of at least 20% in seizure rate, while Pr($R<0.8)>0.67$ is recommended for clinicians who would only use the treatment when its true effect is a reduction of at least 50%. These decision rules should provide an acceptable balance between false positives and false negatives.

These recommendations have been implemented within the agreed reporting framework (see Appendix 8 for sample report provided to treating clinicians). To try to minimise potential confusion, we display probabilities relating to the 3 thresholds shown in the tables below, while giving just two recommendations, according to the clinician’s preferred treatment threshold, i.e. treatment warranted at 20% reduction (*R* < 0.8) or 50% reduction (*R* < 0.5).

***Tables displaying sensitivity and specificity under simulated scenarios as described above***

1. Assuming population distribution if untreated is $\mu\sim\gamma\left( 20,2 \right)$
   (baseline “conservative” scenario, average = 10 per period)

|  | Optimistic prior;  treat if *R <* 0.8 | | Pessimistic prior;  treat if *R <* 0.8 | | Pessimistic prior;  treat if *R <* 0.5 | |
| --- | --- | --- | --- | --- | --- | --- |
| Decision rule | Sens | Spec | Sens | Spec | Sens | Spec |
| Pr($R<0.8)>0.80$ | 0.87 | 0.95 | 0.80 | 0.98 | 0.96 | 0.84 |
| Pr($R<0.67)>0.80$ | 0.77 | 0.99 | 0.66 | 1.00 | 0.89 | 0.92 |
| Pr($R<0.5)>0.80$ | 0.56 | 1.00 | 0.42 | 1.00 | 0.62 | 0.98 |

1. Assuming population distribution if untreated is $\mu\sim\gamma\left( 10,0.5 \right)$
   (more realistic, ‘non-informative’ for the mean, average = 20 per period)

|  | Optimistic prior;  treat if *R <* 0.8 | | Pessimistic prior;  treat if *R <* 0.8 | | Pessimistic prior;  treat if *R <* 0.5 | |
| --- | --- | --- | --- | --- | --- | --- |
| Decision rule | Sens | Spec | Sens | Spec | Sens | Spec |
| Pr($R<0.8)>0.80$ | 0.91 | 0.98 | 0.84 | 0.97 | 1.00 | 0.81 |
| Pr($R<0.67)>0.80$ | 0.80 | 0.99 | 0.69 | 0.99 | 0.93 | 0.90 |
| Pr($R<0.5)>0.80$ | 0.60 | 1.00 | 0.45 | 1.00 | 0.72 | 0.99 |

**Appendix 8: Example results reporting format**

Single Patient Therapy Plan (N of 1) for Use of Cannabidiol in Paediatric Epilepsy

Report generated 05/12/2018

Clinical Epidemiology & Biostatistics Unit (CEBU), MCRI

**Patient ID:** AUS-TEST-006

**DOB:** 15/12/2005

**Initials:** ABC

**Patient seizure data: A=Active, B=Placebo**

**12**

**10**

**Seizure count**

**8**

**6**

**4**

**2**

**0**

**A1 B1 B2 A2 A3 B3**

**Interpretation***

**Treatment threshold (decision rule) Continued treatment**

**20% reduction in seizure numbers Recommended**

**50% reduction in seizure numbers Not recommended**

1. https://www.tga.gov.au/accessing-unapproved-products [↑](#footnote-ref-1)
2. Punja S, Eslick I, Duan N, Vohra S, the DEcIDE Methods Center N-of-1 Guidance Panel. An Ethical Framework for N-of-1 Trials: Clinical Care, Quality Improvement, or Human Subjects Research? In: Kravitz RL, Duan N, eds and the DEcIDE Methods Center N-of-1 Guidance Panel (Duan N, Eslick I, Gabler NB, Kaplan HC, Kravitz RL, Larson EB, Pace WD, Schmid CH, Sim I, Vohra S). Design and [↑](#footnote-ref-2)
3. Devinsky O, Marsh E, Friedman D, Thiele E et al. Cannabidiol in patients with treatment-resistant epilepsy: an open-label intervention trial. Lancet Neurol. 2016; 15(3):270-278. [↑](#footnote-ref-3)
4. Porter BE, Jacobson C. Report of a parent survey of cannabidiol-enriched cannabis use in pediatric treatment-resistant epilepsy. Epilepsy Behav. 2013; 29:574-577. [↑](#footnote-ref-4)
5. Devinsky O, Marsh E, Friedman D, Thiele E et al. Cannabidiol in patients with treatment-resistant epilepsy: an open-label intervention trial. Lancet Neurol. 2016; 15(3):270-278. [↑](#footnote-ref-5)
6. Porter BE, Jacobson C. Report of a parent survey of cannabidiol-enriched cannabis use in pediatric treatment-resistant epilepsy. Epilepsy Behav. 2013; 29:574-577. [↑](#footnote-ref-6)
7. Epidiolex Product Information: <https://www.accessdata.fda.gov/drugsatfda_docs/label/2018/210365lbl.pdf> (accessed 26 July 2018) [↑](#footnote-ref-7)
8. Punja S, Eslick I, Duan N, Vohra S, the DEcIDE Methods Center N-of-1 Guidance Panel. An Ethical Framework for N-of-1 Trials: Clinical Care, Quality Improvement, or Human Subjects Research? In: Kravitz RL, Duan N, eds and the DEcIDE Methods Center N-of-1 Guidance Panel (Duan N, Eslick I, Gabler NB, Kaplan HC, Kravitz RL, Larson EB, Pace WD, Schmid CH, Sim I, Vohra S). Design and Implementation of N-of-1 Trials: A User’s Guide. AHRQ Publication No. 13(14)-EHC122-EF. Rockville, MD: Agency for Healthcare Research and Quality; February 2014: Chapter 2, pp. 13-22. [↑](#footnote-ref-8)
